# AdamiForge: a modular reverse genetics tool for human adenovirus type 12 (HAdV-A12)

**DOI:** 10.1128/spectrum.00696-26

**Published:** 2026-05-21

**Authors:** Maxence Collard, Jean-François Antoine, Nicolas Debortoli, Céline Maschietto, Jonathan Degosserie, Laurent Gillet, Nicolas A. Gillet

**Affiliations:** 1 Integrated Veterinary Research Unit (URVI), University of Namur, Namur Research Institute for Life Sciences (NARILIS), Namur, Belgium; 2 Department of Laboratory Medicine, Université catholique de Louvain, CHU UCL Namur, Yvoir, Belgium; 3 Namur Molecular Tech, CHU UCL Namur 82470 , Yvoir, Belgium; 4 Department of Infectious and Parasitic Diseases, Immunology-Vaccinology, Faculty of Veterinary Medicine – FARAH, University of Liège 706606, Liège, Belgium; University of Miami, Miami, Florida, USA

**Keywords:** HAdV-A12, adenovirus A12, *Mastadenovirus adami*, recombinant adenovirus, AdamiForge, reverse genetics

## Abstract

Human adenoviruses (HAdVs) are a large family of viruses comprising more than 100 genotypes distributed across seven species (A to G). Depending on the genotype, they cause respiratory, ocular, urinary tract, or gastrointestinal infections. Despite the considerable diversity within the human adenovirus genus, most of our current knowledge comes from studies on HAdV-C5. The availability of experimental tools, including antibodies and reverse genetics systems, for the study of adenoviruses other than the C5 strain remains very limited. Here, we describe a reverse genetics tool to engineer HAdV-A12, named AdamiForge. HAdV-A12 is phylogenetically distant from HAdV-C5 and differs markedly in its clinical manifestations, warranting the development of dedicated experimental tools. In principle, AdamiForge is similar to the widely used reverse genetics tool for HAdV-C5 known as AdenoBuilder. Briefly, the viral genome is divided into several blocks and cloned into high-copy plasmids. These genomic fragments are released using a restriction enzyme and recombined *in vitro* by Gibson assembly. The reconstituted genome is then delivered into permissive cells to generate new virions. The main advantage of this approach lies in the fragmentation of the viral genome into small plasmids, which can be easily modified. In addition to the reconstitution of the wild-type virus, we engineered two recombinant viruses expressing modified versions of the viral DNA-binding protein fused either to eGFP or to a FLAG-tag. These modifications compensated for the lack of specific antibodies and enabled successful detection of the HAdV-A12 DBP by both Western blotting and immunofluorescence.

**IMPORTANCE:**

Adenoviruses belong to a large family of viruses comprising seven species (A–G) and more than 100 genotypes. Beyond their role as human pathogens, adenoviruses have been extensively studied as vectors in gene therapy, vaccine development, and oncolytic virotherapy. To date, research has largely focused on a limited number of strains. However, strain-specific characteristics can substantially influence both pathogenic potential and vector performance. It is therefore essential to expand research to other genotypes and to develop new tools for the study of less-characterized strains such as human adenovirus A12.

## INTRODUCTION

Human adenoviruses (HAdVs) are non-enveloped double-stranded DNA viruses. HAdVs are very diverse, counting more than 100 genotypes spread over seven species (from A to G). They cause upper or lower respiratory tract, ocular, urinary tract, or gastrointestinal infections depending on the genotype. The most common genotypes detected differ among countries and change over time (1). In the United States, HAdV-B3, B7, B14, C1, C2, C5, and E4 are the most frequently reported genotypes, accounting for about 90% of all types reported (2). Although generally self-limiting in adults, HAdVs can lead to acute and lethal infections in early childhood and in immunocompromised individuals (1, 3). Following the primary infection, adenoviruses can establish a persistent latent infection in mucosae-associated lymphocytes and reactivate when the immune system is weakened (4, 5). This characteristic makes adenoviruses a worrying medical problem in the context of organ transplantation (1). Beyond their pathogenic role, HAdVs are valuable biotechnological tools due to their large genome (~35 kb), their ability to infect a wide range of cell types, and their ability to efficiently deliver recombinant DNA into the nucleus. Recombinant adenoviruses are used in both *in vitro* and *in vivo* settings, notably for gene therapy, vaccine, and oncolytic virotherapy (6–8). Most of the recombinant adenoviruses classically used for clinical applications are based on a few genotypes: HAdV-C5, HAdV-D26, or the simian adenovirus ChAdY25 (9–11).

Despite the great diversity within the human adenovirus genus, most of our knowledge about adenoviruses comes from the study of the C5 strain. HAdV-C5 is a common respiratory strain with a median seroprevalence of approximately 70% in the general population (12). Thus, many research tools, such as specific antibodies and a vast collection of mutants, have been developed to allow its study. Recently, the construction of recombinant viruses based on the C5 strain became easier thanks to a reverse genetics tool called AdenoBuilder (13, 14). A reverse genetics tool is an experimental system that enables the reconstruction of viral particles from a known viral genome sequence. The genome, cloned into a plasmid, can be deliberately modified through mutations, deletions, or insertions. This approach is termed “reverse genetics” because it begins with defined genetic changes and examines the resulting phenotype. Because adenoviruses have a large genome, the viral genome is split into different blocks and cloned into high-copy plasmids. The different genomic fragments are released from the plasmids using a restriction enzyme and recombined *in vitro* by Gibson assembly. The reconstituted genome is then delivered into permissive cells to assemble new virions. The main advantage of this process lies in the fragmentation of the viral genome into small plasmids, which allows them to be easily modified. The AdenoBuilder platform was further enriched by the addition of viral proteins fused with fluorescent tags for studying viral gene regulation and replication dynamics in real time (15, 16).

HAdV-A12 is an uncommon cause of classical adenovirus disease and is only rarely isolated from clinical specimens; when identified, it is generally linked to respiratory or enteric infections. The seroprevalence of species A adenoviruses, and of HAdV-A12 in particular, varies considerably from one study to another, ranging from very high to very low prevalence (17–20). The prototype A12 virus (Huie strain) was isolated from a stool sample of a child exhibiting symptoms suggestive of poliomyelitis in the 1950s (21). Phylogenetic analysis shows that species A are closer to the species F and G (responsible for gastroenteritis) than to other species (22). Species A adenoviruses use the CAR receptor for entry (23, 24). If relatively few studies were conducted on species A, some were key for the adenovirus field of research. In the 1960s, species A adenoviruses were shown to be able to induce tumors in animal models, a property that was not shared with C5 (25–30). Despite that, the panel of available tools to study adenoviruses species A remains very limited. Our understanding of HAdV-A12 is still incomplete, and much of what we know has been extrapolated from studies on other adenovirus strains. It is therefore necessary to develop research tools for understudied strains such as HAdV-A12.

To date, the generation of recombinant adenoviruses has relied on large plasmids and laborious cloning strategies. The method involves the modification of full-length adenoviral genomes through homologous recombination in bacteria, followed by delivery of the recombinant genome into packaging cells to generate new mutant viruses. Although functional, these technologies require advanced technical skills and considerable time and resources (31, 32). In addition, working with such long plasmids increases the risk of unwanted modifications during the cloning process. We have therefore developed a new reverse genetics tool for the A12 strain named “AdamiForge,” in reference to the species name of HAdV-A12: *Mastadenovirus adami*. The concept underlying AdamiForge is not novel; rather, it represents an adaptation of the modular AdenoBuilder system to the HAdV-A12 strain. The partitioning of the A12 genome into seven “blocks” allows individual viral genes to be easily manipulated in isolation and then recombined with other mutations or transgenes. In addition to the reconstitution of a wild-type HAdV-A12, we constructed two recombinant viruses, one encoding a DNA-binding protein (DBP) fused with a FLAG-tag and one encoding a DBP fused with eGFP.

## MATERIALS AND METHODS

### Cell lines and virus

All cell lines were maintained in Dulbecco’s Modified Eagle’s medium (DMEM) (Gibco) supplemented with 10% fetal calf serum (FCS) (Gibco), 10 mM L-glutamine, and 1% penicillin/streptomycin (Gibco). All cell lines were maintained in a humidified incubator at 37°C and 5% CO_2_. HEK293T were sourced from American Type Culture Collection (ATCC). A549-V are human lung carcinoma cells engineered to constitutively express protein V of parainfluenza virus type 5. This modification allows the inhibition of IFN signaling through STAT1 degradation and promotes increased viral production. A549 PIV5-V cells were kindly provided by Prof. Niklas Arnberg (Umeå University). HAdV-A12 was obtained from ATCC (VR-863).

### Viral stock production

Viral stocks were amplified in A549 PIV5-V cells. During viral stock production, A549 PIV5-V cells were cultured in OPTI-MEM Reduced-Serum Medium without phenol red (Opti-MEM) (Thermo Fisher Scientific). Five days after infection, when cells showed a clear cytopathic effect, the supernatant was collected, clarified by centrifugation at 3,000 × *g* for 15 min, and set aside. The cells were scraped, centrifuged at 3,000 × *g* for 15 min, and the pellet was resuspended in 2 mL of culture medium. The cell suspension was subjected to three cycles of freeze/thaw to release virions from the cells and clarified by centrifugation at 3,000 × *g* for 15 min. Both clarified fractions containing the virions were pooled and treated with Benzonase (1 U/mL) (Sigma-Aldrich) at 37°C for 30 min to degrade free nucleic acids. After incubation, the virion-containing medium was filtered through a 0.22 µm filter. Viruses were concentrated and purified using a 15 mL Amicon Ultra Centrifugal Filter Unit with 100 kDa molecular weight cut-off (Merck Millipore). Finally, virions were aliquoted and stored at −80°C for further use. Handling of human adenovirus was done in a BSL-2 laboratory.

### Amplification of HAdV-A12 genomic DNA segments and plasmid construction

**Fig 1:**
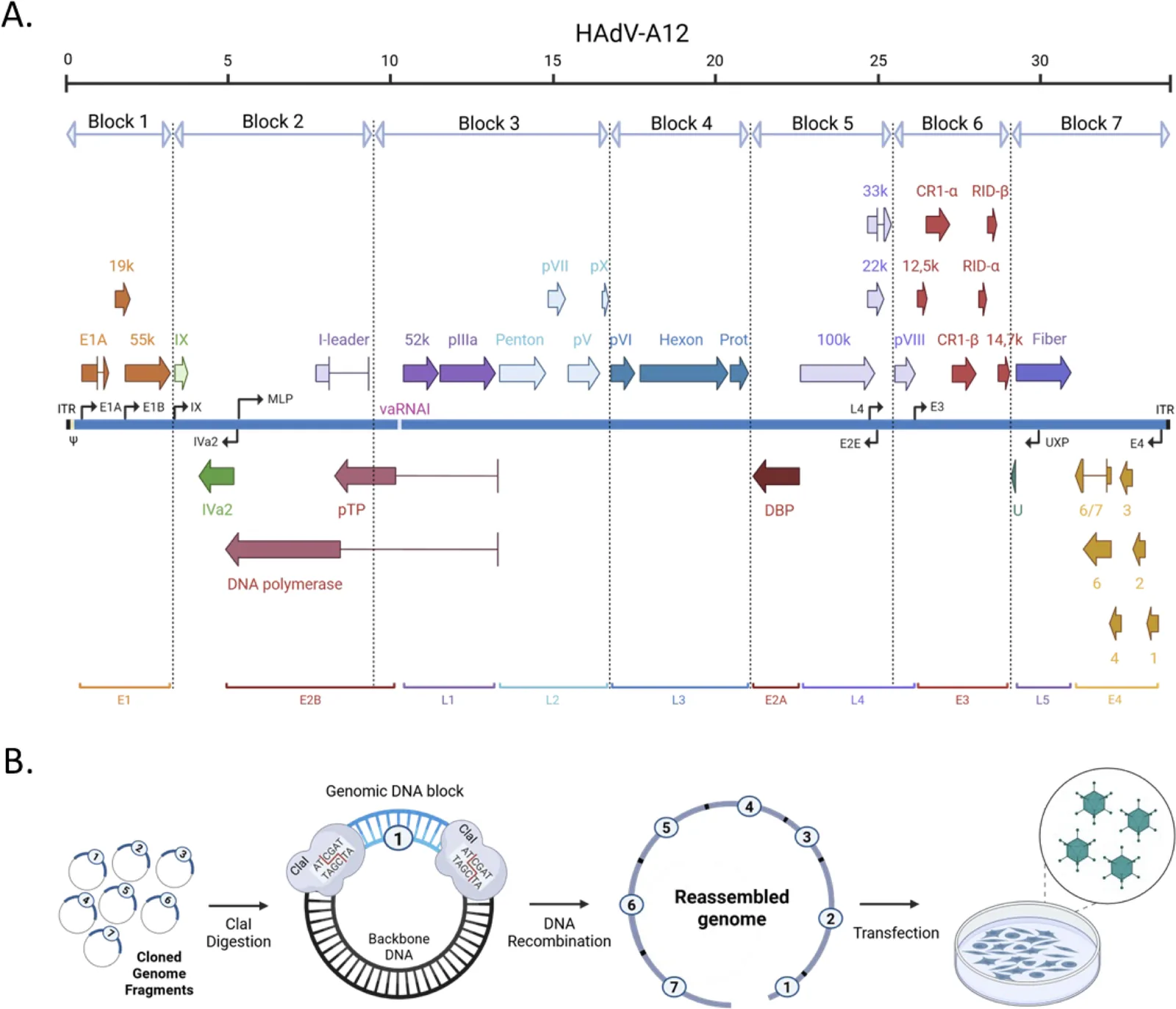
Modular design of AdamiForge. (**A**) The genome of HAdV-A12 is segmented into seven blocks. Each block has been individually cloned to enable easy modification of specific regions of the viral genome. Genes are mapped onto the HAdV-A12 genome and colored according to their transcription unit. Yellow/red colors represent early genes, green colors represent intermediate genes, and blue/purple colors represent late genes. Black arrows represent promoters. Inverted terminal repeat (ITR), packaging sequence (ψ), and vaRNAI are represented on the genome. (**B**) Cloned plasmids can be digested with ClaI restriction enzyme to release viral genome fragments, which are then recombined by Gibson assembly. The full-length genome is then transfected into permissive cells to produce new virions.

HAdV-A12 virions (amplified from ATCC VR-863) were inactivated at 75°C for 15 min. Viral DNA was extracted using NucleoSpin Virus (Macherey-Nagel) according to the manufacturer’s instructions. Extracted DNA was used as a template for PCR amplification of seven overlapping genomic segments covering the entire HAdV-A12 genome (Fig. 1). A sequence corresponding to the ClaI restriction site (ATCGAT) was added upstream of the overlapping regions to allow DNA segments to be released from its vector. Amplification was performed using 1 ng of purified DNA using NEB Q5 High-Fidelity DNA polymerase with the addition of Q5 high-GC enhancer. Genome segmentation was conceived to achieve a balance between functional regions and reasonable segment size (ranging from 3,316 to 7,358 bp). PCR products were purified using Macherey-Nagel NucleoSpin Gel and PCR Clean-up kit according to the manufacturer’s instructions. For each construction, 0.15 pmol of purified DNA was ligated into 0.05 pmol of pJET1.2 blunt-end cloning vector using the CloneJET PCR Cloning kit and according to the manufacturer’s instructions. After a 30 min incubation, 5 µL of ligation mix was used to transform NEB 5-alpha Competent *Escherichia coli* (New England Biolabs). Bacteria were incubated on ice for 30 min with the ligation mix, submitted to a 42°C heat shock for 30 s, and then incubated on ice for 1 min. Bacteria were then resuspended in 200 µL of pre-warmed LB medium for 30 min at 37°C with agitation. After incubation, bacteria were centrifuged at 4,000 × *g* for 2 min and the supernatant removed until 100 µL remained. The bacteria suspension was plated onto Petri dishes containing 0.1% ampicillin solid LB medium. Petri dishes were incubated at 37°C for 24 h. Grown colonies were PCR-screened, and plasmids from positive bacteria were extracted to be Sanger sequenced. Selected plasmids were fully sequenced using Nanopore technology. Plasmids coding for recombinant proteins were engineered from the corresponding wild-type block (no. 5) plasmid using NEBuilder HiFi DNA Assembly Kit (New England Biolabs). Both backbone sequence and inserted DNA were PCR amplified using Q5 High-Fidelity and as previously described. PCR products were purified using Macherey-Nagel NucleoSpin Gel and PCR Clean-up kit according to the manufacturer’s instructions. Homologous recombination was performed using 0.100 pmol of insert DNA, 0.050 pmol of backbone DNA, 10 µL of NEBuilder HiFi DNA Assembly Master Mix, and water was adjusted to 20 µL total. The mixture was incubated for 1 h at 50°C, and the assembly was transformed into competent NEB 5-alpha Competent *E. coli* as previously described. Primer list and PCR thermal protocol are listed in Table S1.

### Viral genome reassembly and production of recombinant virions

A mixture containing 500 fmol of each of the seven blocks (or the corresponding recombinant plasmids) was prepared. The solution was digested using 5 µL of ClaI restriction enzyme (50 units) (New England Biolabs), 10 µL of rCutsmart buffer, and water was adjusted to 100 µL. The digestion mix was incubated at 37°C for 1 h. Digested DNA was precipitated using 10 µL of sodium acetate (3 M pH 5.5) and 400 µL of 100% ethanol. The mix was vortexed for 15 s. A white precipitate should appear. The DNA was then incubated for 1 h at −80°C. After incubation, the DNA was centrifuged at 20,000 × *g* for 15 min. The supernatant was discarded, and the pellet washed with 600 µL of 70% ethanol. DNA was centrifuged once again at 20,000 × *g* for 10 min. The supernatant was discarded, and the pellet dried at 37°C for 3 min (or until all ethanol had evaporated). DNA was resuspended in 10 µL of water and incubated at 37°C for 30 min to allow the DNA to be fully dissolved. Once the pellet was resuspended, 10 µL of NEBuilder HiFi DNA Assembly Master Mix (New England Biolabs) was added and incubated for 1 h at 45°C. The mixture was either directly used for transfection or stored at −20°C. For genome reassembly visualization, 5 µL of the mixture was loaded on a 0.5% agarose gel and ran for 18 h at 20 V.

The assembled genome was delivered using linear cationic polymer PEI MAX (Polysciences). Two mixes were prepared: one mix including 250 µL of Opti-MEM and 50 µg of PEI MAX, and one mix including 250 µL of Opti-MEM and 20 µL of the previously reassembled DNA. Both were gently vortexed and incubated for 5 min at RT. After incubation, the DNA mix was slowly added to the PEI mix, gently flicked, and incubated for 30 min at RT. The day before, HEK293T were previously seeded at 70% confluency in a T25 flask with 10% FCS DMEM. Before transfection, the media was renewed and changed to 6% FCS DMEM, and the solution was finally delivered to the packaging cells.

Seven days post-transfection, viruses were harvested by scraping the T25 flask. Media and cell lysate were gathered and centrifuged at 3,000 × *g* for 15 min. After centrifugation, the supernatant was discarded until 2 mL was left, and the cell pellet was resuspended in the virions-containing media. The lysate was vortexed for 15 s and submitted to three cycles of freeze/thawing with 15 s of vortexing between each cycle. The sample was clarified by centrifugation at 3,000 × *g* for 15 min. The virions-containing media was collected and used to amplify the virus.

One day prior to the collection of the transfected cells, A549 PIV5-V cells were seeded in a six-well plate at 70% confluency. The following day, the culture media was replaced with 2 mL of the virions-containing media. The culture was kept for 10 days or until clear lytic plaques appeared. After the first round of amplification, the viruses were harvested as previously described and further amplified in A549 PIV5-V cells.

If using the eGFP-DPB construct, the user should observe a fair proportion of GFP-positive 293T cells after transfection of the reassembly mix. Despite that, the first passage in A549 PIV5-V cells would only lead to very few replication plaques. This is expected and suggests that only a limited fraction of the initially GFP-positive 293T cells actually harbored a fully recombined, replication-competent viral genome. No screening step is required; the first passage in A549 PIV5-V cells acts as a selection step, only retaining fully recombined, replication-competent viral genomes. It is reasonable to assume that such a bottleneck is also present for other constructions (although less easily visible) and that, therefore, several amplification cycles in the A549 PIV5-V cells are required to achieve high-titer production. Upon recovery of the recombinant virus, viral genome sequencing is recommended to verify the integrity of the construct.

### Titration of infectious viral particles

Infectious viral particles were titrated by tissue culture infection dose 50 assay. A549 PIV5-V cells were seeded in a 96-well plate at 80% confluency 1 day prior to the assay. One day later, the media was renewed with 2% FCS DMEM. Virions were serially diluted 10 by 10 and used to infect the 96-well plate. Cytopathic effects were monitored for 10 days.

### Western blot

After 2 days of infection at MOI = 1, cells were scraped and collected in RIPA buffer (150 mM NaCl, 5 mM EDTA, 50 mM Tris-HCl [pH 8.0], 1% NP-40, 0.5% sodium deoxycholate, 0.1% SDS, 1% Triton X-100) supplemented with 1 mM phenylmethylsulfonyl fluoride and cOmplete protease inhibitor cocktail (Roche). Cells were sonicated for five cycles, 30 s ON/30 s OFF, using a Bioruptor Pico device (Diagenode) at 4°C. Lysate was then centrifuged at 14,000 × *g* for 15 min to remove cell debris. Proteins were quantified using Pierce BCA protein assay kit (Thermo Fisher Scientific). Extracted proteins (35 µg) were loaded in a 10% SDS-PAGE gel (100 mA, 2 h) and transferred to a polyvinylidene difluoride membrane (200 mA, 1 h 30) (GE Healthcare Life Sciences). The membrane was blocked for 1 h in TBS (Tris-buffered saline) supplemented with 0.1% Tween 20 and blocking agent (5% bovine serum albumin [BSA] or 5% milk) (TBS-T BSA/milk). Viral structural proteins were detected with rabbit antibody (1:10,000 in TBS-T 5% BSA) (kindly gifted by Prof. Thomas Dobner). Flagged proteins were detected using mouse anti-FLAG M2 antibody (1:5,000 in TBS-T 5% milk) (Sigma-Aldrich). GFP-fused proteins were detected using mouse anti-GFP 4B10 (1:2,000 in TBS-T milk) (Cell Signaling). Primary antibodies were labeled using goat anti-rabbit immunoglobulins/HRP (1:2,000 in TBS-T 5% BSA) (Dako) (structural proteins) or goat anti-mouse immunoglobulins/HRP (1:2,000 in TBS-T 5% milk) (Dako) (GFP and FLAG). Signal was revealed using Pierce ECL Western Blotting Substrate (Thermo Fisher Scientific), and chemiluminescence was read using ImageQuant LAS 4000 (GE Healthcare Life Sciences).

### Viral genome comparison by diagnostic enzyme restriction test

Purified DNA from cells infected with ATCC HAdV-A12 or recombinant HAdV-A12 was digested using 20 units of BstBI or EcoRV restriction enzymes (New England Biolabs). After 30 min of digestion, DNA was loaded in 1% agarose gel and run through electrophoresis for 1 h at 100 V.

### Viral genome sequencing

Next-generation sequencing libraries were prepared from purified DNA extracted from cells infected with ATCC HAdV-A12 or recombinant HAdV-A12 using Illumina DNA Prep kit (Illumina, San Diego, CA, USA; Cat. No. 20060059). Library indexing was performed by PCR amplification using Q5U Hot Start High-Fidelity DNA Polymerase (New England Biolabs, Ipswich, MA, USA; Cat. No. M0515), following the manufacturer’s recommendations to incorporate unique dual indexes (Illumina, San Diego, CA, USA; Cat. No. 20091660). Libraries were sequenced on an Illumina MiSeq platform using the v2 chemistry (300-cycle kit), generating 2 × 150 bp paired-end reads.

Raw sequencing reads were processed using a custom bioinformatics pipeline. Read quality was initially assessed using FastQC. Adapter sequences and low-quality bases were trimmed using fastp, and reads failing quality thresholds were removed. To eliminate host-derived sequences, reads were mapped with BWA-MEM to the human reference genome (GRCh38), and unmapped reads were retained for downstream analyses.

Filtered reads were *de novo* assembled using SPAdes in careful mode to minimize mismatches and short indels. Finally, filtered reads were mapped back to the assembled genomes, and the assemblies were polished using Pilon to correct residual errors.

### Immunofluorescence

Cells were infected with a MOI of 0.5. Twenty-four hours after infection, cells were washed with phosphate-buffered saline (PBS) and then fixed with a cold methanol/acetone solution (1:1) for 15 min at −20°C. After fixation, cells were washed twice with eBioscience permeabilization buffer (Invitrogen) and once with permeabilization buffer supplemented with 2% goat serum (PB-GS). Coverslips were incubated with a rabbit antibody against adenoviral structural proteins (1:10,000) and a mouse anti-FLAG M2 antibody (1:1,000) (Sigma-Aldrich) in PB-GS for 1 h at 4°C. After two washes with PB-GS, coverslips were incubated with goat anti-rabbit antibody coupled with Alexa 633 (1:1,000) (Invitrogen), goat anti-mouse antibody coupled with Alexa 568 (1:1,000) (Invitrogen), and DAPI (1:10,000) (Thermo Fisher Scientific) in PB-GS for 1 h at 4°C. Coverslips were washed twice with PB-GS and mounted using Fluoromount-G (Invitrogen). The slides were imaged using a Leica TCS SP5 microscope.

### Viral replication kinetics

Kinetics of infection were compared by flow cytometry. Cells were infected with a MOI of 0.2 and harvested at four time points (24, 30, 36, and 48 h post-infection). At the corresponding time point, cells were trypsinized for 5 min. Once cells were fully detached, trypsin was neutralized using 10% FCS DMEM, and the cell suspension was collected. Cells were centrifuged at 300 × *g* for 5 min and washed twice with PBS. Cells were fixed using 4% PFA for 15 min at 4°C, washed twice with PBS, permeabilized with PBS Triton 0.1% for 20 min at RT, and washed twice with PBS. Cells were incubated for 1 h at 4°C with anti-adenovirus antibody (MAB 8051, Merck Millipore) used at a dilution of 1:200 in PBS supplemented with 2% of goat serum (PBS-GS) (Gibco). After two washes with PBS-GS, cells were incubated for 1 h at 4°C with an anti-mouse antibody coupled with Alexa 633 (Invitrogen) at a dilution of 1:1,000 in PBS-GS. Cells were washed twice with PBS-GS and finally resuspended in PBS. The percentage of HAdV-infected cells was quantified using a CytoFLEX S V4-B2-Y4-R3 Flow Cytometer (Beckman Coulter) and analyzed using FlowJo software version 10.10.0 (BD Biosciences).

## RESULTS

### Overview of AdamiForge

We partitioned the HAdV-A12 genome into seven blocks ranging from 3.3 to 7.3 kb (Fig. 1A; Table S2). When designing the blocks, we sought to preserve the organization of transcription units and avoid splitting genes across different blocks, thus facilitating subsequent genetic modifications (Fig. 1A). The only exception is the terminal protein (TP) whose coding sequence is split between block 2 and block 3. The first step of the AdamiForge workflow is to release the different blocks from their vector (Fig. 1B). To achieve this, a ClaI restriction site has been added at both ends of each block (the ClaI restriction site, ATCGAT, is not present in the HAdV-A12 genome). Importantly, ClaI is blocked by overlapping dam methylation. If future users wish to modify the plasmids, they must ensure that no dam methylation site (GATC) overlaps the ClaI restriction site (ATCGAT). Therefore, it is imperative to avoid introducing a G or C flanking the ClaI site. Of note, the BstBI restriction enzyme originally used in the AdenoBuilder system could not be used here, as BstBI cuts the A12 genome at several places. Each of the seven blocks shares a short overlapping region (19 bp–30 bp) with their neighboring block to allow full-length genome reassembly. The reconstructed genome can be directly transfected into permissive cells to enable the production of new viral particles (Fig. 1B).

**Fig 2:**
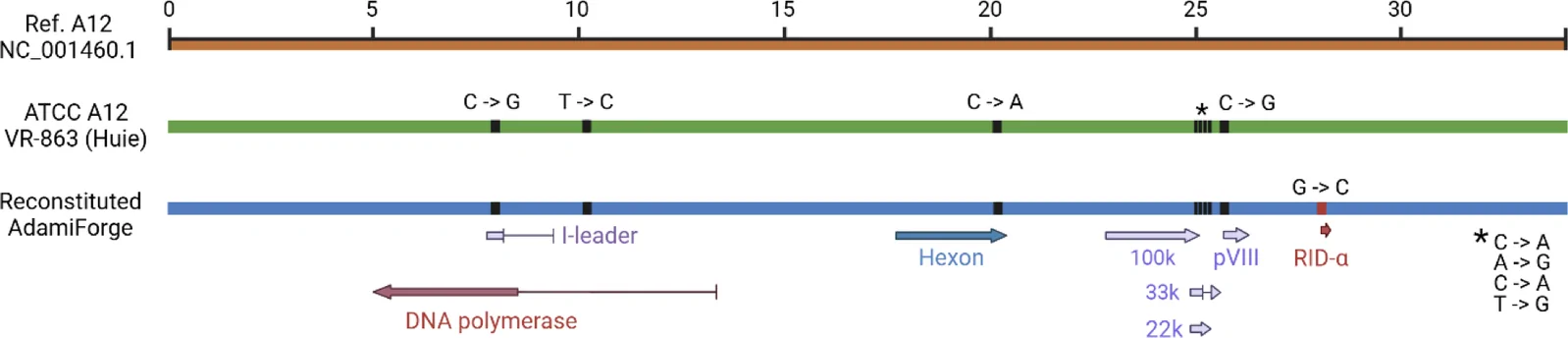
The AdamiForge WT viral genome differs at nine positions from the commonly used reference sequence NC_001460.1. Sequence comparison of the HAdV-A12 reference genome (NC_001460.1), the ATCC Huie isolate (VR-863), and the reconstituted AdamiForge genome. The Huie isolate differs from the reference sequence by eight nucleotide substitutions. The AdamiForge-derived genome contains the same substitutions and harbors one additional synonymous nucleotide change introduced during cloning. Asterisk indicates four closely spaced mutations, listed from the 5′ to 3′ end of the sense genome.

The AdamiForge tool is based on the Huie strain (ATCC catalog VR-863). The reconstituted wild-type AdamiForge virus (AF WT) differs from the Huie strain by a G-to-C mutation at position 28,212 in the E3 RID-α gene (Fig. 2; Table 1). This mutation generates a GTG-to-GTC synonymous substitution and is therefore not expected to impact the fitness of the virus. When compared to the commonly used reference sequence NC_001460.1, AF WT contains eight additional nucleotide substitutions (Fig. 2; Table 1). These differences lead to the changes in 12 codons, 4 of which are synonymous. Plasmids and AF WT genomic sequences are available in the supporting information (Table S3).

**TABLE 1:** Nucleotide differences between the AdamiForge WT genome and the commonly used reference sequence NC_001460.1^*a*^

Position	Nucleotide		Codon			Amino acid	
	NC_001460.1	AdamiForge	Gene	NC_001460.1	AdamiForge	NC_001460.1	AdamiForge
8043	C	G	I-Leader :	**C**CT	GCT	Proline	Alanine
			Pol	AG**G**	AGC	Arginine	Serine
10252	T	C	Non-coding				
20197	C	A	Hexon	**C**AA	AAA	Glutamine	Lysine
24972	C	A	100k	**C**AA	AAA	Glutamine	Lysine
			33k	GC**C**	GCA	Alanine	Alanine
			22k	GC**C**	GCA	Alanine	Alanine
25037	A	G	100k :	CA**A**	CAG	Glutamine	Glutamine
			33k	A**A**C	AGC	Asparagine	Serine
			22k	A**A**C	AGC	Asparagine	Serine
25129	C	A	22k	**C**AA	AAA	Glutamine	Lysine
25152	T	G	22k	TT**T**	TTG	Phenylala.	Leucine
25656	C	G	pVIII	CC**C**	CCG	Proline	Proline
28212*	G	C	E3 RID-a	GT**G**	GTC	Valine	Valine

^*a*^ The sequences differ at nine positions, affecting 13 coding sequences. Synonymous mutations are indicated in gray shading. The G-to-C mutation at position 28212, marked by an asterisk, was absent from the original Huie strain. The other mutations were already present in the Huie strain. The mutated nucleotides are shown in bold.

### Block release, genome reassembly, and production of viral particles

**Fig 3:**
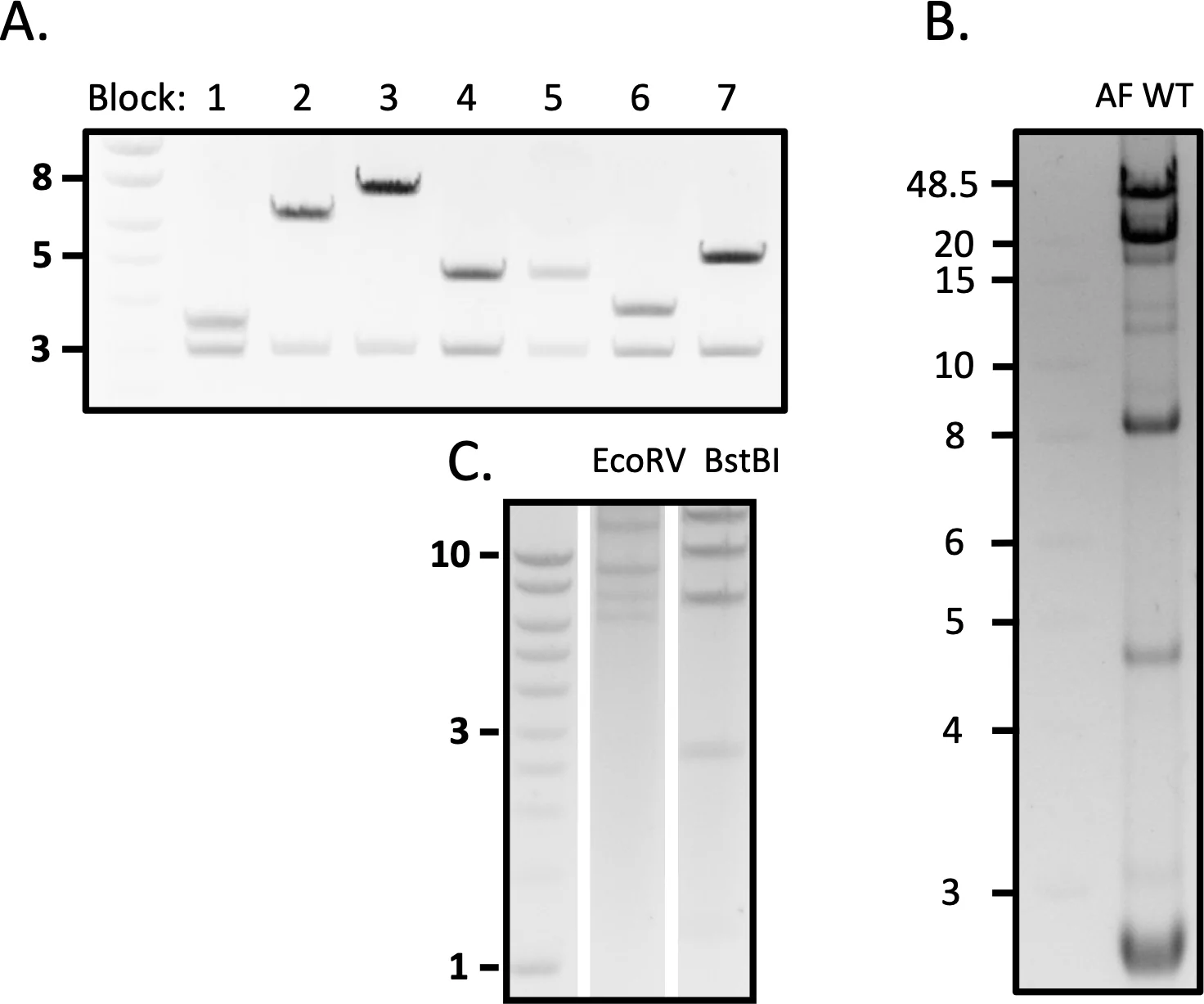
AdamiForge workflow allows the assembly of full-length HAdV-A12 genome. (**A**) Individual vector plasmids were digested with ClaI to release the genomic blocks. A 2,974 bp fragment corresponding to the vector backbone was observed in all conditions. A second band was observed ranging in size from 3,316 to 7,358 bp corresponding to the genomic block. (**B**) Purified genomic blocks were pooled and assembled by Gibson assembly. A prominent DNA band corresponding to the expected size (34,125 bp) was observed. Additional smaller bands likely represent assembly by-products. (**C**) Reconstituted viral genomes were digested with EcoRV or BstBI to verify the restriction profiles. The observed fragment sizes correspond to the expected digestion patterns (EcoRV: 229 [not visible], 5,928, 6,905, 8,461, and 12,602 bp; BstBI: 1,097, 2,553, 6,669, 9,698, and 14,108 bp).

The first step of the protocol consists of the digestion of the different plasmids by ClaI to release the blocks. The completion of this step can be verified on agarose gel. The different plasmids release two fragments: the pJET1.2 backbone (expected at 2,974 bp) and the corresponding block (ranging from 3,316 to 7,358 bp) (Fig. 3A; Table S2). After ClaI digestion, the DNA is precipitated to remove the digestion mix prior to DNA recombination. Due to the nature of the Gibson assembly and the number of fragments that need to be ligated together, the complete recombination process is imperfect (the more fragments present, the lower the probability of obtaining a correctly assembled full-length genome). It is expected to have numerous truncated versions of the assembly. Nevertheless, we observed a relatively prominent band around the expected size (34,125 bp), in addition to all the subproducts of the DNA recombination (Fig. 3B). The recombination product is transfected into HEK-293T cells to allow the generation of new HAdV-A12 particles. The main difficulty of the system remains in this last step, as HAdV-A12 can be harder to propagate than HAdV-C5. After recovery of the first virions produced in HEK-293T cells, successive rounds of amplification in A549 cells deficient in IFN signaling (like A549 PIV5-V) will be necessary to achieve a high-titer production. Finally, the integrity of the reconstituted virus can be quickly checked by restriction profile. Indeed, restriction of DNA from newly produced virions with EcoRV yielded five fragments of the following sizes: 229, 5,928, 6,905, 8,461, and 12,602 (the 229 bp fragment cannot be seen). Similarly, digestion with BstBI produced five fragments of the following sizes: 1,097, 2,553, 6,669, 9,698, and 14,108 bp (although the 1,097 bp fragment can be difficult to see) (Fig. 3C).

### Creation of modified HAdV-A12 using the AdamiForge system

We successfully produced wild-type HAdV-12 using the AdamiForge platform. However, the true value of this kind of tool lies in their ability to engineer viruses. As proof of concept, we developed two HAdV-A12 recombinants expressing modified DBP. We chose to fuse eGFP to DBP to generate a fluorescently labeled HAdV-A12 (named AF eGFP-DBP thereafter), enabling applications such as live imaging. In parallel, we introduced a 3xFLAG-tag into a second DBP construct (named AF FLAG-DBP) to notably overcome limitations in antibody availability and to facilitate analyses such as Western blot or co-immunoprecipitation. In both cases, the modifications were introduced at the N-terminal end of the DBP protein. In practice, we modified the plasmid corresponding to the block 5 to insert the respective tag (eGFP or 3xFLAG) into the DBP coding sequence. The workflow remains the same, except that the wild-type block 5 is replaced by its modified equivalent. ClaI digestion of this modified block releases the engineered genomic fragment. Wild-type block 5 fragment is expected at 4,419 bp, eGFP-DBP block 5 at 5,149 bp, and the 3xFLAG-DBP block 5 at 4,498 bp (Fig. S1).

**Fig 4:**
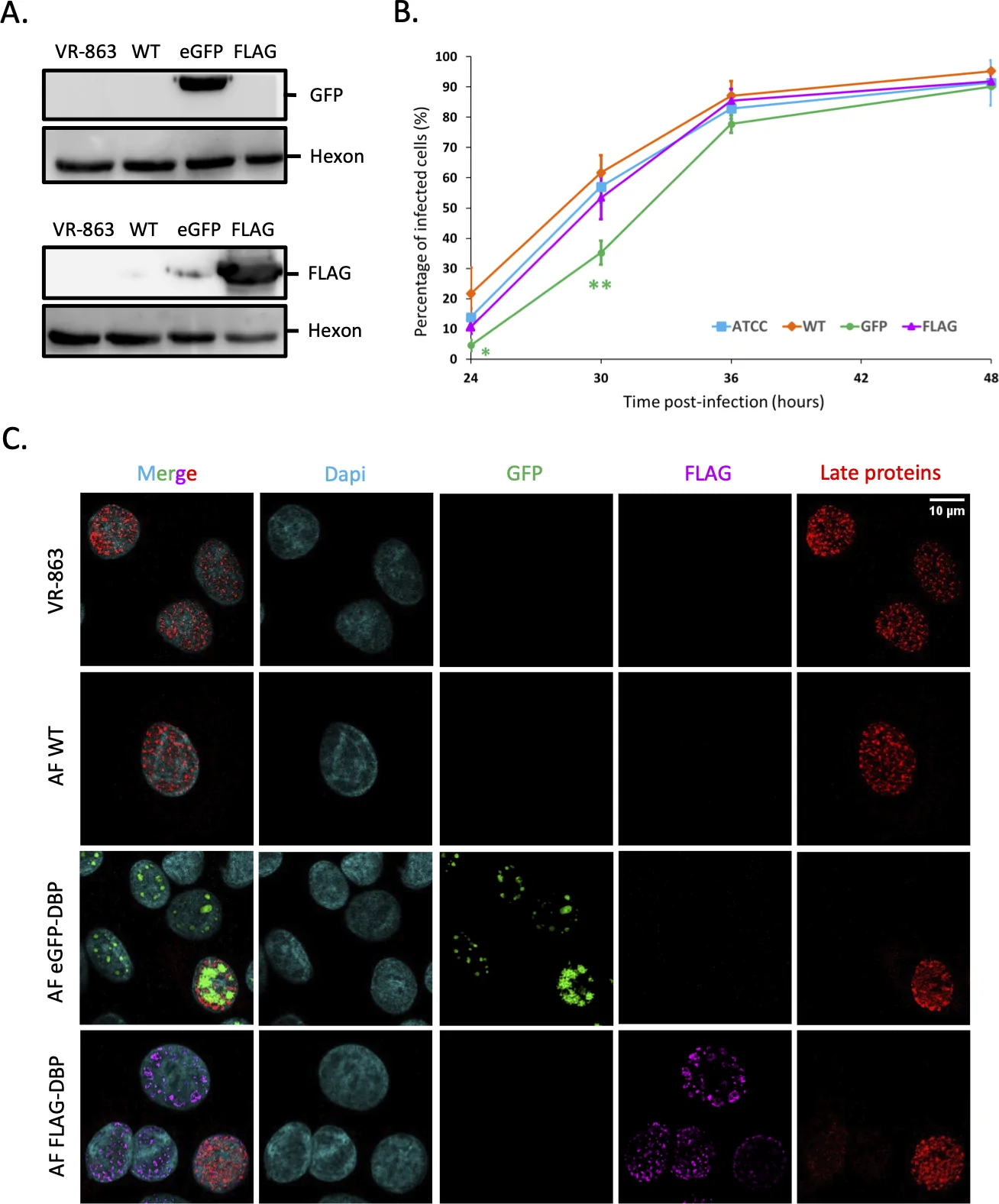
AdamiForge-based HAdV-A12 recombinants enable detection of the viral DNA-binding protein in the absence of specific antibodies. (**A**) A549 cells were infected with ATCC isolate VR-863, AF WT, AF eGFP-DBP, or AF FLAG-DBP. Modified DBP proteins were detected using commercially available antibodies. (**B**) Viral replication kinetics were monitored by flow cytometry. Percentage of infected cells was monitored at four time points post-infection. *P*-values were calculated by *t*-tests. Only significant differences are shown. (**C**) Infected cells were imaged by confocal microscopy. eGFP-DBP was visualized directly, and FLAG-DBP was detected using commercially available anti-FLAG antibody. *P*-values were calculated by *t*-tests. Only significant differences are shown ( *P < 0.05; **P < 0.01).

Human lung carcinoma cells A549 PIV5-V were infected respectively by ATCC isolate VR-863, AF WT, AF eGFP-DBP, or AF FLAG-DBP. As mentioned above, the modifications address the absence of antibodies against HAdV-A12 DBP. Using commercially available reagents, we successfully detected the modified HAdV-A12 DBP protein with anti-FLAG and anti-GFP antibodies (Fig. 4A). The replication kinetics of the recombinant viruses were assessed to evaluate potential effects of the introduced tags on viral fitness. The percentage of infected cells was measured by flow cytometry at different time points post-infection (Fig. 4B). The results show that AF WT and the AF FLAG-DBP display comparable replication kinetics to those of the ATCC isolate VR-863. However, AF eGFP-DBP exhibits slower kinetics at early time points but ultimately reaches a similar percentage of infected cells as the other viruses after 48 h of infection (Fig. 4B). This delayed replication for AF eGFP-DBP shows that not all modifications are functionally neutral. Fusion of the 27 kDa eGFP protein to DBP reduces replication kinetics, consistent with previous descriptions for the HAdV-C5 strain (15). Finally, the cells infected by the four different viruses were also imaged by confocal microscopy. The eGFP-tagged DBP allows visualization of DBP without the need for antibodies, while the FLAG-tagged version provides an accessible epitope for an antibody-based labeling in the absence of a specific HAdV-A12 DBP antibody (Fig. 4C). The cells observed represent different stages of the infection cycle. Some cells are in an early stage of the viral cycle, where DBP is present as small nuclear foci and the viral late proteins cannot be detected. In constrast, other cells are more advanced in the viral cycle as shown by the strong late proteins’ labeling, where DBP accumulates, takes a larger part of the nucleus, and eventually concentrates at the nuclear periphery.

## DISCUSSION

Although the generation of modified adenoviruses has been possible for decades, most approaches required cloning the viral genome into a bacmid and using homologous recombination in bacteria to replace a specific region with the desired sequence (32–35). Unfortunately, these approaches remain time-consuming and require rare expertise. Alternative approaches based on the partitioning of the viral genome into several high-copy plasmids have been developed. Editing of individual plasmids relies on routine laboratory techniques, while Gibson assembly enables straightforward reconstruction of the complete viral genome (13, 14). Building on this latter approach, we have developed a new tool to create HAdV-A12 mutants. The partitioning of the viral genome into seven blocks allows individual viral genes to be easily manipulated in isolation and then recombined with other mutations or transgenes. Full-length genome recombination is achieved within one day, and recombinant viral stock can be expected within two weeks. Recently, Vergnes and colleagues developed an elegant protocol for manipulating the adenoviral genome, called AdVICE (36). Thus, the viral genome is cloned into a plasmid, and its modification is achieved through targeted CRISPR/Cas9-mediated cleavage and Gibson assembly. In theory, engineered HAdV-A12 could be generated with either AdVICE or AdamiForge within comparable timeframes and with similar technical expertise.

In addition to the reconstitution of wild-type HAdV-A12, we engineered the virus to express modified DBP in two different ways: a FLAG-tagged version and a fluorescently labelled version generated by fusion with eGFP. The AF eGFP-DBP allows the visualization of viral replication centers in infected cells without using standard immunostaining methods. The morphology of DBP evolves during the infection, appearing as small nuclear foci in the early stages, then developing into larger structures, and finally concentrating at the nuclear periphery in the later stages. Because AF eGFP-DBP displays a slight delay in replication, the use of AF 3xFLAG-DBP would be more suitable for studies requiring minimally perturbed viral behavior.

Although it is not within the scope of this article, the AdamiForge tool could be instrumental in extending our knowledge of HAdV-A12 and for the design of A12-based therapeutics, viruses, and vectors. As opposed to its congeneric counterpart HAdV-C5, which has been extensively studied, HAdV-A12 has been relatively overlooked. Consequently, the panel of available tools, such as reverse genetics systems or antibodies, remains very limited. Nevertheless, HAdV-A12 is not without significance. Adenoviruses species A, and in particular HAdV-A12, were the first human viruses shown to promote tumors in non-human models (25–28). In contrast, HAdV-C5 displays a much lower oncogenic potential in animal models. These distinct cell-transforming abilities have been notably linked to differences in the E1A and E1B-55k proteins, but a comprehensive understanding of A12 oncogenic properties has been limited by the absence of a reverse genetics tool for HAdV-A12 (37). It is precisely because of their oncogenic properties in rodents that species A adenoviruses have not been considered suitable candidates to serve as the basis for oncolytic viruses, vaccines, or gene therapy vectors, even though no link has ever been observed between infection with species A and cancer in humans. Interestingly, a C5-based replicative vector expressing the A12 E1A protein has been shown to kill prostate cancer cells (38, 39). Thus, the availability of a reverse genetics tool for HAdV-A12 could also spur the testing of A12-based oncolytic vectors. The use of first-generation adenoviral vectors for vaccine application has been successfully demonstrated during the SARS-CoV-2 pandemic. Albeit the AstraZeneca COVID-19 vaccine contributed to save countless lives, its use has been associated with a rare but severe autoimmune reaction characterized by a combination of thrombotic events and thrombocytopenia (40). Mechanistically, PF4 protein (Platelet Factor 4) binds to the vaccine capsid, setting off the production of anti-PF4 antibodies (41). These autoantibodies will in turn trigger vaccine‐induced immune thrombotic thrombocytopenia (VITT). Since then, the scientific community has devoted considerable effort to addressing this issue, mainly by selecting adenoviral capsids that do not bind PF4. Interestingly, the HAdV-A12 capsid does not seem to bind PF4, suggesting that an A12-based vector should not elicit VITT (42). Thus, the AdamiForge tool could be used to design A12-based vaccines and evaluate their efficacy and safety profile. Although they belong to the same genus, adenovirus species display distinct characteristics. While the genome organization is broadly conserved, function and/or subcellular localization of certain proteins substantially differs, and certain regions, such as E3, exhibit species-specific organization (43). For example, species A does not encode the E3 gp19k protein responsible, in HAdV-C5, for MHCI downregulation (44). Although p53 inactivation is conserved among adenoviruses, the mechanisms differ between species. For instance, the E4orf6 - E1B-55k complex from HAdV-C5 mediates p53 degradation via a Cul5-based ubiquitin ligase complex, whereas HAdV-A12 recruits a Cul2-based complex (45). Another example concerns the antagonism of the innate immune effector APOBEC3B, a cytidine deaminase that exerts selective pressure and restricts adenovirus replication by introducing mutations into the viral genome (46). While HAdV-C2 promotes a reduction of APOBEC3B protein levels, HAdV-A12 does not. Instead, HAdV-A12 lowers the enzymatic activity of APOBEC3B (47, 48). To date, the molecular mechanism underlying the antagonization of APOBEC3B remains unknown. Thus, the aforementioned examples highlight the importance of developing research tools for HAdV-A12 (as well as for the many other understudied adenoviruses).

In summary, we have adapted the modular AdenoBuilder system to the HAdV-A12 strain, creating a new reverse genetics tool for A12: AdamiForge. This tool enables the generation of all types of mutant viruses, including insertion, deletion, or modification of specific genes, all of which can be combined within a single final construct. Such an instrument can notably compensate for the lack of antibodies specific to HAdV-A12. AdamiForge fills a gap in the adenovirus field. It will enable a more comprehensive understanding of HAdV-A12 physiology and support the field through comparative studies with related viruses.

## Supplementary Material

Reviewer comments

## SUPPLEMENTAL MATERIAL

Supplemental Material spectrum.00696-26-s0001.docx Tables S1 and S3 captions, Table S2, and Figure S1.

Table S1 spectrum.00696-26-s0002.xlsx Primer list and PCR thermal protocols.

Table S3 spectrum.00696-26-s0003.docx Plasmids and whole viral genome sequences.

OPEN PEER REVIEW reviewer-comments.pdf An accounting of the reviewer comments and feedback.

ASM does not own the copyrights to Supplemental Material that may be linked to, or accessed through, an article. The authors have granted ASM a non-exclusive, world-wide license to publish the Supplemental Material files. Please contact the corresponding author directly for reuse.

## References

[1] Lynch JP, Kajon AE. 2016. Adenovirus: epidemiology, global spread of novel serotypes, and advances in treatment and prevention. Semin Respir Crit Care Med 37:586–602. doi:10.1055/s-0036-1584923 27486739 PMC7171713

[2] Binder AM, Biggs HM, Haynes AK, Chommanard C, Lu X, Erdman DD, Watson JT, Gerber SI. 2017. Human Adenovirus Surveillance - United States, 2003-2016. MMWR Morb Mortal Wkly Rep 66:1039–1042. doi:10.15585/mmwr.mm6639a2 28981484 PMC5720882

[3] Lion T. 2014. Adenovirus infections in immunocompetent and immunocompromised patients. Clin Microbiol Rev 27:441–462. doi:10.1128/CMR.00116-13 24982316 PMC4135893

[4] Garnett CT, Erdman D, Xu W, Gooding LR. 2002. Prevalence and quantitation of species C adenovirus DNA in human mucosal lymphocytes. J Virol 76:10608–10616. doi:10.1128/jvi.76.21.10608-10616.2002 12368303 PMC136639

[5] Lion T. 2019. Adenovirus persistence, reactivation, and clinical management. FEBS Lett 593:3571–3582. doi:10.1002/1873-3468.13576 31411731

[6] Watanabe M, Nishikawaji Y, Kawakami H, Kosai K-I. 2021. Adenovirus biology, recombinant adenovirus, and adenovirus usage in gene therapy. Viruses 13:2502. doi:10.3390/v13122502 34960772 PMC8706629

[7] Douglas JT. 2007. Adenoviral vectors for gene therapy. Mol Biotechnol 36:71–80. doi:10.1007/s12033-007-0021-5 17827541

[8] Bulcha JT, Wang Y, Ma H, Tai PWL, Gao G. 2021. Viral vector platforms within the gene therapy landscape. Signal Transduct Target Ther 6:53. doi:10.1038/s41392-021-00487-6 33558455 PMC7868676

[9] Zhao Z, Anselmo AC, Mitragotri S. 2022. Viral vector-based gene therapies in the clinic. Bioeng Transl Med 7:e10258. doi:10.1002/btm2.10258 35079633 PMC8780015

[10] Mercado NB, Zahn R, Wegmann F, Loos C, Chandrashekar A, Yu J, Liu J, Peter L, McMahan K, Tostanoski LH, et al.. 2020. Single-shot Ad26 vaccine protects against SARS-CoV-2 in rhesus macaques. Nature 586:583–588. doi:10.1038/s41586-020-2607-z 32731257 PMC7581548

[11] Barrett JR, Belij-Rammerstorfer S, Dold C, Ewer KJ, Folegatti PM, Gilbride C, Halkerston R, Hill J, Jenkin D, Stockdale L, et al.. 2021. Phase 1/2 trial of SARS-CoV-2 vaccine ChAdOx1 nCoV-19 with a booster dose induces multifunctional antibody responses. Nat Med 27:279–288. doi:10.1038/s41591-020-01179-4 33335322

[12] Hong L, et al.. 2025. The seroprevalence of adenoviruses since 20001. Emerg. Microbes Infect 14:2475831. doi:10.1080/22221751.2025.2475831 PMC11915735 40035700

[13] Miciak JJ, Hirshberg J, Bunz F. 2018. Seamless assembly of recombinant adenoviral genomes from high-copy plasmids. PLoS One 13:e0199563. doi:10.1371/journal.pone.0199563 29949649 PMC6021080

[14] Jang Y, Bunz F. 2022. AdenoBuilder: a platform for the modular assembly of recombinant adenoviruses. STAR Protoc 3:101123. doi:10.1016/j.xpro.2022.101123 35098167 PMC8783202

[15] King CR, Dodge MJ, MacNeil KM, Tessier TM, Mymryk JS, Mehle A. 2024. Expanding the adenovirus toolbox: reporter viruses for studying the dynamics of human adenovirus replication. J Virol 98:e0020724. doi:10.1128/jvi.00207-24 38639487 PMC11092356

[16] O’Brien CM, Serra L, Patterson MR, Acosta RW, Yu A, Claiborne DT, Price AM. 2025. Replication-competent adenovirus reporters utilizing endogenous viral expression architecture. J Virol 99:e0114625. doi:10.1128/jvi.01146-25 40928252 PMC12548470

[17] C HG, E EW. 1964. Adenovirus type 12 infection. Clinical and laboratory studieS. JAMA 188:1086–1088. doi:10.1001/jama.1964.03060380054019 14151175

[18] D’ambrosio E, Grosso ND, Chicca A, Midulla M. 1982. Neutralizing antibodies against 33 human adenoviruses in normal children in Rome. J Hyg 89:155–161. doi:10.1017/S0022172400070650 7096999 PMC2134173

[19] Vogels R, Zuijdgeest D, van Rijnsoever R, Hartkoorn E, Damen I, de Béthune M-P, Kostense S, Penders G, Helmus N, Koudstaal W, Cecchini M, Wetterwald A, Sprangers M, Lemckert A, Ophorst O, Koel B, van Meerendonk M, Quax P, Panitti L, Grimbergen J, Bout A, Goudsmit J, Havenga M. 2003. Replication-deficient human adenovirus type 35 vectors for gene transfer and vaccination: efficient human cell infection and bypass of preexisting adenovirus immunity. J Virol 77:8263–8271. doi:10.1128/jvi.77.15.8263-8271.2003 12857895 PMC165227

[20] Wang X, Aurich K, Zhang W, Ehrhardt A, Greinacher A, Bayer W. 2024. Longitudinal analysis of binding antibody levels against 39 human adenovirus types in sera from 60 regular blood donors from Greifswald, Germany, over 5 years from 2018 to 2022. Viruses 16:1747. doi:10.3390/v16111747 39599861 PMC11598854

[21] Kibrick S, Melendez L, Enders JF. 1957. Clinical associations of enteric viruses with particular reference to agents exhibiting properties of the ECHO group. Ann N Y Acad Sci 67:311–325. doi:10.1111/j.1749-6632.1957.tb46055.x 13411969

[22] Davison AJ, Benkő M, Harrach B. 2003. Genetic content and evolution of adenoviruses. J Gen Virol 84:2895–2908. doi:10.1099/vir.0.19497-0 14573794

[23] Roelvink PW, Lizonova A, Lee JG, Li Y, Bergelson JM, Finberg RW, Brough DE, Kovesdi I, Wickham TJ. 1998. The coxsackievirus-adenovirus receptor protein can function as a cellular attachment protein for adenovirus serotypes from subgroups A, C, D, E, and F. J Virol 72:7909–7915. doi:10.1128/JVI.72.10.7909-7915.1998 9733828 PMC110119

[24] Bewley MC, Springer K, Zhang Y-B, Freimuth P, Flanagan JM. 1999. Structural analysis of the mechanism of adenovirus binding to its human cellular receptor, CAR. Science 286:1579–1583. doi:10.1126/science.286.5444.1579 10567268

[25] Yabe Y, Trentin JJ, Taylor G. 1962. Cancer induction in hamsters by human type 12 adenovirus. Effect of age and of virus dose. Exp Biol Med (Maywood) 111:343–344. doi:10.3181/00379727-111-27786 14002172

[26] Rabson AS, Kirschstein RL, Paul FJ. 1964. Tumors produced by adenovirus 12 in mastomys and mice. JNCI J Natl Cancer Inst 32:77–87. doi: 10.1093/jnci/32.1.77 14114974

[27] Utsumi KR, Kitamura I, Trentin JJ. 1965. Karyologic studies of normal cells and of adenovirus-type-12-induced tumor cells of the Syrian hamster. J Natl Cancer Inst 35:759–769. doi:10.1093/jnci/35.5.759 5849591

[28] Mukai N, Kalter SS, Cummins LB, Matthews VA, Nishida T, Nakajima T. 1980. Retinal tumor induced in the baboon by human adenovirus 12. Science 210:1023–1025. doi:10.1126/science.7434012 7434012

[29] Williams JF. 2004. E1A-based determinants of oncogenicity in human adenovirus groups A and C, p 245–288. *In* Doerfler W, Böhm P (ed), Adenoviruses: model and vectors in virus-host interactions: immune system, oncogenesis, gene therapy. Springer, Berlin, Heidelberg. 10.1007/978-3-662-05599-1_8 14674604

[30] Endter C, Dobner T. 2004. Cell transformation by human adenoviruses, p 163–214. *In* Doerfler W, Böhm P (ed), Adenoviruses: model and vectors in virus-host interactions: immune system, oncogenesis, gene therapy. Springer, Berlin, Heidelberg.

[31] Jelinek T, Graham FL. 1992. Recombinant human adenoviruses containing hybrid adenovirus type 5 (Ad5)/Ad12 E1A genes: characterization of hybrid E1A proteins and analysis of transforming activity and host range. J Virol 66:4117–4125. doi:10.1128/JVI.66.7.4117-4125.1992 1534849 PMC241214

[32] Chartier C, Degryse E, Gantzer M, Dieterle A, Pavirani A, Mehtali M. 1996. Efficient generation of recombinant adenovirus vectors by homologous recombination in *Escherichia coli**.* J Virol 70:4805–4810. doi:10.1128/JVI.70.7.4805-4810.1996 8676512 PMC190422

[33] He TC, Zhou S, da Costa LT, Yu J, Kinzler KW, Vogelstein B. 1998. A simplified system for generating recombinant adenoviruses. Proc Natl Acad Sci USA 95:2509–2514. doi:10.1073/pnas.95.5.2509 9482916 PMC19394

[34] Ruzsics Z, Lemnitzer F, Thirion C. 2014. Engineering adenovirus genome by bacterial artificial chromosome (BAC) technology. Methods Mol Biol 1089:143–158. doi:10.1007/978-1-62703-679-5_11 24132484

[35] Mück-Häusl M, Solanki M, Zhang W, Ruzsics Z, Ehrhardt A. 2015. Ad 2.0: a novel recombineering platform for high-throughput generation of tailored adenoviruses. Nucleic Acids Res 43:e50. doi:10.1093/nar/gkv031 25609697 PMC4417142

[36] Vergnes J-B, Roger B, Iggo R, Wodrich H. 2025. Advanced viral genome *in vitro* Cas9 editing (AdVICE): an overnight method for traceless and limitless manipulation of adenoviral and vector genomes with large transgenes. J Virol 99:e02265-24. doi:10.1128/jvi.02265-24 40396759 PMC12172474

[37] Bertzbach LD, Ip W-H, von Stromberg K, Dobner T, Grand RJ. 2024. A comparative review of adenovirus A12 and C5 oncogenes. Curr Opin Virol 67:101413. doi:10.1016/j.coviro.2024.101413 38865835

[38] Wang Y, Li D, Luo J, Tian G, Zhao LY, Liao D. 2016. Intrinsic cellular signaling mechanisms determine the sensitivity of cancer cells to virus-induced apoptosis. Sci Rep 6:37213. doi:10.1038/srep37213 27849011 PMC5111159

[39] Li D, Tian G, Wang J, Zhao LY, Co O, Underill ZC, Mymryk JS, Claessens F, Dehm SM, Daaka Y, Liao D. 2018. Inhibition of androgen receptor transactivation function by adenovirus type 12 E1A undermines prostate cancer cell survival. Prostate 78:1140–1156. doi:10.1002/pros.23689 30009471 PMC6424568

[40] Greinacher A, Thiele T, Warkentin TE, Weisser K, Kyrle PA, Eichinger S. 2021. Thrombotic thrombocytopenia after ChAdOx1 nCov-19 vaccination. N Engl J Med 384:2092–2101. doi:10.1056/NEJMoa2104840 33835769 PMC8095372

[41] Baker AT, Boyd RJ, Sarkar D, Teijeira-Crespo A, Chan CK, Bates E, Waraich K, Vant J, Wilson E, Truong CD, Lipka-Lloyd M, Fromme P, Vermaas J, Williams D, Machiesky L, Heurich M, Nagalo BM, Coughlan L, Umlauf S, Chiu P-L, Rizkallah PJ, Cohen TS, Parker AL, Singharoy A, Borad MJ. 2021. ChAdOx1 interacts with CAR and PF4 with implications for thrombosis with thrombocytopenia syndrome. Sci Adv 7:eabl8213. doi:10.1126/sciadv.abl8213 34851659 PMC8635433

[42] Sallard E, Pembaur D, Ciancaglini M, Manov-Bouard L, Weklak D, Hamdan F, Chan CK, Jönsson F, Chabot E, Musielak C, et al.. 2026. Novel adenovirus vaccine vectors lacking thrombosis-associated interactions with platelet factor 4. iScience 29:114329. doi:10.1016/j.isci.2025.114329 41509905 PMC12775996

[43] Robinson CM, Seto D, Jones MS, Dyer DW, Chodosh J. 2011. Molecular evolution of human species D adenoviruses. Infect Genet Evol 11:1208–1217. doi:10.1016/j.meegid.2011.04.031 21570490 PMC3139803

[44] Sester M, Ruszics Z, Mackley E, Burgert H-G. 2013. The transmembrane domain of the adenovirus E3/19K protein acts as an endoplasmic reticulum retention signal and contributes to intracellular sequestration of major histocompatibility complex class I molecules. J Virol 87:6104–6117. doi:10.1128/JVI.03391-12 23514889 PMC3648096

[45] Cheng CY, Gilson T, Dallaire F, Ketner G, Branton PE, Blanchette P. 2011. The E4orf6/E1B55K E3 ubiquitin ligase complexes of human adenoviruses exhibit heterogeneity in composition and substrate specificity. J Virol 85:765–775. doi:10.1128/JVI.01890-10 21068234 PMC3020000

[46] Poulain F, Lejeune N, Willemart K, Gillet NA. 2020. Footprint of the host restriction factors APOBEC3 on the genome of human viruses. PLoS Pathog 16:e1008718. doi:10.1371/journal.ppat.1008718 32797103 PMC7449416

[47] Lejeune N, Mathieu S, Decloux A, Poulain F, Blockx Z, Raymond KA, Willemart K, Vartanian J-P, Suspène R, Gillet NA. 2023. The APOBEC3B cytidine deaminase is an adenovirus restriction factor. PLoS Pathog 19:e1011156. doi:10.1371/journal.ppat.1011156 36745676 PMC9934312

[48] Lejeune N, Poulain F, Willemart K, Blockx Z, Mathieu S, Gillet NA. 2021. Infection of bronchial epithelial cells by the human adenoviruses A12, B3, and C2 differently regulates the innate antiviral effector APOBEC3B. J Virol 95:e0241320. doi:10.1128/JVI.02413-20 33853956 PMC8315923

