# Supplementary material for: AdamiForge: a modular reverse genetics tool for human adenovirus type 12 (HAdV-A12)
Source: Microbiol Spectr. 2026 May 21;14(7):e00696-26. doi: 10.1128/spectrum.00696-26 (PMC13340337; doi:10.1128/spectrum.00696-26)

**Supplemental Material - AdamiForge**

**Table S1. Primer list and PCR thermal protocols**

Table S1.xlsx

**Table S2. Size (bp) of each cloned plasmid block and corresponding gene list.**

**Table S3. AdamiForge genome sequence**

Table S3.docx

**Figure S1. Release of modified block 5 following ClaI digestion.** ClaI digestion releases a 2,974 bp fragment corresponding to the vector backbone and a second fragment corresponding to the genomic segment (WT: 4419, eGFP: 5149, FLAG: 4498 bp).


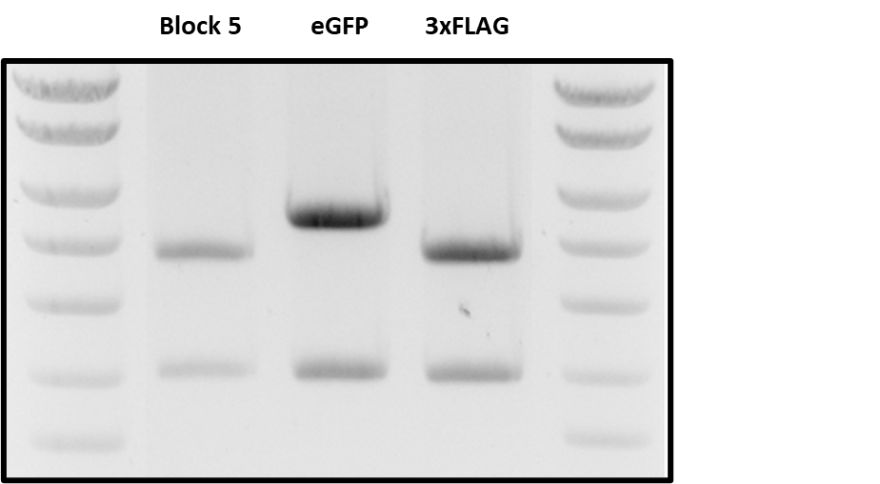

Supplement: Supplemental Material — Tables S1 and S3 captions, Table S2, and Figure S1. [file spectrum.00696-26-s0001.docx]
