# Supplementary material for: AdamiForge: a modular reverse genetics tool for human adenovirus type 12 (HAdV-A12)
Source: Microbiol Spectr. 2026 May 21;14(7):e00696-26. doi: 10.1128/spectrum.00696-26 (PMC13340337; doi:10.1128/spectrum.00696-26)
Supplement: Table S3 — Plasmids and whole viral genome sequences. [file spectrum.00696-26-s0003.docx]

>AdamiForge_sequence

CCTATCTAATAATATACCTTATACTGGACTAGTGCCAATATTAAAATGAAGTGGGCGTAGTGTGTAATTTGATTGGGTGGAGGTGTGGCTTTGGCGTGCTTGTAAGTTTGGGCGGATGAGGAAGTGGGGCGCGGCGTGGGAGCCGGGCGCGCCGGATGTGACGTTTTAGACGCCATTTTACACGGAAATGATGTTTTTTGGGCGTTGTTTGTGCAAATTTTGTGTTTTAGGCGCGAAAACTGAAATGCGGAAGTGAAAATTGATGACGGCAATTTTATTATAGGCGCGGAATATTTACCGAGGGCAGAGTGAACTCTGAGCCTCTACGTGTGGGTTTCGATACGTGAGCGACGGGGAAACTCCACGTTGGCGCTCAAAGGGCGCGTTTATTGTTCTGTCAGCTGATCGTTTGGGTATTTAATGCCGCCGTGTTCGTCAAGAGGCCACTCTTGAGTGCCAGCGAGAAGAGTTTTCTCTGCCAGCTCATTTTCACGGCGCCATTATGAGAACTGAAATGACTCCCTTGGTCCTGTCGTATCAGGAAGCTGACGACATATTGGAGCATTTGGTGGACAACTTTTTTAACGAGGTACCCAGTGATGATGATCTTTATGTTCCGTCTCTTTACGAACTGTATGATCTTGATGTGGAGTCTGCCGGTGAAGATAATAATGAACAGGCGGTGAATGAGTTTTTTCCCGAATCGCTTATTTTAGCTGCCAGTGAGGGGTTGTTTTTACCGGAGCCTCCTGTACTTTCTCCTGTCTGTGAGCCTATTGGGGGCGAATGTATGCCACAACTGCACCCTGAAGATATGGATTTATTGTGCTACGAGATGGGCTTTCCCTGTAGCGATTCGGAAGACGAGCAAGACGAGAACGGAATGGCGCATGTTTCTGCATCCGCAGCTGCTGCTGCCGCTGATAGGGAACGTGAGGAGTTTCAGTTAGACCATCCAGAGTTGCCCGGACACAATTGTAAGTCCTGTGAGCACCACCGGAATAGTACTGGAAATACTGACTTAATGTGCTCTTTGTGCTATCTGCGAGCCTACAACATGTTCATTTACAGTAAGTGTGCTATGGGAGGTGGGAGGTGATTTTTTTTTCTTAAGCAGTGAAAAATAATATTTTGTTGTTTTTAGGTCCTGTTTCCGATAATGAGCCTGAACCTAATAGCACTTTGGATGGCGATGAGCGACCCTCACCCCCGAAACTAGGAAGTGCGGTTCCAGAAGGAGTAATAAAACCTGTGCCTCAGCGGGTGACTGGGAGGCGTAGATGTGCTGTGGAAAGCATTTTGGATTTGATTCAAGAGGAAGAAAGAGAACAAACAGTGCCTGTTGATCTGTCAGTGAAACGCCCTAGATGTAATTAATGGACTTTGAGCACCTGGGCAATAAAATAGGGGTAATGTGGTTTTTGTGAGTCATGTATAATAAAACTGGTTTCGGTTGAAGTGTCTTGTTAATGTTTGTTTGGGCGTGGTTAAACAGGGATATAAAGCTGGGTTGGTGTTGCTTTGAATAGTTCATCTTAGTAATGGAGTTGGAAACTGTGCTGCAAAGTTTTCAGAGCGTTCGCCAGCTCTTGCAGTATACCTCTAAAAACACTTCAGGTTTTTGGAGGTATCTGTTTGGCTCTACCTTAAGCAAGGTGGTAAATAGGGTGAAAGAAGACTATAGAGAGGAATTTGAAAACATATTGGCCGACTGTCCAGGGCTTTTGGCTTCACTAGACCTTTGTTACCACTTGGTGTTTCAGGAAAAAGTGGTCAGATCCTTAGATTTTTCATCTGTGGGACGAACGGTTGCTTCTATTGCTTTTTTGGCAACCATATTGGATAAATGGAGCGAGAAATCCCACCTGAGTTGGGATTACATGCTGGATTACATGTCAATGCAGCTGTGGAGGGCATGGCTGAAGAGGAGGGTTTGCATTTACTCGCTGGCGCGGCCTTTGACCATGCCGCCGCTGCCGACGTTGCAAGAGGAGAAGGAGGAGGAGCGGAACCCTGCGGTGGTGGAGAAGTAAACATGGAACAACAGGTGCAAGAAGGCCATGTACTTGACTCTGGCGAAGGGCCTAGTTGCGCAGATGATAGAGATAAGCAGGAAAAAAAAGAAAGTTTAAAGGAAGCTGCTGTTCTTAGTAGGCTAACTGTTAATCTGATGTCCCGCCCGCGTTTGGAAACTGTATATTGGCAGGAGTTGCAGGATGAATTTCAGCGGGGTGATATGCATTTACAGTACAAATACAGTTTTGAACAATTAAAAACCCACTGGTTAGAGCCATGGGAGGATATGGAGTGTGCTATTAAAGCTTTTGCTAAATTGGCCTTACGTCCTGATTGTAGCTACAGAATTACTAAAACAGTAACCATTACTTCATGCGCCTATATTATAGGTAACGGGGCAATAGTTGAGGTAGATACAAGCGACAGAGTTGCTTTTAGATGTCGAATGCAGGGTATGGGCCCAGGGGTGGTGGGTTTGGATGGAATTACATTTATAAATGTTAGGTTTGCTGGAGATAAGTTTAAAGGCATTATGTTCGAAGCTAATACCTGTCTTGTCTTGCATGGTGTTTACTTTCTTAACTTTAGTAACATTTGTGTAGAGTCTTGGAATAAGGTTTCTGCTAGGGGCTGTACTTTTTATGGATGTTGGAAGGGTTTGGTGGGTAGACCAAAAAGTAAACTGTCTGTAAAAAAGTGTTTGTTTGAAAAATGTGTACTTGCTTTAATTGTAGAGGGGGATGCACATATTAGGCATAATGCAGCTTCAGAAAATGCCTGTTTTGTATTATTGAAGGGAATGGCTATTTTAAAGCATAATATGGTTTGTGGGGTGTCTGATCAAACTATGCGACGTTTTGTTACCTGTGCTGATGGAAATTGTCATACCTTAAAAACTGTTCATATTGTGAGCCACAGTAGACATTGTTGGCCTGTATGTGATCATAACATGTTTATGCGCTGTACCATACATTTAGGCTTAAGGCGGGGTATGTTTAGACCTTCCCAATGTAACTTCAGCCACTCAAACATTATGCTGGAACCTGAAGTGTTTTCTAGAGTGTGTTTAAATGGGGTATTTGATTTATCTGTGGAATTATGTAAGGTTATAAGATATAATGATGATACTCGACATCGTTGCCGACAGTGTGAGTGTGGTAGCAGTCATCTAGAACTTCGTCCCATTGTGCTAAATGTAACTGAGGAGCTGAGAAGTGACCACCTTACCCTGTCTTGCCTGCGGACTGACTATGAGTCAAGTGATGAAGACGACAACTGAGGTAAGTGGGTGGAGCTAGGTGGGATTATAAAAGGCTGGAAGTCAACTAAAAATTGTTTTTGTTCTTTTAACAGCACGATGAACGGAACTACTCAGAACAACGCTGCGCTTTTTGATGGAGGGGTTTTTAGCCCTTATTTGACTTCCAGGTTACCATATTGGGCCGGAGTACGTCAGAATGTGGTAGGATCTACAGTGGACGGTCGACCTGTGGCACCTGCAAATTCATCAACATTAACCTATGCAACTATTGGACCCTCGCCTTTGGATACCGCCGCCGCCGCTGCAGCTTCCGCGGCCGCTTCTACGGCTCGCAGTATGGCAGCTGATTTCAGCTTCTACAATCACTTGGCTTCGAATGCTGTGACACGCACCGCAGTTCGAGAGGACATTCTGACTGTTATGCTTGCCAAGCTTGAAACTCTAACTGCTCAGCTGGAAGAGCTATCGCAAAAGGTTGAGGAATTAGCTGATGCTACTACCCATACCCCAGCCCAACCTGTAACCCAATAAAGAAAAAACTTAAATTGAGATGGTGTTATGAATCTTTATTGATACTTGTTTTTTCTGACATGGTAAGCTCTTGACCACCGTTCCCTATCATTAAGAACACGGTGAATGTGTTCCAGTATTTTGTAAAGATGAGCCTGTATATTAAGGTACATTGGCATTAGGCCATCTTTGGGATGAAGGTAGGACCATTGAAGGGCTTCATGTTCCGGGTTAGTGTTGTAGATAATCCAGTCATAGCAACAACGCTGGGCATGGTGATTAAATATATCTTTTAACAACAAGCTAATTGCTAATGGAAGACCTTTAGTATAGGTATTGATAAAACGGTTAAGCTGGGTGGGATGCATCCGAGGTGACATGATATGAAGTTTTGATTGTATTTTGAGATTGGCAATGTTACCTGCCAAATCTCTTCTTGGATTCATATTGTGGAGAACCACGAAAACGGTGTAGCCAGTACACTTGGGAAATTTGTCATGGAGTTTAGAAGGAAAGGCATGGAAAAACTTGGAAACGCCTTTGTGACTTCCCAAATTTTCCATACACTCATCCATTATTATGGCAATTGGACCGCGAGCAGCGGCTTGAGCAAAAATGTTTTCTGGATCAGAAACATCATAGTTGTGGTCTAGAGTTAGGTCATCGTAGGACAACTTAACAAATTTAGGACACAGCGTTCCAGATTGTGGAATAATAGTTCCCTCTGGTCCTGGGACATAATTTCCCTCACAAATTTGCATTTCCCAAGATTTAATTTCAGATGGGGGAATCATGTCCACTTGCGGAACAATAAAAAAAACAGTTTCTGGAGCAGGTGTAACCAGCTGGGCAGAAAGCAAATTACGCAACAACTGAGACTTCCCACAGCCAGTGGGTCCATAAATTACCCCAATTACAGGTTGCAAGTGATAGTTTAACGAGGTGCAGCTGCCGTCTTCGTGGAGAAGCGGAGCCACTTCATTCATCATTTGTCGGACGCGGATGTTTTGCTTGGCCAGTTCCCCTAACAGACGCTCTCCGCCTAAGGAAAGTAACTCTTGTAAAGATTTGAAATTTTTAAGTGGCTTTAGGCCATCGGCCATAGGCATGTGGTCCAGGGTTTGCTTCAGCAGTTGCAAGCGATCCCATAGCTCAGTTATATTTTCTATGCCATCTCGATCCAGCAAACTTCCTCGTTGCGGGGGTTTGGCTGGCTGTTGCTGTAAGGAACGAGGCGGTGAGCATCCAAATGGACGAGGGTTTTGTCCTTCCAGGGACGTAATGTGCGCGTCAGGGTTGTTTCGGTCACGGTGAATGGATGCGCTCCTGGTTGAGCGCTGGCCAGTGTGCGCTTTAAACTGAGGCGGCTGGTGCTGAAGCGCGTGTCTTCTCCCTGTGCTTCGGCAAGGTAGCATTTTAACATAAGATCATAAGACAAAGCCTCTGTAGCGTGGCCTTTAGCCCGTATTTTTCCTTTGGAGGTGCTCCCGCAGTGAGGACACTGAAGGCATTTAAGGGCGTACAGTTTTGGAGCCAAAAAAACAGATTCTGGAGAATAAGCATCTGCGCCACAATAACTACAAACAGTTTCACATTCAACTGACCAGGTCAGCTCAGGACATGATGGATCAAAAACAAGTTTCCCTCCGTACTTTTTGATGCGTTTCTTACCTTGCGACTCCATAAGGCGGCGTCCTTTCTCTGTGACAAAAAGACTGTCAGTGTCTCCGTATACAGATTTAAGGGGTCTATCCTTCAGTGGTATTCCGCGGTCCTCCTCGTACAGGAATTCTGACCACTCTGACACAAAAGCTCTAGTCCAAGCAAGTACAAAGGAAGCCACATGGGAAGGGTACCGATCGTTGTTAATTAAAGGGTTAGAACTTTCTAAGGTGTGTAAACACATGTCTCCTTCTTCAGCGTCCATGAATGTGATTGGTTTGTAGGTGTAAGTCACGTGTTCACAATTTTCTGGTGGTGGGCTATAAAAAGGGGCGGGTCCTTGGTCTTCATCGCTTTCTTCTGCTTCGCTGTTTACGAGCGCCAACTGGTTGGGTGAGTACACGCGCTCAAAGGCAGGCATTACCTCTGTACTCAACGTGTCAGTTTCTATAAACGATGAGGATTTGATGTTTAATCGCCCCGCTGCAATTTCTTTCATTAGGCTTTCTTCCATTTGATCAGAAAAAACTATTTTTTTGTTATCTAGTTTGGTAGCAAAAGATCCGTACAAGGCATTGGAAAGCAGCTTGGCTATAGATCTTAGGGTTTGATTTTTGTCCCTATCGGCCCGTTCTTTTGCGGCAATATTGAGTTGCACATATTCGCGTGCCAGGCATTTCCAGGTGGGGAAAATGGTGGTGCGCTCGTCAGATAGCAAGCGTAAGCGCCACCCGCGATTATGCAGTGTAACCAGATCTACGCTGGTAACTACTTCACCGCGCAAGCTTTCATTGGTCCAGGCTAAACGACCGCCTTTTCTAGAACAAAAAGGAGGAAGAACATCCAACTGATTTTCATCTGGGGGGTCGGCATCTATAGTAAAAATGCCAGGACAAAGATTTTTGTCAAAATAATCAATTTTGCAAGTGTAATTTTCCAGCGCCACCTGCCATTGCCGCACGGCCAATGCCCGCTCATAGGGGTTAAGGGGAGGACCCCAAGGCATGGGGTGTGTGAGGGCCGATGCATACATGCCGCAAATATCATATACATATATGGGCTCTTTTAGTACTCCTATGTAAGTAGGATAGCACCTGCCGCCACGAATGCTGGCGCGAACGTAGTCATATAGCTCATGTGAAGGCGCCAGGATGTTGGGCCCAAGATGTGTGCGCTGTGGTTTTTCGGCGCGGTACAAAATTTGTCTGAAAATTGCATGAGAGTTAGAGGAAATGGTAGGACGCTGAAACACATTAAAATGTGCCGCGTCAAGACCCACTGCGTCAGTAACAAACTGGGCGTATGAGCTACGCAGTTTTTCTACCAATGAGGCAGTCACAAGTACATCCAGGGCACAATAGTTTAATGTTTCCCCGATAAGATTGTAATTTTTTTCTCCTTTTTTTTTCCATAGTTCTTGATTTAGGAGGTATTCCTCCTTATCCTTCCAGTACTCCTCCAGGGGAAACCCATTTGCATCTGCACGGTAAGAACCAAGCATATAAAACTGATTTACCGCCTTGTACGGACAACATCCTTTTTCTACAGGCAGGGCATACGCTTGTGCAGCCTTTCTTAAAGATGTATGAGTAAGAGCAAAGGTATCTCTGACCATTACTTTTAAATACTGGTATTTAAAATCTTGGTCGTCACACCCTCCGTGTTCCCACAGTAGGAAGTTAGTTCGCTTTTTGTAGTGGGGATTGGGAAGGGCAAAAGTAATATCATTAAATAATATTTTGCCAGCTCTTGGAATAAAATTTCTAGAAATTTTAAAGGGTCCAGGGACGTCCAAGCGGTTATTGATTACCTGAGCGGCAAGAACAATTTCATCAAATCCATTAATATTGTGTCCTACTATATACAACTCTACAAATCTTGGCTCACCCTTAATTGCAGGGGCTCTTTTAAGATCTTCGTAGGAAAGATCTTCAAGCGCGACTAGTCCGTTTTCTTCTTGAGCCCATTGAGACAAGTGTGGATTTTTTTGTAAAAAAGTCATCCAAAGATCAGTAGCTAAGGAGGTTTGTAAGCGGTTTCTATAGGTACGAAACTGTTGACCGACCTTCATTTTTTCTGGGGTTAAGCAGTAGAAAGTAGTAGAGTCTTTTTCCCATTGGTCCCATCCAAGTTCTAATGCAAGTTGTAAGGCATGTTTGACAAGATTGTCATCCCCAGACAGTTTCATCACCAGCATAAATGGGACAAGTTGCTTTCCAAATGCCCCCATCCAGGTGTAGGTTTCTACATCATAGGTAATAAAAAGGCGCTCAGTGCGAGGATGCGAACCGATTGGGAAAAAGTGGATCTCCTGCCACCAGTTGGAAGAATGGCTGTTGATGTGATGAAAGTAGAAATCTCGTCGGCGGACAGAGCATTCATGCTGATGTTTGTAAAAGCGTGCGCAGTGTTCGCATCGTTGCACGGGCTGTATCTGTTGAATGAGGTGTACCTGGCGGCCTCGCACCAGAAAGCAGATGGGAAAATCAATACCACTTGGCAGCTGCCGTTCGTCCTCTTCCTCTTCTGCTGCATTGCCACTACCGTTTGGATCCTCGAAAGCGAGAACGGAGAGGGTGACGGTGCCCCTCGAGCTGCATGTCCAGATTTCAGCACGAGAGGGGCGGAAACGGGAAATCAGGGCGTACAGCCTGGAGCTGTCCATGGTATCAGTCAGAGAGAAAAGCATGTCCGCGGGGACAGCGCGCAAGTTGACTTCGCACAGGCGGGTAAGAGCAGGCTGGAGGTGCAGGTAATACTTAATTTCTAGAGGCGTGCCGTTGGCAGAGTCTATTGCGTGAAGTATTCCATGAGCCCGGGGACTAACCACGGTTCCACGGTGCACTTTTCCAATGCGCCTGCTTAAAATCGGCGGCGCGGACGAGCTCCCGGAGGAAGCGGCGGTTCGGGTCCTGCGGGAAGCGGGGGAAGCGGTATGTCGGCCTGACGCTCTGGCAGGGGAAGGTGTTGAGCCCGAAGTTGACTGGCATGGGCGACTACCCGGCGATTGATATCTTGAATCTGTCGGCGTTGTGTAAACACTACCGGCCCTGTTGTTTTGAACCTGAAAGAAAGTTCAACAGAATCAATCTCAGTGTCATTTACTGCAGCCTGTCTTAAAATCTCCTGAACGTCGCCTGAGTTATCTTGGTAGGCAATTTCTGCCATTAATTGATCAATTTCTTCCTCCTGGAGGTCTCCATGTCCCGCACGTTCAATAGTGGCTGCAAGGTCATTAGATATCCGACTCATAAGCTGTGAAAATGCGTTTAGTCCAATTTCGTTCCAGACTCGGCTGTATACTACCCCTCCTTCGCTGTCCCGAGCGCGCATAACCACTTGCGCCAAGTTGAGTTCCACGAGCCGTGCGAACACGCCGTAGTTGCGCAAGCGCTGAAACAGGTAGTTTAAGGTGGTGGCAACGTGTTCTGAGACGAAGAAATACAGAATCCACCGACGAAGCGTCAGCTCGTTGATGTCACCTAAGGCTTCAAGACGTTCCATGGCTTCGTAAAAGTCTACTGCAAAATTGAAAAACTGGGAGTTGCGAGCTGCCACCGTCAATTCTTCTTCCAACAGACGAATAAGCTCGGCCACCGTCTCGCGCACTTCTTGCTGAAATGCGCCCGGAACTATTTCTTGTTCTTCCTCTTCTACCTCCATTATTTCTTCCTCGACCACAGGTGGTGGGGGTTGTCTTCTTCGACGCCGGCGAACGGGCAGCCTGTCTACAAATCTTTCAATCATTTCGCCGCGACGGCGGCGCATAGTTTCGGTTACTGCTCGACCGTTTTCACGTGGTCGTAACTCAAAAACTCCACCTCTAAGTTCTGTTTCATGTAAAATGGGAAATGAGGCGTTGCGAGGGGCGTTAGGTAGGGATACAGCGCTGATTATGCATTTTATTATTTGCTGCGTAGGAACTCCGCGCAAGGAGCTAAGCGTCTGCATATCCACCGGGTCGGAGAACCTTTCAAGAAAGGCATCTAGCCAGTCACAGTCACAAGGTAGGCTAAGTTTTGTTTCTTCTAAAGTACCAGGAAGCTGAGCAATGCTACTAATAATGTAATTGAAGTAAGCTGTTTTAAGCCCACGAATGGTTTTAAGAAGCACCACATCTTTGGGTCCGGCTTGTTGAATTCGCAGGCGGTCTGCCATTCCCCACACGTCACTTTGACATCGTCCAAGATCTTTGTAGTAGTCTTGCATTAACCTTTCCACCTCTACCTCGCGGTTTCCGCGATCAGCCATGTGCGTGCTTCCGTAGCCTTGCAGCGGTTGTAATAAAGCTAAATCTGCCACTACCCGTTCCGCAAGCACTGCCTGTTGAATTTGGGTAAGGGTGGTTGCAAAGTCATCCACATCTACAAAGCGGTGATAAGCTCCTGCATTAATGGTGTAGCTGCAGTTTGTCATTACTGACCAATTAACAGTTTGCGTGCCTGGCTGTACAGTTTCTGTGTATCGCAAGCGTGAGTAAGCCCGAGAGTCAAAAACATAGTCATTGCAGGTGCGCACTAGGTATTGATAGCCCACAAGGAAATGAGGAGGAGGTTCGCGATACAACGGCCAGCCAAGCGTAGCCGCAGCACCTGGAGCGAGATCTTCCAACATGAGGCGGTGGTATTCATATATGTATCTGGACATCCATGTGATGCCGGCAGCGGTAGTTGTTGCTCGCATAAATTCGCGGGCTCGGTTCCAAATATTGCGCAGGGGTAAAAAGCGTTCAATAGTTGCCACGCTTTGACCGGTCAGGCGTGCGCAGTCTTGAATGCTCTGGACATGGAAAAAATGAAAGTCGGTAAGCGACTCCCTTCCGTGGTTTGGTGGAAAAGTCACAAGGGTACCATAGCGAGGAACCCCGGTTCGAAACCGGCAGGATCCGCTATGAGCACAAGTGAGGCGCTTGCGCGTTGAACCCGGCCAAGGACCCCCAGACACGGAGAGGAGTCTTTTTTTATTTATTTTTTCTTAGATGCATCCTGTCCTGCGACAAATGCGACCTCAGCCCAGGGCAACCACGGCCTCAGCAGCGGTGGCGCTTTCGGGCTCTGGCGAACAGGAAGAGCCTCAATGTCCTACATTGGAGTTGGAAGAAGGAGAAGGCATAGCCCGATTGGGCGCCCACTCTCCTGAGCGTCACCCAAGGGTGCAGCTCGCCCGGGACAGTCGCGTGGCATTTGTGCCTCGTCAGAACATGTTTCGCGACAACAGCGGGGAGGAAGCTGAGGAAATGCGAGACTGCAGGTTTAGGGCCGGTCGCGAGCTGCGCCGCGGATTTAATCGCGAGCGACTGCTGCGTGAGGAGGACTTTGAGCCAGATGAACATTCGGGGATTAGTTCTGCACGGGCCCATGTATCAGCAGCCAACTTAGTAACAGCATATGAACAAACGGTTACAGAGGAACGTAACTTTCAAAAAAGCTTTAATAACCATGTGCGCACACTAATAGCGCGAGAAGAAGTAGCCATTGGTTTAATGCATCTTTGGGACTTTGTAGAAGCTTATGTACATAATCCAGCAAGTAAACCCCTAACTGCCCAGCTGTTCTTAATAGTTCAACATAGTAGAGACAATGAAACTTTTAGGGATGCAATGCTTAACATAGCTGAACCCCAGGGTCGGTGGTTACTCGATTTAATTAACATTCTGCAGAGCATTGTGGTTCAGGAACGCAGTCTTAGTTTGGCAGACAAGGTGGCCGCCATTAATTACTCCATGTTAAGTTTGGGAAAGTTTTATGCTCGTAAAATCTACAAAAGTCCGTATGTTCCCATTGACAAGGAAGTGAAGATAGACAGCTTTTATATGCGCATGGCTTTAAAGGTACTAACATTAAGCGACGATCTTGGAGTGTACCGCAATGACCGAATCCACAAAGCAGTAAGCGCCAGTCGCCGCAGAGAGCTAAGCGACAAAGAGCTTATGCATAGCTTACAAAGGGCGCTGACGGGAGCAGGAACAGAGGACGAGTCGTTCTTTGATATGGGCGCAGACCTACGGTGGCAGCCAAGCGCTCGCGCTTTGGAGGCAGCTGGAGTGGCGTCTGCTGACGTCACTGGCGATGACGATGACGAAGACCAGTACGAGGACTGATCGGCCGTACCTTTTGTTAGATGCAGCGACCGGCGATCATCGCGGAGAGGGCTCCTAACCTGGATCCCGCGGTTTTGGCGGCCATGCAAAGCCAGCCTTCTGGCGTTACAGCTTCAGATGACTGGACAGCGGCCATGGATCGTATTATGGCTTTAACGGCGCGCAGTCCTGATGCTTTTCGCCAGCAGCCCCAAGCTAACCGCTTTTCGGCCATTTTGGAAGCAGTAGTGCCGTCTCGTACTAACCCTACTCACGAGAAAGTGTTAACCATTGTAAATGCTTTGTTGGATAGCAAAGCCATCCGCAAAGATGAGGCTGGTTTAATATACAACGCTTTGCTTGAGCGCGTGGCACGCTATAACAGTACCAATGTGCAGGCTAACTTAGACCGGATGGGTACAGATGTAAAGGAGGCGCTGGCTCAACGAGAGCGCTTTCATCGCGATGGTAATCTTGGTTCGCTAGTAGCATTAAACGCTTTTTTGAGTACTCAGCCGGCTAATGTTCCGCGTGGTCAGGAAGATTATACAAACTTCATCAGCGCCTTGCGACTAATGGTTACTGAAGTGCCTCAAAGTGAAGTGTATCAGTCTGGACCCGATTACTTTTTTCAAACGTCCAGGCAGGGTTTGCAAACCGTAAACTTAACTCAGGCTTTTAAAAATTTGCAAGGTTTGTGGGGGGTTCGTGCTCCAGTAGGCGATCGTTCAACTTTGTCCAGTTTACTAACACCAAACTCGCGCCTATTACTGTTGCTAATTGCCCCCTTTACCAACACCAACAGTTTAAGTCGAGATTCATACCTGGGTCACTTAGTTACTTTGTACCGCGAAGCCATTGGTCAAGCGCAGGTAGACGAACAAACTTATCAAGAAATAACCAGTGTTAGTCGCGCACTGGGCCAGGAGGACACTGGCAGTTTAGAGGCCACACTTAACTTTTTACTAACTAACCGTCGCCAGCAAGTGCCTCCTCAGTACACTTTAAATGCGGAAGAAGAACGCATATTGCGCTATGTACAGCAATCTGTAAGTTTGTATCTTATGCGTGAGGGTGCCACCCCCAGTGCCGCCTTAGACATGACAGCGCGCAATATGGAGCCGTCCTTCTACGCTTCCAATCGAGCTTTCATTAATCGCTTGATGGATTACCTTCACCGCGCTGCGGCCATGAACGGGGAATACTTTACAAATGCAATTCTAAATCCGCATTGGTTGCCCCCTCCTGGATTTTACACTGGTGAATTTGATTTGCCGGAAGGAAATGATGGCTTTTTGTGGGATGATGTTACGGACAGTCTGTTTAGTCCTGCAGTTATTGGACACCATGGTAAAAAGGAAGCAGGTGATGAAGGTCCCTTGCTTGACTCTCGGGCGAGTTCTCCATTCCCCAGTTTAACTAGTTTACCCGCCAGTGTTAACAGCGGTCGTACCACCAGACCCCGACTAACAGGTGAAAGTGAATACTTAAATGACCCCATCTTGTTTCCAGTGCGCGACAAAAATTTTCCCAACAATGGCATAGAAAGTTTGGTAGATAAAATGTCTCGCTGGAAAACATATGCACAAGAGCGGCGAGAATGGGAGGAAAGACAGCCAAGACCAGTTCGCCCTCCTAGGCAACGTTGGCAGCGACGCAAAAAAGGGGCACATGCGGGGGATGAAGGAAGCGATGACTCAGCTGACGACAGTAGTGTATTAGATTTAGGAGGGTCAGGAAACCCATTTGCTCATTTGCGCCCACAGGGTTGCATAGGGTCATTGTATTAAATTGAATAAAAGCATACTTACCAAAGCCATGGCGACCAGTGTTCGTCTTATTTTCCTTCTTCCGTTAGCTGTGAAATGAGGCGCGCGGTGGAACTGCAGACAGTGGCTTTTCCTGAGACACCACCTCCCTCTTACGAAACCGTGATGGCAGCGGCGCCACCCTACGTGCCTCCCCGCTATTTGGGTCCTACGGAGGGAAGAAACAGTATCCGTTACTCGGAATTGTCACCGTTGTACGATACCACTCGAGTGTACTTGGTGGACAACAAGTCTTCTGACATTGCTTCATTGAATTACCAGAATGATCACAGCAACTTTTTAACCACTGTAGTGCAAAATAATGACTATTCCCCTATAGAGGCTGGCACGCAAACTATTAACTTTGATGAAAGGTCTAGATGGGGTGGAGATTTAAAAACCATCTTACATACCAACATGCCAAACGTGAACGATTTTATGTTTACCACCAAATTTAAGGCCAGGGTAATGGTGGCTAGGAAAACAAACAACGAAGGCCAAACCATTTTAGAATATGAGTGGGCAGAATTTGTGCTACCCGAGGGTAACTATTCGGAAACCATGACTATTGACTTAATGAACAATGCTATTATTGAGCATTATTTGCGAGTAGGAAGACAGCATGGAGTGCTGGAAAGTGACATTGGAGTTAAGTTTGACACCAGAAACTTTCGTCTGGGTTGGGACCCCGAAACCCAATTAGTAACTCCGGGAGTGTACACTAATGAGGCTTTTCATCCAGATATAGTACTGCTTCCAGGTTGCGGGGTTGATTTTACAGAGAGCAGATTAAGCAACATACTAGGTATAAGAAAGAGGCAGCCGTTTCAGGAAGGATTTGTGATTATGTATGAACACTTAGAGGGAGGCAATATTCCAGCTCTTTTGGATGTAAAAAAATACGAAAACAGTCTGCAGGATCAAAACACTGTAAGAGGAGACAACTTTATTGCCTTAAATAAGGCTGCTAGGATTGAACCGGTTGAAACAGACCCCAAAGGACGCAGTTACAACTTGCTTCCAGACAAAAAAAATACTAAATATCGCAGCTGGTATTTGGCATACAACTACGGAGACCCAGAAAAAGGAGTTCGGTCATGGACTCTACTAACAACTCCAGATGTAACAGGCGGCTCCGAACAGGTGTACTGGTCCCTACCCGATATGATGCAAGATCCGGTGACTTTTCGCTCCTCGCGTCAAGTTAGCAACTATCCTGTAGTTGCAGCAGAATTACTGCCAGTTCATGCTAAAAGCTTCTACAACGAGCAAGCCGTCTACTCACAGCTTATTCGCCAGTCAACCGCGCTTACGCGCGTGTTTAATCGCTTTCCCGAGAACCAGATACTGGTGCGTCCACCAGCCGCTACCATCACTACCGTCAGTGAAAACGTTCCCGCCCTTACAGATCACGGGACCCTGCCGCTGCGTAGCAGTATCAGTGGAGTTCAGCGAGTCACCATCACTGACGCCCGCCGCCGGACCTGTCCCTACGTTTACAAAGCACTGGGCATAGTTTCTCCACGAGTGCTTTCTAGTCGCACTTTTTAAAAAAGTGTGGTAACATGTCCATTTTGGTTTCGCCAAGTAACAACACGGGCTGGGGACTGGGTGCCGCCCGCATGTATGGAGGAGCTAAAACAAGGTCTAGCCAACATCCAGTGCGCGTACGCGGACATTACCGAGCTCCATGGGGCGCGCATACCCGAGGACGCACTGGTCGCACCACTGTAGACGATGTTATTGACTCGGTAGTGGCCGATGCTCGCAAGTACCGCGCGCCCGCTGAAACAGCAGGGTCTACTGTTGATGCAGTAATTGATGAGGTAGTGGCAAACGCGCGGGCTTATGCAAGGCGCCGCAGACGGCTGCGTCGCCGGCGTAGACCAACCACCGCCATGCGCGCGGCCAGAGCGTTGGTTCGACGGGCCAGGCGCATTGGGCGGCGAGCTATGATGCGGGCAGCCAGGCGGGCTGCAACGCCTGCCGGTCGAGCGCGGAGACGGGCCGCAGCTGCGGCCGCAACAGCTATTGCAAACCTAGCTGCTCCGCGACGAGGAAATGTATACTGGGTGCGCGACTCAGTGACCGGGACGCGTGTGCCAGTTCGTACGCGTCCACCTCACCCTTAGAAGACAAAGAGTGACTCAATGTCTGTTATGTATGCCCAGCATGACCAAACGCAAGTTCAAAGAAGAGCTGCTGCAGGCCTTAGCGCCTGAAATATATGGCCCATCGGATAACCTTACCAAGCGCGATATCAAGCATGTTAAAAAACGGGAAAAAAAAGAGGAAGAAGTCGCCGCGGCGTCAGCAGACGGCGTCGAGTTTGTGCGCTCATTTGCGCCCAGACGTAGGGTACAGTGGAAGGGACGGCAAGTAAAACGCATTTTGCGACCGGGCACCACAGTGGTTTTTTCTCCCGGAGAGCGAACGATTATGCGTCCCCTAAAGCGCGAGTACGACGAAGTGTACGCAGACGATGACATTTTGGAGCAAGCGGCACAACAGACTGGGGAATTTGCATATGGAAAAAAAGGGCGTTACGGAGACAAAATTGCTATTCCTTTGGACGAGGGAAATCCAACACCCAGTTTAAAGGCTGTCACTTTGCAACAAGTGTTGCCCGTCCTTGGGCCTTCGGAAGAAAAGCGTGGAATTAAAAGGGAAGCCATGGATGAATTGCAGCCTACAATGCAACTGATGGTGCCTAAGCGGCAAAAGTTAGAGGACGTACTAGAGCACATGAAGGTGGATCCTAGCGTACAGCCAGATGTAAAAGTACGTCCGATAAAAAAGGTAGCTCCAGGATTGGGAGTTCAAACAGTGGACATTCAAATTCCTGTGCAAACTGCATTGGGTGAAACTATGGAAATCCAAACTTCGCCAATAAAAACAACGGTGAACGCAAGCGTGCAAACAGACCCTTGGTACCCGCCAGTGCTTTCAACAAAAAAAAAGCGTCACTACAGACAAACAAGTTCGCTTTTGCCAGACTACGTTTTACATCCTTCCATTGTGCCCACGCCTGGGTACCGTGGGACAACTTTTCAGCGCCGAGCCACAGCCCCTAGCCGTAGACGAGGTCCATCACGCCGTAGACGTCGACGCAAAGCCACTTTAGCCCCAGCGGCAGTACGTCGCGTTGTACAAAGGGGGCGCACACTAATACTTCCATCCGTGCGTTACCACCCTAGCATTCTCTAACAAGCTGCGCTGCCGTTTTTTCAGATGGCTCTTACTTGCCGAATGCGCATACCCATTCCAGGATACAGAGGACGACCCCGCCGGAGGAAAGGGCTGACCGGGAACGGTCGATTTCGGCGGCGTAGTATGCGCAGACGCATGAAGGGTGGGGTGCTGCCCTTCCTAATTCCACTTATTGCTGCGGCCATTGGAGCCGTTCCCGGAATTGCCTCAGTAGCCTTGCAGGCTTCTCGAAAAAATTAAAATAAAATAAAACTTCCAACTTATTACTGGTACTATGACTGTTTTATGCAGACTAAATGGAAGACATCAATTTTTCGTCGCTGGCCCCGCGACACGGCACGCGGCCGTACATGGGCACCTGGAACGAGATCGGCACGAGCCAGCTGAACGGGGGCGCCTTCAATTGGAACAGTATCTGGAGCGGTCTTAAAAATTTTGGTTCCACGATTAAGACATATGGCACCAAGGCGTGGAACAGCCAAACCGGCCAGATGCTAAGGGACAAGTTAAAAGACCAAAATTTTCAACAGAAAGTTGTAGATGGTCTGGCTTCGGGAATTAATGGAGTTGTAGACATAGCCAATCAGGCTGTACAGAAAAAAATTGCCAACCGTTTAGAGCCGCGGCCCGACGAGGTAATGGTAGAGGAAAAGCTGCCACCTCTAGAAACTGTGCCCGGATCCGTTCCAACCAAAGGAGAAAAGCGGCCACGGCCGGATGCAGAGGAAACCTTAGTAACGCACACAACAGAACCGCCGTCCTATGAGGAAGCAATAAAACAAGGAGCCGCTCTGTCACCTACCACCTATCCCATGACCAAGCCTATTTTACCCATGGCTACTAGAGTGTATGGAAAAAACGAAAATGTGCCTATGACCCTTGAGCTGCCTCCTTTGCCAGAACCCACTATCGCGGATCCCGTAGGTTCCGTTCCTGTTGCATCTGTTCCAGTTGCATCGACAGTGAGCCGTCCAGCAGTGCGGCCTGTTGCCGTGGCTAGCTTGCGAAACCCACGATCCAGTAATTGGCAAAGTACCCTAAACAGTATTGTGGGACTGGGAGTAAAGTCTCTCAAACGCCGACGCTGCTACTAACATTAAAAGACGAGTGTTAATTCCCATCTGTGTATACGCCTCCTATGTTAGCGCCAGAGGACCAACGCGTGAATCGCAGTCACCACCAGCGCTTTCAAGATGGCCACTCCCTCGATGATGCCGCAGTGGTCTTACATGCACATCGCCGGTCAGGATGCCTCGGAGTACCTGAGTCCCGGTCTGGTGCAATTCGCCCGCGCCACGGACACCTACTTCACCCTGGGAAACAAGTTTAGAAACCCCACCGTGGCTCCCACCCATGATGTTACCACCGATCGCTCGCAGCGTCTGACGCTGCGTTTTGTGCCCGTGGATCGGGAAGATACTACCTACTCCTACAAGGCTCGCTTTACGCTGGCTGTGGGTGACAACCGCGTGTTAGACATGGCTAGTTCTTACTTTGACATTCGAGGGGTACTGGATCGTGGTCCCAGTTTTAAGCCCTATTCCGGAACCGCCTACAATTCTTTGGCACCAAAAGGCGCTCCTAATGCTTCACAATGGTCAGATAACGCTAAGCTTAATACCTTTGCTCAGGCGCCGTATCTTAGCGACACTATCACCGCCGCCGATGGTATTAAAGTTGGAACAGACACCGCCCAGGCAGGCGCGGCGGTGTATGCCAACAAAACTTATCAGCCAGAGCCGCAAGTAGGACCAAGTGAATGGAACACCAGCATTGAAAACGTTAAAGCTGGCGGGAGGGCATTAAAGCAAACCACTGCAATGCAGCCGTGCTATGGCTCCTACGCTCGTCCAACCAACGAACACGGAGGACAATCCAAGGATGACAACATTGAACTTAAGTTCTTTGATTCAGCTAACAATGCAGCAAACACTGCTCAAGTTGTGTTCTATACCGAAGACGTAAACCTTGAAATGCCAGACACGCATCTTGTGTTTAAGCCTACTGTTACCAATGGAACAATTGCTTCTGAGTCGCTGTTGGGACAGCAAGCAGCGCCAAATAGAGCAAACTACATTGCATTCAGAGATAATTTTATTGGCCTGATGTATTACAACAGTACAGGCAACATGGGTGTATTGGCCGGGCAAGCTTCCCAACTTAACGCAGTAGTAGACCTGCAAGACAGAAATACAGAGCTGTCATACCAGTTAATGCTGGATGCTTTGGGAGACAGAACACGGTACTTTTCCTTGTGGAATTCCGCAGTGGACAGTTACGACCCTGACGTTCGCGTTATTGAGAATCACGGGGTAGAGGATGAACTACCAAATTATTGCTTTCCTCTTAGCGCAGTAGGTGAAATAAAAAATTACAAAGGCATTAAGCCAGATAACGGAGGAGGAGGTGGCTGGACTGCCGACAACACTGTCAGTGAAGCAAACCACATAGGCATTGGGAATATAGCCGCCATGGAAATTAATTTGCAGGCTAATTTGTGGAGAAGCTTCTTGTACTCAAATGTGGGCTTATACCTACCAGACGACTTAAAATACACTCCAGGAAACATAAAACTACCTGATAACAAGAACACCTACGAGTACATGAACGGGCGTGTGACTGCCCCGGGGTTGGTGGATACCTATGTCAATATCGGCGCTCGCTGGTCCCCAGATGTGATGGATAATGTAAACCCTTTTAACCACCACCGAAACGCAGGGTTGCGCTACAGATCCATGTTGCTAGGCAATGGGAGATTTGTTCCTTTTCACATTCAGGTGCCGCAAAAATTTTTTGCCATCAGAAATTTGTTGCTGTTGCCCGGTTCCTACACTTACGAATGGAACTTTAGAAAGGATGTAAACATGATTCTTCAGAGCACACTGGGAAATGATCTTCGGGTGGACGGAGCCAGCGTTCGCTTTGACAACATTGCCCTGTATGCTAACTTTTTTCCCATGGCACATAACACAGCTTCTACTTTAGAAGCCATGTTAAGAAATGACACCAACGACCAGTCTTTTAACGATTATTTGTGTGCTGCAAACATGCTGTATCCCATCCCAGCTAACGCCACCAGCGTGCCCATTTCAATACCTTCGCGAAATTGGGCGGCATTTAGAGGCTGGAGCTTTACTCGCCTAAAAACTAAAGAAACTCCTTCCCTGGGTTCAGGGTTTGACCCCTACTTTGTATACTCTGGAACCATTCCCTATTTAGACGGCACCTTTTACCTAAACCACACTTTTAAGAAGGTGTCAATCATGTTTGACTCCTCCGTGAGTTGGCCTGGAAATGACCGTTTGCTAACCCCAAATGAATTTGAAATAAAGCGTTCTGTGGATGGGGAGGGATACAATGTGGCCCAATGCAATATGACTAAGGATTGGTTCCTAATACAAATGCTTAGTCATTACAACATTGGATACCAAGGTTTTTACATTCCAGAGAGCTACAAGGACCGCATGTATTCTTTCTTTAGAAACTTTCAGCCCATGAGTAGGCAAGTTGTGGATACCACAGAATATAAGAACTACAAAAAAGTAACCGTAGAGTTTCAACATAACAACTCAGGATTCGTGGGATACCTGGGCCCCACTATGCGGGAGGGACAAGCTTACCCCGCCAACTATCCCTACCCTCTTATAGGCAAAACAGCTGTGGAAAGCATCACACAGAAAAAGTTTCTATGCGATCGTGTTATGTGGCGCATCCCATTTTCTAGTAACTTCATGTCTATGGGGGCGCTAACGGATCTTGGGCAAAATATGCTGTACGCAAACTCAGCCCATGCTCTAGACATGACATTTGAGGTGGATCCAATGGATGAGCCTACCCTTCTTTATGTTTTATTTGAAGTTTTCGACGTGGTACGCATTCACCAGCCACACCGCGGCGTCATTGAAGCGGTCTACCTGCGCACGCCCTTCTCGGCGGGTAACGCTACCACCTAAGAAGGCACCCTCCCAGACTGCTGTAATGGGTTCAAGCGAACAGGAGCTGACGGCCATTGTTCGAGATCTAGGCTGTGGACCCTATTTTTTGGGAACCTTTGACAAACGTTTTCCGGGTTTTGTGTCTCGCGACCGCTTATCATGTGCTATTGTTAACACTGCCGGTCGCGAAACTGGGGGCGTACACTGGCTGGCTTTTGGATGGAACCCCAAATCGCACACTTGCTATTTATTCGATCCATTTGGATTTTCTGATCAACGACTAAAACAAATCTATCAGTTTGAGTACGAAAGTCTGTTGCGCCGTAGTGCGCTAGCGGCCACTAAAGACCGATGCGTTACCCTAGAAAAGTCAACCCAAACTGTACAAGGACCGTTTTCTGCAGCGTGCGGCCTGTTTTGTTGTATGTTCTTACACGCTTTTACTCACTGGCCTGACCATCCAATGGATAAAAATCCCACTATGGACCTACTTACTGGGGTGCCTAATTGTATGCTACAAAGTCCTCAGGTAGTGGGCACATTGCAACGCAATCAGAATGAATTGTATAAATTCTTAAACAATCTGTCCCCTTACTTTCGTCACAACCGCGAGCGCATAGAAAAAGCTACATCTTTTACTAAAATGCAAAATGGACTCAAATAAACGTGTACACAATGCATTAATAATAAAACCATTTTATTAGCTCATTGGAGTACAAGCTTGACTGTTTTATTAAAAATCAAATGGCTCTTCGCGACAGTCGCCGTGGTTGGTGGGCAGGGATATGTTTCTGTACTGCAAACGCTGATGCCACTTGAATTCTGGAATAACAAGCCTAGGGGGGGAGCCGTCAAAATTTTCTCCCCACAGCTGGCGCACAAGTTGCAGGGCGCCCATAACATCAGGAGCAGAAATCTTGAAGTCGCAATTAGGGCCAGCATTGCCGCGCGCATTGCGATAAACTGGATTTGCGCACTGAAAAACCAACAAACACGGATACTTAATACTGGCTAACGCTCCAGGGTCGGTTACTTCGTTGATATCAATGTTATCCACATTGCTGAGGTTAAAAGGAGTGATTTTACACAGTTGACGCCCCATCCGTGGCAGGCCATCTTGCTTGTTTAAACATTCGCAGCGCACTGGCATAAGGAGACGTTTTTGCCCATGTCGCATGTGAGGGTAGTCGGCCAGCATAAAAGCTTCAATTTGCCTAAAAGCTATTTGAGCCTTCATTCCTTCAGAATAAAACAAGCCGCAGGACTTTCCGGAGAAAGAATTATTCCCGCAGCCAACATCATGAAAACAGCAGCGGGCATCGTCGTTTTTAATTTGAACTACATTACGCCCCCAGCGGTTTTGCGCCACCTTGGCTTTCGAGGGGTTCTCTTTCAACGCTCGTTGCCCACTTTCGCTGGTTACATCCATTTCCACCAAATGCTCTTTGCGCACCATCTCCATTCCATGCAGGCATCTAAGCTCCCCTTCGCGCTCGGTACACTTATGCTCCCACACGCAGCAACCGGTGGGTTCCCAGGAATTCTGTTGGACACCGGCATAAGCTTGCATATATCCTTGCAAAAAGCGTCCCATGAGCTCCTGAAAGGTTTTTTGGGATGAAAAAGTCAGCTGCAAACCGCGCTTTTCTTCGTTGAGCCATGTTGTGCATATTTTCTTGTACACGCTGCCCTGATCCGGCAAAAAACGAAAGGTGGCGCGCTCGTCGTGATCCACATGGTACTTTTCCATTAGCATAGCCATGGCTTCCATGCCTTTTTCCCAAGCTGAAACTAGGGGCTGGCTTGCCGGATTGCGAACAACAACAACATTCTTTTCATTTTCGTCGCTGTTTTGAGCGGAAGCCTTCAAAACGTGTACCTGCCTGGTTTCCATTTTTTGAAAAGACTGAGAACCGTCTGCATGATGCATAATGCGGACGGGCGGCATGCTGAAACCCATTACTCCTAAAACTGCTCTTGGTGGTTCTGCCTCTTCTTCTTCTGCACTCTCTGGGGAAAGAGGTATCGCAGCCATAGATTTCTTGACTTTTTTCTTTGGAGGTAAAGGCACAGCTTCCAGTTCTTCTTCGCTTTCGGAATCCAGAAAGTATCTGCCCATTTTTGGCGGCGGCGGCTGAGCGCTGCGGTCTGGGGTGCGCTCCCTCTGTGAGTGCTGATTGCTGGCCATTATTTAATCCTAGGCAAAGAAACACATGATGGATCTGGAGCCACAGGAAAGCTTAACCGCCCCCACCGCTCCCGCCATTGGCGCTACGGCTGTCATGGAGAAGGACAAAAGTCTACTCATACCCCAAGACGCACCGGTTGAGCAGAACTTGGGCTACGAGACTCCCCCCGAGGAATTTGAAGGCTTTCTTCAAATCCAAAAGCAACCAAATGAGCAAAACGCTGGGCTCGAGGACCATGACTACCTAAACGAGGGAGATGTCCTGTTTAAACATCTACAGCGACAAAGCACTATCGTTCGCGACGCCATATCTGATCGCTCTTCAATACCAGTTTCAATTGCAGAACTATCTTGCATCTACGAACGCAACCTGTTCTCCCCACGTGTGCCCCCTAAACGGCAAGCCAACGGCACATGCGAGCCAAATCCTCGCCTTAACTTCTACCCAGTTTTTGCAGTGCCAGAAGCACTGGCAACATACCATATTTTCTTTAAAAATCACAAAATACCCCTATCCTGTCGAGCTAACCGCAGCCGCGCAGATGAGCTTCTTGCTTTAAGGGCTGGCGCTTCCATACCTGGGATTGTGTCCTTGGAAGAGGTGCCTAAAATTTTTGAAGGTTTAGGTCGGGATGAAAAACGAGCAGCAAATGCCCTGCAAAAAGAAAATGAACAAAATCACCATGGGAATAGTGCTCTAATAGAACTGGAAGGTGACAATGCCCGCCTGGCAGTTTTAAAGCGCAATATTGAGGTTACTCACTTTGCCTACCCGGCAGTAAATCTTCCGCCAAAGGTAATGAGCGCAGTGATGAATCAGCTACTAATTAAGCGAGCCCAACCCATTGACAAAGATGCAAACTTGCAAGACCCGGAGGCAACAGATGATGGAAAGCCGGTTGTAAGCGACGAGCAATTAACTAAGTGGTTGGGAACAGACAATTCCAACGAACTACAACAGCGGCGTAAACTCATGATGGCCGCCGTACTTGTAACTGTGGAACTCGAGTGCATGCATCGTTTTTTCTCCGACATCACCACATTGCGCAAAATTGAGGAATGTCTTCACTACACTTTCCGCCATGGCTACGTGCGCCAAGCCTGTAAAATTTCTAATGTGGAGCTGAGCAATCTAGTTTCTTACATGGGCATCTTGCATGAAAACCGATTGGGACAGAACGTGCTACACTCAACACTACGCGATGAAGCACGCAGAGATTACGTGCGAGACTGCATTTACCTTTTCCTGTTACATACCTGGCAAACTGGGATGGGTGTTTGGCAGCAATGCTTGGAAGAAAAAAACCTTCGAGAACTAAACAAACTGTTAGACAGAGCACTAAAATCCCTATGGACCGGTTTTGACGAACGGACAGTAGCTGCAGAGCTAGCTGACATAATTTTCCCAGAAAGGTTAATGATAACCTTGCAAAACGGCTTGCCTGACTTTATGAGTCAAAGTATGCTGCACAATTATCGCTCTTTTATATTAGAGCGTTCTGGGATGCTTCCTAGCATGTGTTGTGCACTTCCTTCAGATTTTGTGCCTATATATTTTAGAGAGTGCCCCCCTCCCCTGTGGAGCCACTGCTACTTACTACGACTTGCTAACTACCTAGCTTACCACTCAGACCTTATGACAGATTCAAGCGGCGAAGGCCTAATGGAGTGTCACTGCCGCTGCAATCTTTGCACCCCCCACCGTTCTTTGGTTTGCAATACTGAACTATTAAGTGAAAGTCAAGTCATTGGTACCTTCGAAATGCAGGGACCGCAGTCTGACAGCAATTTCACGACGAACCTAAGACTTACCCCTGGGCTTTGGACTTCTGCCTACCTGCGCAAATTTGAACCCCAAGATTACCACGCCCACAGTATCAATTTTTACGAAGACCAATCCAAACCCCCAAAAGCGCCACTAACGGCTTGCGTCATTACGCAGGGAAAAATTCTAGCCCAATTGCATGCTATTAAGCAAGCGCGCGAAGAGTTTTTACTTAAAAAAGGACACGGAGTGTACCTTGATCCCCAAACCGGCGAGGAACTAAACCTTCCATCACCTTTGTGTGCTACTGCGTCTCCCCATTCGCAGCATGTCCCCGAAAGCCGCAAAACAGGCTATTGCGCAGCAACGCTCAAAGAAACAGCAGCAACGGCAGGAAATCTGGGAGGAAGAATCTTGGGAGAGTCAGGCAGAGGACGAGGTCGAGGACTTGGAAGAATGGGAGGAGGAGGAGGCGGACAGCCTAGACGAGGATCCAGAGGAGGAGGAGGAAGGTTCCAAGGACGGAGCGACCGCCGCAAAACCGTCGCTTTCAACCAAGCCCTCTCCAATGAAACCCGCTGTGAGCAAATCTCAGAAAGCCAGCCGTAGATGGGACACCATTGAAACCAGCGCCGCAAACTTGGGTAAGAATCGCAAGCAGGCGCGTCGGGGCTACTGCTCATGGCGGGCTCACAAAAGTAATATTGTAGCCTGCTTGCAGCACTGCGGGGGGAATATCTCATTTGCAAGGCGGTATTTGCTATACCATGATGGAGTGGCGATTCCAAGGAATGTCCTCCATTACTACCGTCATCTCTACAGCCCCTTTGAAGAGCTCGACAAGGAACCGACCTGCAACAGCCAAGCGGCCCACTAGAATCGGCAACAGCAGCAACAAGGAAAGTCCTGAGGCGCGCGAGTTAAGAAAACGCATTTTTCCCACTTTATATGCTATTTTTCAGCAGAGTCGAGGTCAAGAACACGAACTGAAAATAAAAAACCGTTCCCTGCGTTCACTTACCCGCAGCTGTCTCTACCTCAAAAGCGAAGATCAGTTGCAACGCACCTTGCAGGACGCAGAAGCTCTGTTCAATAAATACTGCTCCCTCTCGCTTAAAGAGTAAAAAAAGCCCGCGCGCGGACTTTCAACAGGCGGGAAAAGTGACGTCACAACAAGATGAGTAAAGATATTCCCACGCCTTACATGTGGAGCTTTCAACCGCAAATGGGACTGGCGGCCGGCGCGGCTCAAGACTATTCTAGCAAAATGAATTGGTTAAGCGCCGGACCCCACATGATTTCCAGGGTGAATGGGGTACGAGCCCGGCGTAACCAAATACTGCTAGAACAAGCCGCTCTCACCGCTACACCACGTAATCAACTTAACCCTCCCTCTTGGCCAGCTGCCCTGATATATCAGGAAAATCCCCCTCCTACCACTGTACTTTTGCCTCGCGACGCCCAGGCCGAAGTCCATATGACTAACGCTGGGGCACAGCTTGCGGGCGGTGCACGTCACAGTTTCAGGTATAAAGGTCGCACTGAGCCCTATCCGTCTCCAGCTATAAAAAGAGTACTCATCAGAGGGAAAGGTATTCAGCTGAACGACGAAGTCACATCGCCATTGGGAGTCAGACCCGACGGAGTGTTTCAGCTCGGAGGGTCCGGACGTTCCTCCTTTACCGCTCGTCAAGCCTACCTGACACTACAGAGCTCATCCTCAGCTCCGAGATCTGGTGGTATTGGAACTCTCCAATTTGTGGAGGAATTTACTCCATCTGTTTACTTCAATCCTTTTTCGGGCTCGCCTGGACACTATCCTGACGCCTTCATACCCAACTTTGACGCAGTGAGTGAATCTGTGGATGGCTATGATTAATGTCTAATGGAGCGGCTGACAGAGCGCGGCTGCGACATTTAGACCACTGTCGCCAACCTCACTGCTTTGCTCGAGACATCTGTGTCTTTACCTACTTTGAGCTTCCAGAGGAGCACCCCCAGGGGCCAGCTCACGGTGTCAGAATAACAGTTGAAAAAGGAATTGATACACACCTCATTAAATTTTTCACCAAACGCCCGCTATTGGTGGAAAAAGATCAAGGAAATACTATATTAACTTTATATTGCATTTGTCCTGTTCCCGGATTACATGAAGATTTCTGCTGTCATTTGTGTGCTGAATTTAATCATCTGTAGTGGCGCTGTACCGCCTGAAGAAGAACCTAACTGTCATCCGCATTTAAGCAACATTAAAATCAACCTTTCGATCCCTCATATCACTCTTCGCTGCAGTTTTTTTTCCACACATCTCACCTGGACCTTTAACGGAAAACACGTTACCAATACAGATATAAAGTTTAAACTACACAAAGAAAACATCACTCTATTTCAACCTATTAACCTGGGATACTACCGCTGCTCAGCTCCACCCTGTACGCAAGCATTTTTTGTTGCTCCAGTTATTGACAAACGCCCTGCTCCGACAACAGCTGCTGTCACTGAGCACATCACCGAGGCAGTTTCTCCTTCTAAAGGTACAGAGGAAATTGTGTACTTTTCAAACTTTACAAACCACTTAGTTTTAAATTGTTCCTGTTCTAACTCCTTAATTTCATGGTTTGCTAACAGCTCTCTGTGCAAAACTTTCTACCAAGGAAAACTTTTGTATTCTGCTAAACTCACATTGTGTAACCAGAGCACCCCTTCCCACCTTACTCTATTGCCACCTTTTGTTGCCGGTCGTTACTTTTGCATAGGAGCTGCACGTACTAGCCCCTGTCAACAGCATTGGAATTTAACTTACTGTCCCCCACCAGTGTCGCCCTTTGTGATCAATACTGAATATTTAGACTATAATCCCTTGCTTGCTTACGGCGGTCTCGCAGCTCTTATTTTATTCCTGATTTCTAACTTGTTTCTAGTGCAACATTTGTATTCATACTAACAATGCTTTCCATTTTTCTTTTATTTCTCTTTTCTTTACCTTCTGGCTTGTATGCTCAAACAGCCGAAAGACCACTAAAAGTCGTGGTGGAAGCTGGCCATAATGTAACCCTTCCCCACCTTTCTGGTTCACACCAAACTGGCCATGTTACTTGGCTAGTAGAGACATCAGATTATGGTTCAGCTTCTCCAGACAACTTCATTTTCAGTGGACAAAAACTATGCCAGTTTACTGACAGAACCATGGTGTGGCCTTATTACAATTTACATTTTAACTGTGAAAATTATGACCTTAATCTGTTTTGGCTTAAGGTGGAAAATTCGGCTATTTACAACGTTAAAAATACAGTCAATGCTTCTGAAACAAATATTTACTATGATTTAAGAGTAGTACAAATTTTTCCACCTAAATGCATCATTACTTCAAAGTACCTTACAAACGATTATTGTCACATTACAATTAACTGCACTAACTCTGATTACCCCAATAAGGTTGTGTTTAATAATGTCAGTCGATGGTACTACGGATACGGTAAGGGCAGCCCAACCCTTCCCAACTACTTTATAACTAACTTTAATGTTTCAGGTATTACTAAAAGCTTTAATCACACTTACCCTTTTAATGAGCTCTGTGATTATCCCACATCCCAATCTCAACACAGTTTAACACATACAGTAAGCACAGTAATCTTTTTAGGAATAATTGGCTTCAGCATTTTGATTATTATAGCAGCCTTTATTTATCTGTGCTGGCATAGAAAATCTTTGTGTGTTTCTAAAACAGAACCTCTTATGCCGATTCCTTACTAGTTTTCTTTTTTCTTACAGTATGGTCACGGTTCTTCTCATCTTTTTATGCCTGCCAGTCATTTTTTCTTCTTCGACTTTTGCCGCAGTCAGTGACCTTGATCCCGAGTGTTTAGCCCCCTTTGCGGTGTACCTGATTTTCACATTTGTGACTGCTACCTGCGTCTGCAGTATTATTACTCTGCTAATCACCTCGCTCCAATTTTTTGATTACTACTACGTGAGAATTGTTTACCGCAGACACCACCCCCGTTACCAAAACCCTCAAATTGCGGCTCTTTTGCAGCTCCAACCATGAAAACAGCATTAGTTCTTTTCTTTATGTTAATCCCAGTTTGGGCTAGTTCTTGTCAACTACATAAACCATGGAATTTTTTAGATTGTTATACTAAAGAAACAAACTACATAGGCTGGGTTTATGGAATTATGTCTGGCTTAGTATTTGTCTCCTCTGTAGTTTCTTTACAACTGTATGCGCGCCTTAATTTTAGTTGGAATAAGTATACTGATGATCTTCCCGAATATCCAAACCCCCAGGATGATTTACCCCTAAATATTGTATTTCCAGAGCCCCCGCGTCCTCCTTCTGTTGTTAGCTATTTTAAGTTCACCGGTGAAGATGATTGAACCTGATCTAGAAATTGATGGAAGAATCACCGAACAGAGGCTCCTCACTGATCGCGCTAGGCGACGCCAACAGGATCAAAAAAATAAAGAGTTAATTGATTTACAAACCGTGCATCAGTGTAAAAAAGGACTTTTTTGCCTGGTAAAACAAGCTACCCTTCGCTATGAATCTTTACCAGGCAAAGAACATCAACTGTGCTACACGCTGCCCACTCAGCGACAAACCTTTACTGCAATGGTGGGCTCGGTACCTATTAAAGTGTCCCAACAAGCAGGAGAACAAGAAGGCTCTATTCGGTGCCTATGTGATAACCCTGAATGTTTGTACACTTTAATAAAAACACTGTGCGGTTTAAGAAATCTTTTACCAATGAATTAAATAAATTACTTACCGGAAATCTGAAAATACATCATGGTCTCCGTGTACTCTTATAAAATTTCCCTCTTCCCAACTGTCAAACCTGACAGACTTGCAAACAGCAAACTTTCTCCAAATCTTAAATGGAAGGTCAGATTCTTCTTCCCAATCCCTACCCACCATCTTCATCTTTTCTAGATGAAGCGCAGCAGAACCCAGTATGCTGAAGAAACAGAAGAAAATGATGACTTCAACCCCGTTTACCCTTTTGACCCATTTGACACATCAGACGTACCCTTTGTTACACCCCCTTTTACTTCTTCCAATGGTCTTCAAGAAAAACCACCAGGTGTATTAGCACTTAATTACAAAGACCCCATTGTAACTGAAAATGGAACCCTTACACTCAAGCTAGGGGACGGAATAAAACTTAATGCCCAAGGTCAACTTACAGCTAGTAATAATATCAATGTTTTGGAGCCCCTTACCAACACCTCACAAGGTCTTAAACTTTCTTGGAGCGCCCCCCTAGCAGTAAAGGCTAGTGCCCTCACACTTAACACAAGAGCGCCCTTAACCACAACGGATGAAAGCTTAGCCTTAATAACCGCCCCTCCCATTACAGTAGAGTCTTCGCGTTTGGGCTTGGCCACCATAGCCCCTCTAAGCTTAGATGGAGGTGGAAACCTAGGTTTAAATCTTTCTGCTCCCCTGGACGTTAGTAACAACAATTTGCATCTCACCACTGAAACTCCCTTAGTTGTAAATTCTAGCGGTGCCCTATCTGTTGCTACTGCAGACCCCATAAGTGTTCGCAACAACGCTCTTACCCTACCTACGGCAGATCCGTTAATGGTGAGCTCCGATGGGTTGGGAATAAGTGTCACTAGTCCCATTACAGTAATAAACGGTTCCTTAGCCTTGTCTACAACTGCTCCCCTCAACAGCACAGGATCCACTTTAAGTCTGTCTGTTGCCAATCCTCTGACTATTTCACAAGACACATTGACTGTTTCCACTGGTAACGGTCTTCAAGTGTCGGGGTCTCAATTAGTAACAAGAATAGGGGATGGTTTAACATTCGATAATGGGGTCATGAAAGTAAACGTTGCCGGGGGAATGAGAACTTCTGGCGGTAGAATAATTTTAGATGTTAATTATCCCTTTGATGCGAGCAATAACCTGTCCTTAAGACGGGGATTGGGACTAATTTATAACCAATCTACAAACTGGAACTTAACAACTGATATTAGTACCGAAAAAGGTTTAATGTTTAGTGGCAATCAAATAGCTCTTAATGCAGGTCAGGGGCTTACATTTAATAATGGCCAACTTAGGGTTAAGTTGGGAGCTGGACTTATTTTTGATTCAAACAATAACATTGCCTTAGGCAGCAGCAGCAACACTCCATACGACCCTCTGACACTGTGGACAACTCCTGACCCACCACCAAACTGCAGCCTCATACAAGAGCTAGATGCAAAACTCACCCTGTGCTTAACAAAAAACGGATCTATTGTTAATGGCATTGTAAGTTTAGTGGGTGTTAAGGGTAATCTCCTAAATATCCAAAGTACTACTACCACTGTAGGAGTGCATTTAGTGTTTGATGAACAGGGAAGATTAATCACATCAACCCCTACTGCCCTGGTTCCCCAAGCTTCGTGGGGATATAGACAAGGCCAATCAGTGTCTACCAATACTGTTACCAATGGTCTAGGTTTTATGCCTAATGTGAGTGCTTACCCTAGACCAAATGCCAGTGAGGCTAAAAGCCAAATGGTAAGTCTCACGTACTTACAGGGAGATACATCTAAACCTATAACAATGAAAGTTGCATTTAATGGCATTACGTCGCTAAATGGATACTCTTTAACATTCATGTGGTCAGGTCTATCAAACTATATAAATCAGCCTTTCTCTACACCATCCTGCTCCTTTTCTTACATTACCCAAGAATAAAAACACACACAAAACACAAATTGCGTACTTATTGTTTATTTTTTTTTTTTTTTACACTATACGCGTGGTTAAACTGCCTCCTTCCCATTTTACCTTGTATACCTCCCTTTCCCCCTTTGTAGCTGAAAACAACTGCACTTGAATATTTCGACTTAGGTTTTTTGGCGTTAGCGTCCACACAGTTTCTTTACGGGCAAAGCGAGGGTCGGTGATGGAAACGAATCCCTCGCCCGCACAGTCACTCAAGCGGCATTCCCCATCCAAAACCAGGTCCATGATTTTATCCTACAAAAAGTAACAACAGTCAGTGTCCATCAGCCGCCCAAGGATTCTCTCGTTGATTATAATCTCCAAATAAAATTGCTCGATGATGCATAATTAAACCCTTTAGCAGTTGCTGACGATAACGTTCATGCCGACTATGTTTTAGAGGGCGAACAGTGTTTTCAGCAATTACTTGAACAACTTTTAACATTAGCAGTCTGGTACGACGAGCGCAACAGCGCATGCGTATCTCACTTAAGTCTTTACAATAATCACAACACAGCACTAACATGTTATTTAAAATTCCATAATTAAAGGCGCTCCATCCAAAACTAACTTTTTCTAACGCTAACCAGGCATGGCCATCATACATAATTTTAAAGTAAATTAAATGGCGACCTCTAACAAAGGTGCTTCCCACATACATCACCTCTTTAGGCATTAAATGGTTAACAACCTCCCGATACCAAAAACACCTTTTGTTAATTAAGGCGCCATATACGGCCATTTTGAACCAGCGTCCCAAAAGCATCCCAGCTGACATACACTGTAGTGAACCCGGACGCTGGCAATGACAATGAATAAGCCACCGCTCATGACCATGTAATAATTGAGTAACTTCAACATTTATAGTGGCACAACACATACATACACTCATGTATTTTTTCAAAATAAACATCTCATAATCAGTTAGAATCATATCCCACGGTATTGGCCATTCCTGCAGCACTGTAAAACCTACACATGAAGGAATGCCTCTTACCTCACTTACATTATGTAAAGTCAGACTATTACACTCAGGCCATAAAGAATTTTCCGAAGTACTCAACGTAGCTTTTGACTGTTCCTCACAGGGCGGTAGTTGGTACTTGTTGTATGGTGCCAATCTGTAGCGATACCGTCTGTCGCGCTGCATCGTAAACAACAGACTTGCGAGCGTCTTCGTACTTAAAAAAACAAAACCACGTACGACCACTGGTTATCGCACCTCGTCCTTTTTGTTTGCAGCGTTGGCGTTCCGTCAAAAAAGCAAAGTACAACCACTCTCGCAGGCTTGCTAAAATGTATTCAGCTTCAGGTGTTATCTTCAAATCATGATGTTTAATAAAGCGCAGAGTATCCACACAGGATGCATGGGCTAAACCAAGCCATGCTATGCAGGCAGCCGTGTCCCGACTTACAGGAGGAGGAGGAATACAAGGTAGAGGCATAAAAACTTAATCAAGACGGTCAGCAAGGATTTGAATGCGTAAATCTCGCAGGTGGCAGCGATCGCCTCCGCTGTGCTGGTGAAAGATCACAGCCAGATCAAATTGTAAGCGATTTTCCAAATGTTCAACAACAGCTTCTAAAAGAGCCACAGCTCTGATTTCGATAAACAAAAGCAAAGCAAATGCATTATCATGAAACTCTTCTATCATCAAACTGCCTGACTGAACCATTCCCAGGTAATTTTCATTCTTCCACTGTTGTATTATTTGAACACACTGATTTTGCAGGTTTAAACCGTGAATATTAAAAAGCTCTGTAAGGGCGCCCTCCACCGCCATCCGCAGGCAGTACTTCATATTTGCTGAAAAAAGTCTGGATCTTCAAACACCTGCAGTAAATTCAGTAGATTTACATTAGGCTCCACACCTTGGTCTCGCAGCTGACATCTTAATGCCAGTTGTATAAAATCATACAAATCAGAAGCCAGCAGCAAAGAAAGTTCACCTCCAGGTACAAGTTCCGGAGTTCCCACAGAACATACAACTTGCACAAATGGACCCATATTAGTAAGCGTGGCGCCAACGTAGACATCGCGCATAGGAGGAGTTAAATAATGCATTACCAGCAGCCAAAACTCAGGTAGCACGTCTTTAAGAAACGTCACCACCTCAAAATCTAAGCCATGCAAATAGTTCCGTAAAGACTCCGGAAACAACACGGAGTAATGAACAAGCGACCTCTGAAACATGCTTTAGGTTAGCCTGAAAAATAAAAATATGTTAAATTAAAGATGCCTGGCAAACGGGTGGAAAAACAACTCTACTTAAAAGCAAGCGCGCGACTGGCTGCTTTGCGCGAACATCGCAAAACACGTCGGAATGATTAAACAACAAAACACTGAGCTCCATTCTTGAGCCTGGATAAAGCGTTTCAGCGCCAACAAAAACCCCTCTGGCGTTCATGTCGCATAATGAAAACAATGTTCCCAAATATCCAGGAGGAATATCAACTGCTATGTGCAAATATAAAAGCACAACTCCATGTGGAGGTATAACAAAATTCGCAGGAGAAAATAACACATAAGCATTAGAGTCGCCCTCTTGTTTAGGCAACATAGCCCCAGGTCCCGTAAAATACACATAAAGAGTCTCAAAAGCAGCCATAATGCCTTACCAGAAAAACAGTACAAAGCCAGGCACAGCAGACACAATCTGCCGCAAGTGCGCACCTTTAATACTGAAAAATAGTGACGTAAATGGCCAAAGTTCGCCTACACAACACAAAAAAAACCCCAAAAGCCCGCGAAAAAAATCACTTCCGCATATGACTCGGCATAATACGGTGTTCTCACGACACGTCACATCCGGCGCGCCCGGCTCCCACGCCGCGCCCCACTTCCTCATCCGCCCAAACTTACAAGCACGCCAAAGCCACACCTCCACCCAATCAAATTACACACTACGCCCACTTCATTTTAATATTGGCACTAGTCCAGTATAAGGTATATTATTAGATAGG

>

CGTATCCAATAATATACCTTATACTGGACTAGTGCCAATATTAAAATGAAGTGGGCGTAGTGTGTAATTTGATTGGGTGGAGGTGTGGCTTTGGCGTGCTTGTAAGTTTGGGCGGATGAGGAAGTGGGGCGCGGCGTGGGAGCCGGGCGCGCCGGATGTGACGTTTTAGACGCCATTTTACACGGAAATGATGTTTTTTGGGCGTTGTTTGTGCAAATTTTGTGTTTTAGGCGCGAAAACTGAAATGCGGAAGTGAAAATTGATGACGGCAATTTTATTATAGGCGCGGAATATTTACCGAGGGCAGAGTGAACTCTGAGCCTCTACGTGTGGGTTTCGATACGTGAGCGACGGGGAAACTCCACGTTGGCGCTCAAAGGGCGCGTTTATTGTTCTGTCAGCTGATCGTTTGGGTATTTAATGCCGCCGTGTTCGTCAAGAGGCCACTCTTGAGTGCCAGCGAGAAGAGTTTTCTCTGCCAGCTCATTTTCACGGCGCCATTATGAGAACTGAAATGACTCCCTTGGTCCTGTCGTATCAGGAAGCTGACGACATATTGGAGCATTTGGTGGACAACTTTTTTAACGAGGTACCCAGTGATGATGATCTTTATGTTCCGTCTCTTTACGAACTGTATGATCTTGATGTGGAGTCTGCCGGTGAAGATAATAATGAACAGGCGGTGAATGAGTTTTTTCCCGAATCGCTTATTTTAGCTGCCAGTGAGGGGTTGTTTTTACCGGAGCCTCCTGTACTTTCTCCTGTCTGTGAGCCTATTGGGGGCGAATGTATGCCACAACTGCACCCTGAAGATATGGATTTATTGTGCTACGAGATGGGCTTTCCCTGTAGCGATTCGGAAGACGAGCAAGACGAGAACGGAATGGCGCATGTTTCTGCATCCGCAGCTGCTGCTGCCGCTGATAGGGAACGTGAGGAGTTTCAGTTAGACCATCCAGAGTTGCCCGGACACAATTGTAAGTCCTGTGAGCACCACCGGAATAGTACTGGAAATACTGACTTAATGTGCTCTTTGTGCTATCTGCGAGCCTACAACATGTTCATTTACAGTAAGTGTGCTATGGGAGGTGGGAGGTGATTTTTTTTTCTTAAGCAGTGAAAAATAATATTTTGTTGTTTTTAGGTCCTGTTTCCGATAATGAGCCTGAACCTAATAGCACTTTGGATGGCGATGAGCGACCCTCACCCCCGAAACTAGGAAGTGCGGTTCCAGAAGGAGTAATAAAACCTGTGCCTCAGCGGGTGACTGGGAGGCGTAGATGTGCTGTGGAAAGCATTTTGGATTTGATTCAAGAGGAAGAAAGAGAACAAACAGTGCCTGTTGATCTGTCAGTGAAACGCCCTAGATGTAATTAATGGACTTTGAGCACCTGGGCAATAAAATAGGGGTAATGTGGTTTTTGTGAGTCATGTATAATAAAACTGGTTTCGGTTGAAGTGTCTTGTTAATGTTTGTTTGGGCGTGGTTAAACAGGGATATAAAGCTGGGTTGGTGTTGCTTTGAATAGTTCATCTTAGTAATGGAGTTGGAAACTGTGCTGCAAAGTTTTCAGAGCGTTCGCCAGCTCTTGCAGTATACCTCTAAAAACACTTCAGGTTTTTGGAGGTATCTGTTTGGCTCTACCTTAAGCAAGGTGGTAAATAGGGTGAAAGAAGACTATAGAGAGGAATTTGAAAACATATTGGCCGACTGTCCAGGGCTTTTGGCTTCACTAGACCTTTGTTACCACTTGGTGTTTCAGGAAAAAGTGGTCAGATCCTTAGATTTTTCATCTGTGGGACGAACGGTTGCTTCTATTGCTTTTTTGGCAACCATATTGGATAAATGGAGCGAGAAATCCCACCTGAGTTGGGATTACATGCTGGATTACATGTCAATGCAGCTGTGGAGGGCATGGCTGAAGAGGAGGGTTTGCATTTACTCGCTGGCGCGGCCTTTGACCATGCCGCCGCTGCCGACGTTGCAAGAGGAGAAGGAGGAGGAGCGGAACCCTGCGGTGGTGGAGAAGTAAACATGGAACAACAGGTGCAAGAAGGCCATGTACTTGACTCTGGCGAAGGGCCTAGTTGCGCAGATGATAGAGATAAGCAGGAAAAAAAAGAAAGTTTAAAGGAAGCTGCTGTTCTTAGTAGGCTAACTGTTAATCTGATGTCCCGCCCGCGTTTGGAAACTGTATATTGGCAGGAGTTGCAGGATGAATTTCAGCGGGGTGATATGCATTTACAGTACAAATACAGTTTTGAACAATTAAAAACCCACTGGTTAGAGCCATGGGAGGATATGGAGTGTGCTATTAAAGCTTTTGCTAAATTGGCCTTACGTCCTGATTGTAGCTACAGAATTACTAAAACAGTAACCATTACTTCATGCGCCTATATTATAGGTAACGGGGCAATAGTTGAGGTAGATACAAGCGACAGAGTTGCTTTTAGATGTCGAATGCAGGGTATGGGCCCAGGGGTGGTGGGTTTGGATGGAATTACATTTATAAATGTTAGGTTTGCTGGAGATAAGTTTAAAGGCATTATGTTCGAAGCTAATACCTGTCTTGTCTTGCATGGTGTTTACTTTCTTAACTTTAGTAACATTTGTGTAGAGTCTTGGAATAAGGTTTCTGCTAGGGGCTGTACTTTTTATGGATGTTGGAAGGGTTTGGTGGGTAGACCAAAAAGTAAACTGTCTGTAAAAAAGTGTTTGTTTGAAAAATGTGTACTTGCTTTAATTGTAGAGGGGGATGCACATATTAGGCATAATGCAGCTTCAGAAAATGCCTGTTTTGTATTATTGAAGGGAATGGCTATTTTAAAGCATAATATGGTTTGTGGGGTGTCTGATCAAACTATGCGACGTTTTGTTACCTGTGCTGATGGAAATTGTCATACCTTAAAAACTGTTCATATTGTGAGCCACAGTAGACATTGTTGGCCTGTATGTGATCATAACATGTTTATGCGCTGTACCATACATTTAGGCTTAAGGCGGGGTATGTTTAGACCTTCCCAATGTAACTTCAGCCACTCAAACATTATGCTGGAACCTGAAGTGTTTTCTAGAGTGTGTTTAAATGGGGTATTTGATTTATCTGTGGAATTATGTAAGGTTATAAGATATAATGATGATACTCGACATCGTTGCCGACAGTGTGAGTGTGGTAGCAGTCATCTAGAACTTCGTCCCATTGTGCTAAATGTAACTGAGGAGCTGAGAAGTGACCACCTTACCCTGTCTTGCCTGCGGACTGACTATGAGTCAAGTGATGAAGACGACAACTGAGGTAAGTGGGTGGAGCTAGGTGGGATTATAAAAGGCTGGAAGTCAACTAAAAATTGTTTTTGTTCTTTTAACAGCACGATGAACGGAACTACTCAGAACAACGCTGCGCTTTTTGATGGAGGGGTTTTTAGCCCTTATTTGACTTCCAGGTTACCATATTGGGCCGGAGTACGTCAGAATGTGGTAGGATCTACAGTGGACGGTCGACCTGTGGCACCTGCAAATTCATCAACATTAACCTATGCAACTATTGGACCCTCGCCTTTGGATACCGCCGCCGCCGCTGCAGCTTCCGCGGCCGCTTCTACGGCTCGCAGTATGGCAGCTGATTTCAGCTTCTACAATCACTTGGCTTCGAATGCTGTGACACGCACCGCAGTTCGAGAGGACATTCTGACTGTTATGCTTGCCAAGCTTGAAACTCTAACTGCTCAGCTGGAAGAGCTATCGCAAAAGGTTGAGGAATTAGCTGATGCTACTACCCATACCCCAGCCCAACCTGTAACCCAATAAAGAAAAAACTTAAATTGAGATGGTGTTATGAATCTTTATTGATACTTGTTTTTTCTGACATGGTAAGCTCTTGACCACCGTTCCCTATCATTAAGAACACGGTGAATGTGTTCCAGTATTTTGTAAAGATGAGCCTGTATATTAAGGTACATTGGCATTAGGCCATCTTTGGGATGAAGGTAGGACCATTGAAGGGCTTCATGTTCCGGGTTAGTGTTGTAGATAATCCAGTCATAGCAACAACGCTGGGCATGGTGATTAAATATATCTTTTAACAACAAGCTAATTGCTAATGGAAGACCTTTAGTATAGGTATTGATAAAACGGTTAAGCTGGGTGGGATGCATCCGAGGTGACATGATATGAAGTTTTGATTGTATTTTGAGATTGGCAATGTTACCTGCCAAATCTCTTCTTGGATTCATATTGTGGAGAACCACGAAAACGGTGTAGCCAGTACACTTGGGAAATTTGTCATGGAGTTTAGAAGGAAAGGCATGGAAAAACTTGGAAACGCCTTTGTGACTTCCCAAATTTTCCATACACTCATCCATTATTATGGCAATTGGACCGCGAGCAGCGGCTTGAGCAAAAATGTTTTCTGGATCAGAAACATCATAGTTGTGGTCTAGAGTTAGGTCATCGTAGGACAACTTAACAAATTTAGGACACAGCGTTCCAGATTGTGGAATAATAGTTCCCTCTGGTCCTGGGACATAATTTCCCTCACAAATTTGCATTTCCCAAGATTTAATTTCAGATGGGGGAATCATGTCCACTTGCGGAACAATAAAAAAAACAGTTTCTGGAGCAGGTGTAACCAGCTGGGCAGAAAGCAAATTACGCAACAACTGAGACTTCCCACAGCCAGTGGGTCCATAAATTACCCCAATTACAGGTTGCAAGTGATAGTTTAACGAGGTGCAGCTGCCGTCTTCGTGGAGAAGCGGAGCCACTTCATTCATCATTTGTCGGACGCGGATGTTTTGCTTGGCCAGTTCCCCTAACAGACGCTCTCCGCCTAAGGAAAGTAACTCTTGTAAAGATTTGAAATTTTTAAGTGGCTTTAGGCCATCGGCCATAGGCATGTGGTCCAGGGTTTGCTTCAGCAGTTGCAAGCGATCCCATAGCTCAGTTATATTTTCTATGCCATCTCGATCCAGCAAACTTCCTCGTTGCGGGGGTTTGGCTGGCTGTTGCTGTAAGGAACGAGGCGGTGAGCATCCAAATGGACGAGGGTTTTGTCCTTCCAGGGACGTAATGTGCGCGTCAGGGTTGTTTCGGTCACGGTGAATGGATGCGCTCCTGGTTGAGCGCTGGCCAGTGTGCGCTTTAAACTGAGGCGGCTGGTGCTGAAGCGCGTGTCTTCTCCCTGTGCTTCGGCAAGGTAGCATTTTAACATAAGATCATAAGACAAAGCCTCTGTAGCGTGGCCTTTAGCCCGTATTTTTCCTTTGGAGGTGCTCCCGCAGTGAGGACACTGAAGGCATTTAAGGGCGTACAGTTTTGGAGCCAAAAAAACAGATTCTGGAGAATAAGCATCTGCGCCACAATAACTACAAACAGTTTCACATTCAACTGACCAGGTCAGCTCAGGACATGATGGATCAAAAACAAGTTTCCCTCCGTACTTTTTGATGCGTTTCTTACCTTGCGACTCCATAAGGCGGCGTCCTTTCTCTGTGACAAAAAGACTGTCAGTGTCTCCGTATACAGATTTAAGGGGTCTATCCTTCAGTGGTATTCCGCGGTCCTCCTCGTACAGGAATTCTGACCACTCTGACACAAAAGCTCTAGTCCAAGCAAGTACAAAGGAAGCCACATGGGAAGGGTACCGATCGTTGTTAATTAAAGGGTTAGAACTTTCTAAGGTGTGTAAACACATGTCTCCTTCTTCAGCGTCCATGAATGTGATTGGTTTGTAGGTGTAAGTCACGTGTTCACAATTTTCTGGTGGTGGGCTATAAAAAGGGGCGGGTCCTTGGTCTTCATCGCTTTCTTCTGCTTCGCTGTTTACGAGCGCCAACTGGTTGGGTGAGTACACGCGCTCAAAGGCAGGCATTACCTCTGTACTCAACGTGTCAGTTTCTATAAACGATGAGGATTTGATGTTTAATCGCCCCGCTGCAATTTCTTTCATTAGGCTTTCTTCCATTTGATCAGAAAAAACTATTTTTTTGTTATCTAGTTTGGTAGCAAAAGATCCGTACAAGGCATTGGAAAGCAGCTTGGCTATAGATCTTAGGGTTTGATTTTTGTCCCTATCGGCCCGTTCTTTTGCGGCAATATTGAGTTGCACATATTCGCGTGCCAGGCATTTCCAGGTGGGGAAAATGGTGGTGCGCTCGTCAGATAGCAAGCGTAAGCGCCACCCGCGATTATGCAGTGTAACCAGATCTACGCTGGTAACTACTTCACCGCGCAAGCTTTCATTGGTCCAGGCTAAACGACCGCCTTTTCTAGAACAAAAAGGAGGAAGAACATCCAACTGATTTTCATCTGGGGGGTCGGCATCTATAGTAAAAATGCCAGGACAAAGATTTTTGTCAAAATAATCAATTTTGCAAGTGTAATTTTCCAGCGCCACCTGCCATTGCCGCACGGCCAATGCCCGCTCATAGGGGTTAAGGGGAGGACCCCAAGGCATGGGGTGTGTGAGGGCCGATGCATACATGCCGCAAATATCATATACATATATGGGCTCTTTTAGTACTCCTATGTAAGTAGGATAGCACCTGCCGCCACGAATGCTGGCGCGAACGTAGTCATATAGCTCATGTGAAGGCGCCAGGATGTTGGGCCCAAGATGTGTGCGCTGTGGTTTTTCGGCGCGGTACAAAATTTGTCTGAAAATTGCATGAGAGTTAGAGGAAATGGTAGGACGCTGAAACACATTAAAATGTGCCGCGTCAAGACCCACTGCGTCAGTAACAAACTGGGCGTATGAGCTACGCAGTTTTTCTACCAATGAGGCAGTCACAAGTACATCCAGGGCACAATAGTTTAATGTTTCCCCGATAAGATTGTAATTTTTTTCTCCTTTTTTTTTCCATAGTTCTTGATTTAGGAGGTATTCCTCCTTATCCTTCCAGTACTCCTCCAGGGGAAACCCATTTGCATCTGCACGGTAAGAACCAAGCATATAAAACTGATTTACCGCCTTGTACGGACAACATCCTTTTTCTACAGGCAGGGCATACGCTTGTGCAGCCTTTCTTAAAGATGTATGAGTAAGAGCAAAGGTATCTCTGACCATTACTTTTAAATACTGGTATTTAAAATCTTGGTCGTCACACCCTCCGTGTTCCCACAGTAGGAAGTTAGTTCGCTTTTTGTAGTGGGGATTGGGAAGGGCAAAAGTAATATCATTAAATAATATTTTGCCAGCTCTTGGAATAAAATTTCTAGAAATTTTAAAGGGTCCAGGGACGTCCAAGCGGTTATTGATTACCTGAGCGGCAAGAACAATTTCATCAAATCCATTAATATTGTGTCCTACTATATACAACTCTACAAATCTTGGCTCACCCTTAATTGCAGGGGCTCTTTTAAGATCTTCGTAGGAAAGATCTTCAAGCGCGACTAGTCCGTTTTCTTCTTGAGCCCATTGAGACAAGTGTGGATTTTTTTGTAAAAAAGTCATCCAAAGATCAGTAGCTAAGGAGGTTTGTAAGCGGTTTCTATAGGTACGAAACTGTTGACCGACCTTCATTTTTTCTGGGGTTAAGCAGTAGAAAGTAGTAGAGTCTTTTTCCCATTGGTCCCATCCAAGTTCTAATGCAAGTTGTAAGGCATGTTTGACAAGATTGTCATCCCCAGACAGTTTCATCACCAGCATAAATGGGACAAGTTGCTTTCCAAATGCCCCCATCCAGGTGTAGGTTTCTACATCATAGGTAATAAAAAGGCGCTCAGTGCGAGGATGCGAACCGATTGGGAAAAAGTGGATCTCCTGCCACCAGTTGGAAGAATGGCTGTTGATGTGATGAAAGTAGAAATCTCGTCGGCGGACAGAGCATTCATGCTGATGTTTGTAAAAGCGTGCGCAGTGTTCGCATCGTTGCACGGGCTGTATCTGTTGAATGAGGTGTACCTGGCGGCCTCGCACCAGAAAGCAGATGGGAAAATCAATACCACTTGGCAGCTGCCGTTCGTCCTCTTCCTCTTCTGCTGCATTGCCACTACCGTTTGGATCCTCGAAAGCGAGAACGGAGAGGGTGACGGTGCCCCTCGAGCTGCATGTCCAGATTTCAGCACGAGAGGGGCGGAAACGGGAAATCAGGGCGTACAGCCTGGAGCTGTCCATGGTATCAGTCAGAGAGAAAAGCATGTCCGCGGGGACAGCGCGCAAGTTGACTTCGCACAGGCGGGTAAGAGCAGGCTGGAGGTGCAGGTAATACTTAATTTCTAGAGGCGTGCCGTTGGCAGAGTCTATTGCGTGAAGTATTCCATGAGCCCGGGGACTAACCACGGTTCCACGGTGCACTTTTCCAATGCGCCTGCTTAAAATCGGCGGCGCGGACGAGCTCCCGGAGGAAGCGGCGGTTCGGGTCCTGCGGGAAGCGGGGGAAGCGGTATGTCGGCCTGACGCTCTGGCAGGGGAAGGTGTTGAGCCCGAAGTTGACTGGCATGGGCGACTACCCGGCGATTGATATCTTGAATCTGTCGGCGTTGTGTAAACACTACCGGCCCTGTTGTTTTGAACCTGAAAGAAAGTTCAACAGAATCAATCTCAGTGTCATTTACTGCAGCCTGTCTTAAAATCTCCTGAACGTCGCCTGAGTTATCTTGGTAGGCAATTTCTGCCATTAATTGATCAATTTCTTCCTCCTGGAGGTCTCCATGTCCCGCACGTTCAATAGTGGCTGCAAGGTCATTAGATATCCGACTCATAAGCTGTGAAAATGCGTTTAGTCCAATTTCGTTCCAGACTCGGCTGTATACTACCCCTCCTTCGCTGTCCCGAGCGCGCATAACCACTTGCGCCAAGTTGAGTTCCACGAGCCGTGCGAACACGCCGTAGTTGCGCAAGCGCTGAAACAGGTAGTTTAAGGTGGTGGCAACGTGTTCTGAGACGAAGAAATACAGAATCCACCGACGAAGCGTCAGCTCGTTGATGTCACCTAAGGCTTCAAGACGTTCCATGGCTTCGTAAAAGTCTACTGCAAAATTGAAAAACTGGGAGTTGCGAGCTGCCACCGTCAATTCTTCTTCCAACAGACGAATAAGCTCGGCCACCGTCTCGCGCACTTCTTGCTGAAATGCGCCCGGAACTATTTCTTGTTCTTCCTCTTCTACCTCCATTATTTCTTCCTCGACCACAGGTGGTGGGGGTTGTCTTCTTCGACGCCGGCGAACGGGCAGCCTGTCTACAAATCTTTCAATCATTTCGCCGCGACGGCGGCGCATAGTTTCGGTTACTGCTCGACCGTTTTCACGTGGTCGTAACTCAAAAACTCCACCTCTAAGTTCTGTTTCATGTAAAATGGGAAATGAGGCGTTGCGAGGGGCGTTAGGTAGGGATACAGCGCTGATTATGCATTTTATTATTTGCTGCGTAGGAACTCCGCGCAAGGAGCTAAGCGTCTGCATATCCACCGGGTCGGAGAACCTTTCAAGAAAGGCATCTAGCCAGTCACAGTCACAAGGTAGGCTAAGTTTTGTTTCTTCTAAAGTACCAGGAAGCTGAGCAATGCTACTAATAATGTAATTGAAGTAAGCTGTTTTAAGCCCACGAATGGTTTTAAGAAGCACCACATCTTTGGGTCCGGCTTGTTGAATTCGCAGGCGGTCTGCCATTCCCCACACGTCACTTTGACATCGTCCAAGATCTTTGTAGTAGTCTTGCATTAACCTTTCCACCTCTACCTCGCGGTTTCCGCGATCAGCCATGTGCGTGCTTCCGTAGCCTTGCAGCGGTTGTAATAAAGCTAAATCTGCCACTACCCGTTCCGCAAGCACTGCCTGTTGAATTTGGGTAAGGGTGGTTGCAAAGTCATCCACATCTACAAAGCGGTGATAAGCTCCTGCATTAATGGTGTAGCTGCAGTTTGTCATTACTGACCAATTAACAGTTTGCGTGCCTGGCTGTACAGTTTCTGTGTATCGCAAGCGTGAGTAAGCCCGAGAGTCAAAAACATAGTCATTGCAGGTGCGCACTAGGTATTGATAGCCCACAAGGAAATGAGGAGGAGGTTCGCGATACAACGGCCAGCCAAGCGTAGCCGCAGCACCTGGAGCGAGATCTTCCAACATGAGGCGGTGGTATTCATATATGTATCTGGACATCCATGTGATGCCGGCAGCGGTAGTTGTTGCTCGCATAAATTCGCGGGCTCGGTTCCAAATATTGCGCAGGGGTAAAAAGCGTTCAATAGTTGCCACGCTTTGACCGGTCAGGCGTGCGCAGTCTTGAATGCTCTGGACATGGAAAAAATGAAAGTCGGTAAGCGACTCCCTTCCGTGGTTTGGTGGAAAAGTCACAAGGGTACCATAGCGAGGAACCCCGGTTCGAAACCGGCAGGATCCGCTATGAGCACAAGTGAGGCGCTTGCGCGTTGAACCCGGCCAAGGACCCCCAGACACGGAGAGGAGTCTTTTTTTATTTATTTTTTCTTAGATGCATCCTGTCCTGCGACAAATGCGACCTCAGCCCAGGGCAACCACGGCCTCAGCAGCGGTGGCGCTTTCGGGCTCTGGCGAACAGGAAGAGCCTCAATGTCCTACATTGGAGTTGGAAGAAGGAGAAGGCATAGCCCGATTGGGCGCCCACTCTCCTGAGCGTCACCCAAGGGTGCAGCTCGCCCGGGACAGTCGCGTGGCATTTGTGCCTCGTCAGAACATGTTTCGCGACAACAGCGGGGAGGAAGCTGAGGAAATGCGAGACTGCAGGTTTAGGGCCGGTCGCGAGCTGCGCCGCGGATTTAATCGCGAGCGACTGCTGCGTGAGGAGGACTTTGAGCCAGATGAACATTCGGGGATTAGTTCTGCACGGGCCCATGTATCAGCAGCCAACTTAGTAACAGCATATGAACAAACGGTTACAGAGGAACGTAACTTTCAAAAAAGCTTTAATAACCATGTGCGCACACTAATAGCGCGAGAAGAAGTAGCCATTGGTTTAATGCATCTTTGGGACTTTGTAGAAGCTTATGTACATAATCCAGCAAGTAAACCCCTAACTGCCCAGCTGTTCTTAATAGTTCAACATAGTAGAGACAATGAAACTTTTAGGGATGCAATGCTTAACATAGCTGAACCCCAGGGTCGGTGGTTACTCGATTTAATTAACATTCTGCAGAGCATTGTGGTTCAGGAACGCAGTCTTAGTTTGGCAGACAAGGTGGCCGCCATTAATTACTCCATGTTAAGTTTGGGAAAGTTTTATGCTCGTAAAATCTACAAAAGTCCGTATGTTCCCATTGACAAGGAAGTGAAGATAGACAGCTTTTATATGCGCATGGCTTTAAAGGTACTAACATTAAGCGACGATCTTGGAGTGTACCGCAATGACCGAATCCACAAAGCAGTAAGCGCCAGTCGCCGCAGAGAGCTAAGCGACAAAGAGCTTATGCATAGCTTACAAAGGGCGCTGACGGGAGCAGGAACAGAGGACGAGTCGTTCTTTGATATGGGCGCAGACCTACGGTGGCAGCCAAGCGCTCGCGCTTTGGAGGCAGCTGGAGTGGCGTCTGCTGACGTCACTGGCGATGACGATGACGAAGACCAGTACGAGGACTGATCGGCCGTACCTTTTGTTAGATGCAGCGACCGGCGATCATCGCGGAGAGGGCTCCTAACCTGGATCCCGCGGTTTTGGCGGCCATGCAAAGCCAGCCTTCTGGCGTTACAGCTTCAGATGACTGGACAGCGGCCATGGATCGTATTATGGCTTTAACGGCGCGCAGTCCTGATGCTTTTCGCCAGCAGCCCCAAGCTAACCGCTTTTCGGCCATTTTGGAAGCAGTAGTGCCGTCTCGTACTAACCCTACTCACGAGAAAGTGTTAACCATTGTAAATGCTTTGTTGGATAGCAAAGCCATCCGCAAAGATGAGGCTGGTTTAATATACAACGCTTTGCTTGAGCGCGTGGCACGCTATAACAGTACCAATGTGCAGGCTAACTTAGACCGGATGGGTACAGATGTAAAGGAGGCGCTGGCTCAACGAGAGCGCTTTCATCGCGATGGTAATCTTGGTTCGCTAGTAGCATTAAACGCTTTTTTGAGTACTCAGCCGGCTAATGTTCCGCGTGGTCAGGAAGATTATACAAACTTCATCAGCGCCTTGCGACTAATGGTTACTGAAGTGCCTCAAAGTGAAGTGTATCAGTCTGGACCCGATTACTTTTTTCAAACGTCCAGGCAGGGTTTGCAAACCGTAAACTTAACTCAGGCTTTTAAAAATTTGCAAGGTTTGTGGGGGGTTCGTGCTCCAGTAGGCGATCGTTCAACTTTGTCCAGTTTACTAACACCAAACTCGCGCCTATTACTGTTGCTAATTGCCCCCTTTACCAACACCAACAGTTTAAGTCGAGATTCATACCTGGGTCACTTAGTTACTTTGTACCGCGAAGCCATTGGTCAAGCGCAGGTAGACGAACAAACTTATCAAGAAATAACCAGTGTTAGTCGCGCACTGGGCCAGGAGGACACTGGCAGTTTAGAGGCCACACTTAACTTTTTACTAACTAACCGTCGCCAGCAAGTGCCTCCTCAGTACACTTTAAATGCGGAAGAAGAACGCATATTGCGCTATGTACAGCAATCTGTAAGTTTGTATCTTATGCGTGAGGGTGCCACCCCCAGTGCCGCCTTAGACATGACAGCGCGCAATATGGAGCCGTCCTTCTACGCTTCCAATCGAGCTTTCATTAATCGCTTGATGGATTACCTTCACCGCGCTGCGGCCATGAACGGGGAATACTTTACAAATGCAATTCTAAATCCGCATTGGTTGCCCCCTCCTGGATTTTACACTGGTGAATTTGATTTGCCGGAAGGAAATGATGGCTTTTTGTGGGATGATGTTACGGACAGTCTGTTTAGTCCTGCAGTTATTGGACACCATGGTAAAAAGGAAGCAGGTGATGAAGGTCCCTTGCTTGACTCTCGGGCGAGTTCTCCATTCCCCAGTTTAACTAGTTTACCCGCCAGTGTTAACAGCGGTCGTACCACCAGACCCCGACTAACAGGTGAAAGTGAATACTTAAATGACCCCATCTTGTTTCCAGTGCGCGACAAAAATTTTCCCAACAATGGCATAGAAAGTTTGGTAGATAAAATGTCTCGCTGGAAAACATATGCACAAGAGCGGCGAGAATGGGAGGAAAGACAGCCAAGACCAGTTCGCCCTCCTAGGCAACGTTGGCAGCGACGCAAAAAAGGGGCACATGCGGGGGATGAAGGAAGCGATGACTCAGCTGACGACAGTAGTGTATTAGATTTAGGAGGGTCAGGAAACCCATTTGCTCATTTGCGCCCACAGGGTTGCATAGGGTCATTGTATTAAATTGAATAAAAGCATACTTACCAAAGCCATGGCGACCAGTGTTCGTCTTATTTTCCTTCTTCCGTTAGCTGTGAAATGAGGCGCGCGGTGGAACTGCAGACAGTGGCTTTTCCTGAGACACCACCTCCCTCTTACGAAACCGTGATGGCAGCGGCGCCACCCTACGTGCCTCCCCGCTATTTGGGTCCTACGGAGGGAAGAAACAGTATCCGTTACTCGGAATTGTCACCGTTGTACGATACCACTCGAGTGTACTTGGTGGACAACAAGTCTTCTGACATTGCTTCATTGAATTACCAGAATGATCACAGCAACTTTTTAACCACTGTAGTGCAAAATAATGACTATTCCCCTATAGAGGCTGGCACGCAAACTATTAACTTTGATGAAAGGTCTAGATGGGGTGGAGATTTAAAAACCATCTTACATACCAACATGCCAAACGTGAACGATTTTATGTTTACCACCAAATTTAAGGCCAGGGTAATGGTGGCTAGGAAAACAAACAACGAAGGCCAAACCATTTTAGAATATGAGTGGGCAGAATTTGTGCTACCCGAGGGTAACTATTCGGAAACCATGACTATTGACTTAATGAACAATGCTATTATTGAGCATTATTTGCGAGTAGGAAGACAGCATGGAGTGCTGGAAAGTGACATTGGAGTTAAGTTTGACACCAGAAACTTTCGTCTGGGTTGGGACCCCGAAACCCAATTAGTAACTCCGGGAGTGTACACTAATGAGGCTTTTCATCCAGATATAGTACTGCTTCCAGGTTGCGGGGTTGATTTTACAGAGAGCAGATTAAGCAACATACTAGGTATAAGAAAGAGGCAGCCGTTTCAGGAAGGATTTGTGATTATGTATGAACACTTAGAGGGAGGCAATATTCCAGCTCTTTTGGATGTAAAAAAATACGAAAACAGTCTGCAGGATCAAAACACTGTAAGAGGAGACAACTTTATTGCCTTAAATAAGGCTGCTAGGATTGAACCGGTTGAAACAGACCCCAAAGGACGCAGTTACAACTTGCTTCCAGACAAAAAAAATACTAAATATCGCAGCTGGTATTTGGCATACAACTACGGAGACCCAGAAAAAGGAGTTCGGTCATGGACTCTACTAACAACTCCAGATGTAACAGGCGGCTCCGAACAGGTGTACTGGTCCCTACCCGATATGATGCAAGATCCGGTGACTTTTCGCTCCTCGCGTCAAGTTAGCAACTATCCTGTAGTTGCAGCAGAATTACTGCCAGTTCATGCTAAAAGCTTCTACAACGAGCAAGCCGTCTACTCACAGCTTATTCGCCAGTCAACCGCGCTTACGCGCGTGTTTAATCGCTTTCCCGAGAACCAGATACTGGTGCGTCCACCAGCCGCTACCATCACTACCGTCAGTGAAAACGTTCCCGCCCTTACAGATCACGGGACCCTGCCGCTGCGTAGCAGTATCAGTGGAGTTCAGCGAGTCACCATCACTGACGCCCGCCGCCGGACCTGTCCCTACGTTTACAAAGCACTGGGCATAGTTTCTCCACGAGTGCTTTCTAGTCGCACTTTTTAAAAAAGTGTGGTAACATGTCCATTTTGGTTTCGCCAAGTAACAACACGGGCTGGGGACTGGGTGCCGCCCGCATGTATGGAGGAGCTAAAACAAGGTCTAGCCAACATCCAGTGCGCGTACGCGGACATTACCGAGCTCCATGGGGCGCGCATACCCGAGGACGCACTGGTCGCACCACTGTAGACGATGTTATTGACTCGGTAGTGGCCGATGCTCGCAAGTACCGCGCGCCCGCTGAAACAGCAGGGTCTACTGTTGATGCAGTAATTGATGAGGTAGTGGCAAACGCGCGGGCTTATGCAAGGCGCCGCAGACGGCTGCGTCGCCGGCGTAGACCAACCACCGCCATGCGCGCGGCCAGAGCGTTGGTTCGACGGGCCAGGCGCATTGGGCGGCGAGCTATGATGCGGGCAGCCAGGCGGGCTGCAACGCCTGCCGGTCGAGCGCGGAGACGGGCCGCAGCTGCGGCCGCAACAGCTATTGCAAACCTAGCTGCTCCGCGACGAGGAAATGTATACTGGGTGCGCGACTCAGTGACCGGGACGCGTGTGCCAGTTCGTACGCGTCCACCTCACCCTTAGAAGACAAAGAGTGACTCAATGTCTGTTATGTATGCCCAGCATGACCAAACGCAAGTTCAAAGAAGAGCTGCTGCAGGCCTTAGCGCCTGAAATATATGGCCCATCGGATAACCTTACCAAGCGCGATATCAAGCATGTTAAAAAACGGGAAAAAAAAGAGGAAGAAGTCGCCGCGGCGTCAGCAGACGGCGTCGAGTTTGTGCGCTCATTTGCGCCCAGACGTAGGGTACAGTGGAAGGGACGGCAAGTAAAACGCATTTTGCGACCGGGCACCACAGTGGTTTTTTCTCCCGGAGAGCGAACGATTATGCGTCCCCTAAAGCGCGAGTACGACGAAGTGTACGCAGACGATGACATTTTGGAGCAAGCGGCACAACAGACTGGGGAATTTGCATATGGAAAAAAAGGGCGTTACGGAGACAAAATTGCTATTCCTTTGGACGAGGGAAATCCAACACCCAGTTTAAAGGCTGTCACTTTGCAACAAGTGTTGCCCGTCCTTGGGCCTTCGGAAGAAAAGCGTGGAATTAAAAGGGAAGCCATGGATGAATTGCAGCCTACAATGCAACTGATGGTGCCTAAGCGGCAAAAGTTAGAGGACGTACTAGAGCACATGAAGGTGGATCCTAGCGTACAGCCAGATGTAAAAGTACGTCCGATAAAAAAGGTAGCTCCAGGATTGGGAGTTCAAACAGTGGACATTCAAATTCCTGTGCAAACTGCATTGGGTGAAACTATGGAAATCCAAACTTCGCCAATAAAAACAACGGTGAACGCAAGCGTGCAAACAGACCCTTGGTACCCGCCAGTGCTTTCAACAAAAAAAAAGCGTCACTACAGACAAACAAGTTCGCTTTTGCCAGACTACGTTTTACATCCTTCCATTGTGCCCACGCCTGGGTACCGTGGGACAACTTTTCAGCGCCGAGCCACAGCCCCTAGCCGTAGACGAGGTCCATCACGCCGTAGACGTCGACGCAAAGCCACTTTAGCCCCAGCGGCAGTACGTCGCGTTGTACAAAGGGGGCGCACACTAATACTTCCATCCGTGCGTTACCACCCTAGCATTCTCTAACAAGCTGCGCTGCCGTTTTTTCAGATGGCTCTTACTTGCCGAATGCGCATACCCATTCCAGGATACAGAGGACGACCCCGCCGGAGGAAAGGGCTGACCGGGAACGGTCGATTTCGGCGGCGTAGTATGCGCAGACGCATGAAGGGTGGGGTGCTGCCCTTCCTAATTCCACTTATTGCTGCGGCCATTGGAGCCGTTCCCGGAATTGCCTCAGTAGCCTTGCAGGCTTCTCGAAAAAATTAAAATAAAATAAAACTTCCAACTTATTACTGGTACTATGACTGTTTTATGCAGACTAAATGGAAGACATCAATTTTTCGTCGCTGGCCCCGCGACACGGCACGCGGCCGTACATGGGCACCTGGAACGAGATCGGCACGAGCCAGCTGAACGGGGGCGCCTTCAATTGGAACAGTATCTGGAGCGGTCTTAAAAATTTTGGTTCCACGATTAAGACATATGGCACCAAGGCGTGGAACAGCCAAACCGGCCAGATGCTAAGGGACAAGTTAAAAGACCAAAATTTTCAACAGAAAGTTGTAGATGGTCTGGCTTCGGGAATTAATGGAGTTGTAGACATAGCCAATCAGGCTGTACAGAAAAAAATTGCCAACCGTTTAGAGCCGCGGCCCGACGAGGTAATGGTAGAGGAAAAGCTGCCACCTCTAGAAACTGTGCCCGGATCCGTTCCAACCAAAGGAGAAAAGCGGCCACGGCCGGATGCAGAGGAAACCTTAGTAACGCACACAACAGAACCGCCGTCCTATGAGGAAGCAATAAAACAAGGAGCCGCTCTGTCACCTACCACCTATCCCATGACCAAGCCTATTTTACCCATGGCTACTAGAGTGTATGGAAAAAACGAAAATGTGCCTATGACCCTTGAGCTGCCTCCTTTGCCAGAACCCACTATCGCGGATCCCGTAGGTTCCGTTCCTGTTGCATCTGTTCCAGTTGCATCGACAGTGAGCCGTCCAGCAGTGCGGCCTGTTGCCGTGGCTAGCTTGCGAAACCCACGATCCAGTAATTGGCAAAGTACCCTAAACAGTATTGTGGGACTGGGAGTAAAGTCTCTCAAACGCCGACGCTGCTACTAACATTAAAAGACGAGTGTTAATTCCCATCTGTGTATACGCCTCCTATGTTAGCGCCAGAGGACCAACGCGTGAATCGCAGTCACCACCAGCGCTTTCAAGATGGCCACTCCCTCGATGATGCCGCAGTGGTCTTACATGCACATCGCCGGTCAGGATGCCTCGGAGTACCTGAGTCCCGGTCTGGTGCAATTCGCCCGCGCCACGGACACCTACTTCACCCTGGGAAACAAGTTTAGAAACCCCACCGTGGCTCCCACCCATGATGTTACCACCGATCGCTCGCAGCGTCTGACGCTGCGTTTTGTGCCCGTGGATCGGGAAGATACTACCTACTCCTACAAGGCTCGCTTTACGCTGGCTGTGGGTGACAACCGCGTGTTAGACATGGCTAGTTCTTACTTTGACATTCGAGGGGTACTGGATCGTGGTCCCAGTTTTAAGCCCTATTCCGGAACCGCCTACAATTCTTTGGCACCAAAAGGCGCTCCTAATGCTTCACAATGGTCAGATAACGCTAAGCTTAATACCTTTGCTCAGGCGCCGTATCTTAGCGACACTATCACCGCCGCCGATGGTATTAAAGTTGGAACAGACACCGCCCAGGCAGGCGCGGCGGTGTATGCCAACAAAACTTATCAGCCAGAGCCGCAAGTAGGACCAAGTGAATGGAACACCAGCATTGAAAACGTTAAAGCTGGCGGGAGGGCATTAAAGCAAACCACTGCAATGCAGCCGTGCTATGGCTCCTACGCTCGTCCAACCAACGAACACGGAGGACAATCCAAGGATGACAACATTGAACTTAAGTTCTTTGATTCAGCTAACAATGCAGCAAACACTGCTCAAGTTGTGTTCTATACCGAAGACGTAAACCTTGAAATGCCAGACACGCATCTTGTGTTTAAGCCTACTGTTACCAATGGAACAATTGCTTCTGAGTCGCTGTTGGGACAGCAAGCAGCGCCAAATAGAGCAAACTACATTGCATTCAGAGATAATTTTATTGGCCTGATGTATTACAACAGTACAGGCAACATGGGTGTATTGGCCGGGCAAGCTTCCCAACTTAACGCAGTAGTAGACCTGCAAGACAGAAATACAGAGCTGTCATACCAGTTAATGCTGGATGCTTTGGGAGACAGAACACGGTACTTTTCCTTGTGGAATTCCGCAGTGGACAGTTACGACCCTGACGTTCGCGTTATTGAGAATCACGGGGTAGAGGATGAACTACCAAATTATTGCTTTCCTCTTAGCGCAGTAGGTGAAATAAAAAATTACAAAGGCATTAAGCCAGATAACGGAGGAGGAGGTGGCTGGACTGCCGACAACACTGTCAGTGAAGCAAACCACATAGGCATTGGGAATATAGCCGCCATGGAAATTAATTTGCAGGCTAATTTGTGGAGAAGCTTCTTGTACTCAAATGTGGGCTTATACCTACCAGACGACTTAAAATACACTCCAGGAAACATAAAACTACCTGATAACAAGAACACCTACGAGTACATGAACGGGCGTGTGACTGCCCCGGGGTTGGTGGATACCTATGTCAATATCGGCGCTCGCTGGTCCCCAGATGTGATGGATAATGTAAACCCTTTTAACCACCACCGAAACGCAGGGTTGCGCTACAGATCCATGTTGCTAGGCAATGGGAGATTTGTTCCTTTTCACATTCAGGTGCCGCAAAAATTTTTTGCCATCAGAAATTTGTTGCTGTTGCCCGGTTCCTACACTTACGAATGGAACTTTAGAAAGGATGTAAACATGATTCTTCAGAGCACACTGGGAAATGATCTTCGGGTGGACGGAGCCAGCGTTCGCTTTGACAACATTGCCCTGTATGCTAACTTTTTTCCCATGGCACATAACACAGCTTCTACTTTAGAAGCCATGTTAAGAAATGACACCAACGACCAGTCTTTTAACGATTATTTGTGTGCTGCAAACATGCTGTATCCCATCCCAGCTAACGCCACCAGCGTGCCCATTTCAATACCTTCGCGAAATTGGGCGGCATTTAGAGGCTGGAGCTTTACTCGCCTAAAAACTAAAGAAACTCCTTCCCTGGGTTCAGGGTTTGACCCCTACTTTGTATACTCTGGAACCATTCCCTATTTAGACGGCACCTTTTACCTAAACCACACTTTTAAGAAGGTGTCAATCATGTTTGACTCCTCCGTGAGTTGGCCTGGAAATGACCGTTTGCTAACCCCAAATGAATTTGAAATAAAGCGTTCTGTGGATGGGGAGGGATACAATGTGGCCCAATGCAATATGACTAAGGATTGGTTCCTAATACAAATGCTTAGTCATTACAACATTGGATACCAAGGTTTTTACATTCCAGAGAGCTACAAGGACCGCATGTATTCTTTCTTTAGAAACTTTCAGCCCATGAGTAGGCAAGTTGTGGATACCACAGAATATAAGAACTACAAAAAAGTAACCGTAGAGTTTCAACATAACAACTCAGGATTCGTGGGATACCTGGGCCCCACTATGCGGGAGGGACAAGCTTACCCCGCCAACTATCCCTACCCTCTTATAGGCAAAACAGCTGTGGAAAGCATCACACAGAAAAAGTTTCTATGCGATCGTGTTATGTGGCGCATCCCATTTTCTAGTAACTTCATGTCTATGGGGGCGCTAACGGATCTTGGGCAAAATATGCTGTACGCAAACTCAGCCCATGCTCTAGACATGACATTTGAGGTGGATCCAATGGATGAGCCTACCCTTCTTTATGTTTTATTTGAAGTTTTCGACGTGGTACGCATTCACCAGCCACACCGCGGCGTCATTGAAGCGGTCTACCTGCGCACGCCCTTCTCGGCGGGTAACGCTACCACCTAAGAAGGCACCCTCCCAGACTGCTGTAATGGGTTCAAGCGAACAGGAGCTGACGGCCATTGTTCGAGATCTAGGCTGTGGACCCTATTTTTTGGGAACCTTTGACAAACGTTTTCCGGGTTTTGTGTCTCGCGACCGCTTATCATGTGCTATTGTTAACACTGCCGGTCGCGAAACTGGGGGCGTACACTGGCTGGCTTTTGGATGGAACCCCAAATCGCACACTTGCTATTTATTCGATCCATTTGGATTTTCTGATCAACGACTAAAACAAATCTATCAGTTTGAGTACGAAAGTCTGTTGCGCCGTAGTGCGCTAGCGGCCACTAAAGACCGATGCGTTACCCTAGAAAAGTCAACCCAAACTGTACAAGGACCGTTTTCTGCAGCGTGCGGCCTGTTTTGTTGTATGTTCTTACACGCTTTTACTCACTGGCCTGACCATCCAATGGATAAAAATCCCACTATGGACCTACTTACTGGGGTGCCTAATTGTATGCTACAAAGTCCTCAGGTAGTGGGCACATTGCAACGCAATCAGAATGAATTGTATAAATTCTTAAACAATCTGTCCCCTTACTTTCGTCACAACCGCGAGCGCATAGAAAAAGCTACATCTTTTACTAAAATGCAAAATGGACTCAAATAAACGTGTACACAATGCATTAATAATAAAACCATTTTATTAGCTCATTGGAGTACAAGCTTGACTGTTTTATTAAAAATCAAATGGCTCTTCGCGACAGTCGCCGTGGTTGGTGGGCAGGGATATGTTTCTGTACTGCAAACGCTGATGCCACTTGAATTCTGGAATAACAAGCCTAGGGGGGGAGCCGTCAAAATTTTCTCCCCACAGCTGGCGCACAAGTTGCAGGGCGCCCATAACATCAGGAGCAGAAATCTTGAAGTCGCAATTAGGGCCAGCATTGCCGCGCGCATTGCGATAAACTGGATTTGCGCACTGAAAAACCAACAAACACGGATACTTAATACTGGCTAACGCTCCAGGGTCGGTTACTTCGTTGATATCAATGTTATCCACATTGCTGAGGTTAAAAGGAGTGATTTTACACAGTTGACGCCCCATCCGTGGCAGGCCATCTTGCTTGTTTAAACATTCGCAGCGCACTGGCATAAGGAGACGTTTTTGCCCATGTCGCATGTGAGGGTAGTCGGCCAGCATAAAAGCTTCAATTTGCCTAAAAGCTATTTGAGCCTTCATTCCTTCAGAATAAAACAAGCCGCAGGACTTTCCGGAGAAAGAATTATTCCCGCAGCCAACATCATGAAAACAGCAGCGGGCATCGTCGTTTTTAATTTGAACTACATTACGCCCCCAGCGGTTTTGCGCCACCTTGGCTTTCGAGGGGTTCTCTTTCAACGCTCGTTGCCCACTTTCGCTGGTTACATCCATTTCCACCAAATGCTCTTTGCGCACCATCTCCATTCCATGCAGGCATCTAAGCTCCCCTTCGCGCTCGGTACACTTATGCTCCCACACGCAGCAACCGGTGGGTTCCCAGGAATTCTGTTGGACACCGGCATAAGCTTGCATATATCCTTGCAAAAAGCGTCCCATGAGCTCCTGAAAGGTTTTTTGGGATGAAAAAGTCAGCTGCAAACCGCGCTTTTCTTCGTTGAGCCATGTTGTGCATATTTTCTTGTACACGCTGCCCTGATCCGGCAAAAAACGAAAGGTGGCGCGCTCGTCGTGATCCACATGGTACTTTTCCATTAGCATAGCCATGGCTTCCATGCCTTTTTCCCAAGCTGAAACTAGGGGCTGGCTTGCCGGATTGCGAACAACAACAACATTCTTTTCATTTTCGTCGCTGTTTTGAGCGGAAGCCTTCAAAACGTGTACCTGCCTGGTTTCCATTTTTTGAAAAGACTGAGAACCGTCTGCATGATGCATAATGCGGACGGGCGGCATGCTGAAACCCATTACTCCTAAAACTGCTCTTGGTGGTTCTGCCTCTTCTTCTTCTGCACTCTCTGGGGAAAGAGGTATCGCAGCCATAGATTTCTTGACTTTTTTCTTTGGAGGTAAAGGCACAGCTTCCAGTTCTTCTTCGCTTTCGGAATCCAGAAAGTATCTGCCCATTTTTGGCGGCGGCGGCTGAGCGCTGCGGTCTGGGGTGCGCTCCCTCTGTGAGTGCTGATTGCTGGCCATTATTTAATCCTAGGCAAAGAAACACATGATGGATCTGGAGCCACAGGAAAGCTTAACCGCCCCCACCGCTCCCGCCATTGGCGCTACGGCTGTCATGGAGAAGGACAAAAGTCTACTCATACCCCAAGACGCACCGGTTGAGCAGAACTTGGGCTACGAGACTCCCCCCGAGGAATTTGAAGGCTTTCTTCAAATCCAAAAGCAACCAAATGAGCAAAACGCTGGGCTCGAGGACCATGACTACCTAAACGAGGGAGATGTCCTGTTTAAACATCTACAGCGACAAAGCACTATCGTTCGCGACGCCATATCTGATCGCTCTTCAATACCAGTTTCAATTGCAGAACTATCTTGCATCTACGAACGCAACCTGTTCTCCCCACGTGTGCCCCCTAAACGGCAAGCCAACGGCACATGCGAGCCAAATCCTCGCCTTAACTTCTACCCAGTTTTTGCAGTGCCAGAAGCACTGGCAACATACCATATTTTCTTTAAAAATCACAAAATACCCCTATCCTGTCGAGCTAACCGCAGCCGCGCAGATGAGCTTCTTGCTTTAAGGGCTGGCGCTTCCATACCTGGGATTGTGTCCTTGGAAGAGGTGCCTAAAATTTTTGAAGGTTTAGGTCGGGATGAAAAACGAGCAGCAAATGCCCTGCAAAAAGAAAATGAACAAAATCACCATGGGAATAGTGCTCTAATAGAACTGGAAGGTGACAATGCCCGCCTGGCAGTTTTAAAGCGCAATATTGAGGTTACTCACTTTGCCTACCCGGCAGTAAATCTTCCGCCAAAGGTAATGAGCGCAGTGATGAATCAGCTACTAATTAAGCGAGCCCAACCCATTGACAAAGATGCAAACTTGCAAGACCCGGAGGCAACAGATGATGGAAAGCCGGTTGTAAGCGACGAGCAATTAACTAAGTGGTTGGGAACAGACAATTCCAACGAACTACAACAGCGGCGTAAACTCATGATGGCCGCCGTACTTGTAACTGTGGAACTCGAGTGCATGCATCGTTTTTTCTCCGACATCACCACATTGCGCAAAATTGAGGAATGTCTTCACTACACTTTCCGCCATGGCTACGTGCGCCAAGCCTGTAAAATTTCTAATGTGGAGCTGAGCAATCTAGTTTCTTACATGGGCATCTTGCATGAAAACCGATTGGGACAGAACGTGCTACACTCAACACTACGCGATGAAGCACGCAGAGATTACGTGCGAGACTGCATTTACCTTTTCCTGTTACATACCTGGCAAACTGGGATGGGTGTTTGGCAGCAATGCTTGGAAGAAAAAAACCTTCGAGAACTAAACAAACTGTTAGACAGAGCACTAAAATCCCTATGGACCGGTTTTGACGAACGGACAGTAGCTGCAGAGCTAGCTGACATAATTTTCCCAGAAAGGTTAATGATAACCTTGCAAAACGGCTTGCCTGACTTTATGAGTCAAAGTATGCTGCACAATTATCGCTCTTTTATATTAGAGCGTTCTGGGATGCTTCCTAGCATGTGTTGTGCACTTCCTTCAGATTTTGTGCCTATATATTTTAGAGAGTGCCCCCCTCCCCTGTGGAGCCACTGCTACTTACTACGACTTGCTAACTACCTAGCTTACCACTCAGACCTTATGACAGATTCAAGCGGCGAAGGCCTAATGGAGTGTCACTGCCGCTGCAATCTTTGCACCCCCCACCGTTCTTTGGTTTGCAATACTGAACTATTAAGTGAAAGTCAAGTCATTGGTACCTTCGAAATGCAGGGACCGCAGTCTGACAGCAATTTCACGACGAACCTAAGACTTACCCCTGGGCTTTGGACTTCTGCCTACCTGCGCAAATTTGAACCCCAAGATTACCACGCCCACAGTATCAATTTTTACGAAGACCAATCCAAACCCCCAAAAGCGCCACTAACGGCTTGCGTCATTACGCAGGGAAAAATTCTAGCCCAATTGCATGCTATTAAGCAAGCGCGCGAAGAGTTTTTACTTAAAAAAGGACACGGAGTGTACCTTGATCCCCAAACCGGCGAGGAACTAAACCTTCCATCACCTTTGTGTGCTACTGCGTCTCCCCATTCGCAGCATGTCCCCGAAAGCCGCAAAACAGGCTATTGCGCAGCAACGCTCAAAGAAACAGCAGCAACGGCAGGAAATCTGGGAGGAAGAATCTTGGGAGAGTCAGGCAGAGGACGAGGTCGAGGACTTGGAAGAATGGGAGGAGGAGGAGGCGGACAGCCTAGACGAGGATCCAGAGGAGGAGGAGGAAGGTTCCAAGGACGGAGCGACCGCCGCAAAACCGTCGCTTTCAACCAAGCCCTCTCCAATGAAACCCGCTGTGAGCAAATCTCAGAAAGCCAGCCGTAGATGGGACACCATTGAAACCAGCGCCGCAAACTTGGGTAAGAATCGCAAGCAGGCGCGTCGGGGCTACTGCTCATGGCGGGCTCACAAAAGTAATATTGTAGCCTGCTTGCAGCACTGCGGGGGGAATATCTCATTTGCAAGGCGGTATTTGCTATACCATGATGGAGTGGCGATTCCAAGGAATGTCCTCCATTACTACCGTCATCTCTACAGCCCCTTTGAAGAGCTCGACAAGGAACCGACCTGCAACAGCCAAGCGGCCCACTAGAATCGGCAACAGCAGCAACAAGGAAAGTCCTGAGGCGCGCGAGTTAAGAAAACGCATTTTTCCCACTTTATATGCTATTTTTCAGCAGAGTCGAGGTCAAGAACACGAACTGAAAATAAAAAACCGTTCCCTGCGTTCACTTACCCGCAGCTGTCTCTACCTCAAAAGCGAAGATCAGTTGCAACGCACCTTGCAGGACGCAGAAGCTCTGTTCAATAAATACTGCTCCCTCTCGCTTAAAGAGTAAAAAAAGCCCGCGCGCGGACTTTCAACAGGCGGGAAAAGTGACGTCACAACAAGATGAGTAAAGATATTCCCACGCCTTACATGTGGAGCTTTCAACCGCAAATGGGACTGGCGGCCGGCGCGGCTCAAGACTATTCTAGCAAAATGAATTGGTTAAGCGCCGGACCCCACATGATTTCCAGGGTGAATGGGGTACGAGCCCGGCGTAACCAAATACTGCTAGAACAAGCCGCTCTCACCGCTACACCACGTAATCAACTTAACCCTCCCTCTTGGCCAGCTGCCCTGATATATCAGGAAAATCCCCCTCCTACCACTGTACTTTTGCCTCGCGACGCCCAGGCCGAAGTCCATATGACTAACGCTGGGGCACAGCTTGCGGGCGGTGCACGTCACAGTTTCAGGTATAAAGGTCGCACTGAGCCCTATCCGTCTCCAGCTATAAAAAGAGTACTCATCAGAGGGAAAGGTATTCAGCTGAACGACGAAGTCACATCGCCATTGGGAGTCAGACCCGACGGAGTGTTTCAGCTCGGAGGGTCCGGACGTTCCTCCTTTACCGCTCGTCAAGCCTACCTGACACTACAGAGCTCATCCTCAGCTCCGAGATCTGGTGGTATTGGAACTCTCCAATTTGTGGAGGAATTTACTCCATCTGTTTACTTCAATCCTTTTTCGGGCTCGCCTGGACACTATCCTGACGCCTTCATACCCAACTTTGACGCAGTGAGTGAATCTGTGGATGGCTATGATTAATGTCTAATGGAGCGGCTGACAGAGCGCGGCTGCGACATTTAGACCACTGTCGCCAACCTCACTGCTTTGCTCGAGACATCTGTGTCTTTACCTACTTTGAGCTTCCAGAGGAGCACCCCCAGGGGCCAGCTCACGGTGTCAGAATAACAGTTGAAAAAGGAATTGATACACACCTCATTAAATTTTTCACCAAACGCCCGCTATTGGTGGAAAAAGATCAAGGAAATACTATATTAACTTTATATTGCATTTGTCCTGTTCCCGGATTACATGAAGATTTCTGCTGTCATTTGTGTGCTGAATTTAATCATCTGTAGTGGCGCTGTACCGCCTGAAGAAGAACCTAACTGTCATCCGCATTTAAGCAACATTAAAATCAACCTTTCGATCCCTCATATCACTCTTCGCTGCAGTTTTTTTTCCACACATCTCACCTGGACCTTTAACGGAAAACACGTTACCAATACAGATATAAAGTTTAAACTACACAAAGAAAACATCACTCTATTTCAACCTATTAACCTGGGATACTACCGCTGCTCAGCTCCACCCTGTACGCAAGCATTTTTTGTTGCTCCAGTTATTGACAAACGCCCTGCTCCGACAACAGCTGCTGTCACTGAGCACATCACCGAGGCAGTTTCTCCTTCTAAAGGTACAGAGGAAATTGTGTACTTTTCAAACTTTACAAACCACTTAGTTTTAAATTGTTCCTGTTCTAACTCCTTAATTTCATGGTTTGCTAACAGCTCTCTGTGCAAAACTTTCTACCAAGGAAAACTTTTGTATTCTGCTAAACTCACATTGTGTAACCAGAGCACCCCTTCCCACCTTACTCTATTGCCACCTTTTGTTGCCGGTCGTTACTTTTGCATAGGAGCTGCACGTACTAGCCCCTGTCAACAGCATTGGAATTTAACTTACTGTCCCCCACCAGTGTCGCCCTTTGTGATCAATACTGAATATTTAGACTATAATCCCTTGCTTGCTTACGGCGGTCTCGCAGCTCTTATTTTATTCCTGATTTCTAACTTGTTTCTAGTGCAACATTTGTATTCATACTAACAATGCTTTCCATTTTTCTTTTATTTCTCTTTTCTTTACCTTCTGGCTTGTATGCTCAAACAGCCGAAAGACCACTAAAAGTCGTGGTGGAAGCTGGCCATAATGTAACCCTTCCCCACCTTTCTGGTTCACACCAAACTGGCCATGTTACTTGGCTAGTAGAGACATCAGATTATGGTTCAGCTTCTCCAGACAACTTCATTTTCAGTGGACAAAAACTATGCCAGTTTACTGACAGAACCATGGTGTGGCCTTATTACAATTTACATTTTAACTGTGAAAATTATGACCTTAATCTGTTTTGGCTTAAGGTGGAAAATTCGGCTATTTACAACGTTAAAAATACAGTCAATGCTTCTGAAACAAATATTTACTATGATTTAAGAGTAGTACAAATTTTTCCACCTAAATGCATCATTACTTCAAAGTACCTTACAAACGATTATTGTCACATTACAATTAACTGCACTAACTCTGATTACCCCAATAAGGTTGTGTTTAATAATGTCAGTCGATGGTACTACGGATACGGTAAGGGCAGCCCAACCCTTCCCAACTACTTTATAACTAACTTTAATGTTTCAGGTATTACTAAAAGCTTTAATCACACTTACCCTTTTAATGAGCTCTGTGATTATCCCACATCCCAATCTCAACACAGTTTAACACATACAGTAAGCACAGTAATCTTTTTAGGAATAATTGGCTTCAGCATTTTGATTATTATAGCAGCCTTTATTTATCTGTGCTGGCATAGAAAATCTTTGTGTGTTTCTAAAACAGAACCTCTTATGCCGATTCCTTACTAGTTTTCTTTTTTCTTACAGTATGGTGACGGTTCTTCTCATCTTTTTATGCCTGCCAGTCATTTTTTCTTCTTCGACTTTTGCCGCAGTCAGTGACCTTGATCCCGAGTGTTTAGCCCCCTTTGCGGTGTACCTGATTTTCACATTTGTGACTGCTACCTGCGTCTGCAGTATTATTACTCTGCTAATCACCTCGCTCCAATTTTTTGATTACTACTACGTGAGAATTGTTTACCGCAGACACCACCCCCGTTACCAAAACCCTCAAATTGCGGCTCTTTTGCAGCTCCAACCATGAAAACAGCATTAGTTCTTTTCTTTATGTTAATCCCAGTTTGGGCTAGTTCTTGTCAACTACATAAACCATGGAATTTTTTAGATTGTTATACTAAAGAAACAAACTACATAGGCTGGGTTTATGGAATTATGTCTGGCTTAGTATTTGTCTCCTCTGTAGTTTCTTTACAACTGTATGCGCGCCTTAATTTTAGTTGGAATAAGTATACTGATGATCTTCCCGAATATCCAAACCCCCAGGATGATTTACCCCTAAATATTGTATTTCCAGAGCCCCCGCGTCCTCCTTCTGTTGTTAGCTATTTTAAGTTCACCGGTGAAGATGATTGAACCTGATCTAGAAATTGATGGAAGAATCACCGAACAGAGGCTCCTCACTGATCGCGCTAGGCGACGCCAACAGGATCAAAAAAATAAAGAGTTAATTGATTTACAAACCGTGCATCAGTGTAAAAAAGGACTTTTTTGCCTGGTAAAACAAGCTACCCTTCGCTATGAATCTTTACCAGGCAAAGAACATCAACTGTGCTACACGCTGCCCACTCAGCGACAAACCTTTACTGCAATGGTGGGCTCGGTACCTATTAAAGTGTCCCAACAAGCAGGAGAACAAGAAGGCTCTATTCGGCGCCTATGTGATAACCCTGAATGTTTGTACACTTTAATAAAAACACTGTGCGGTTTAAGAAATCTTTTACCAATGAATTAAATAAATTACTTACCGGAAATCTGAAAATACATCATGGTCTCCGTGTACTCTTATAAAATTTCCCTCTTCCCAACTGTCAAACCTGACAGACTTGCAAACAGCAAACTTTCTCCAAATCTTAAATGGAAGGTCAGATTCTTCTTCCCAATCCCTACCCACCATCTTCATCTTTTCTAGATGAAGCGCAGCAGAACCCAGTATGCTGAAGAAACAGAAGAAAATGATGACTTCAACCCCGTTTACCCTTTTGACCCATTTGACACATCAGACGTACCCTTTGTTACACCCCCTTTTACTTCTTCCAATGGTCTTCAAGAAAAACCACCAGGTGTATTAGCACTTAATTACAAAGACCCCATTGTAACTGAAAATGGAACCCTTACACTCAAGCTAGGGGACGGAATAAAACTTAATGCCCAAGGTCAACTTACAGCTAGTAATAATATCAATGTTTTGGAGCCCCTTACCAACACCTCACAAGGTCTTAAACTTTCTTGGAGCGCCCCCCTAGCAGTAAAGGCTAGTGCCCTCACACTTAACACAAGAGCGCCCTTAACCACAACGGATGAAAGCTTAGCCTTAATAACCGCCCCTCCCATTACAGTAGAGTCTTCGCGTTTGGGCTTGGCCACCATAGCCCCTCTAAGCTTAGATGGAGGTGGAAACCTAGGTTTAAATCTTTCTGCTCCCCTGGACGTTAGTAACAACAATTTGCATCTCACCACTGAAACTCCCTTAGTTGTAAATTCTAGCGGTGCCCTATCTGTTGCTACTGCAGACCCCATAAGTGTTCGCAACAACGCTCTTACCCTACCTACGGCAGATCCGTTAATGGTGAGCTCCGATGGGTTGGGAATAAGTGTCACTAGTCCCATTACAGTAATAAACGGTTCCTTAGCCTTGTCTACAACTGCTCCCCTCAACAGCACAGGATCCACTTTAAGTCTGTCTGTTGCCAATCCTCTGACTATTTCACAAGACACATTGACTGTTTCCACTGGTAACGGTCTTCAAGTGTCGGGGTCTCAATTAGTAACAAGAATAGGGGATGGTTTAACATTCGATAATGGGGTCATGAAAGTAAACGTTGCCGGGGGAATGAGAACTTCTGGCGGTAGAATAATTTTAGATGTTAATTATCCCTTTGATGCGAGCAATAACCTGTCCTTAAGACGGGGATTGGGACTAATTTATAACCAATCTACAAACTGGAACTTAACAACTGATATTAGTACCGAAAAAGGTTTAATGTTTAGTGGCAATCAAATAGCTCTTAATGCAGGTCAGGGGCTTACATTTAATAATGGCCAACTTAGGGTTAAGTTGGGAGCTGGACTTATTTTTGATTCAAACAATAACATTGCCTTAGGCAGCAGCAGCAACACTCCATACGACCCTCTGACACTGTGGACAACTCCTGACCCACCACCAAACTGCAGCCTCATACAAGAGCTAGATGCAAAACTCACCCTGTGCTTAACAAAAAACGGATCTATTGTTAATGGCATTGTAAGTTTAGTGGGTGTTAAGGGTAATCTCCTAAATATCCAAAGTACTACTACCACTGTAGGAGTGCATTTAGTGTTTGATGAACAGGGAAGATTAATCACATCAACCCCTACTGCCCTGGTTCCCCAAGCTTCGTGGGGATATAGACAAGGCCAATCAGTGTCTACCAATACTGTTACCAATGGTCTAGGTTTTATGCCTAATGTGAGTGCTTACCCTAGACCAAATGCCAGTGAGGCTAAAAGCCAAATGGTAAGTCTCACGTACTTACAGGGAGATACATCTAAACCTATAACAATGAAAGTTGCATTTAATGGCATTACGTCGCTAAATGGATACTCTTTAACATTCATGTGGTCAGGTCTATCAAACTATATAAATCAGCCTTTCTCTACACCATCCTGCTCCTTTTCTTACATTACCCAAGAATAAAAACACACACAAAACACAAATTGCGTACTTATTGTTTATTTTTTTTTTTTTTTACACTATACGCGTGGTTAAACTGCCTCCTTCCCATTTTACCTTGTATACCTCCCTTTCCCCCTTTGTAGCTGAAAACAACTGCACTTGAATATTTCGACTTAGGTTTTTTGGCGTTAGCGTCCACACAGTTTCTTTACGGGCAAAGCGAGGGTCGGTGATGGAAACGAATCCCTCGCCCGCACAGTCACTCAAGCGGCATTCCCCATCCAAAACCAGGTCCATGATTTTATCCTACAAAAAGTAACAACAGTCAGTGTCCATCAGCCGCCCAAGGATTCTCTCGTTGATTATAATCTCCAAATAAAATTGCTCGATGATGCATAATTAAACCCTTTAGCAGTTGCTGACGATAACGTTCATGCCGACTATGTTTTAGAGGGCGAACAGTGTTTTCAGCAATTACTTGAACAACTTTTAACATTAGCAGTCTGGTACGACGAGCGCAACAGCGCATGCGTATCTCACTTAAGTCTTTACAATAATCACAACACAGCACTAACATGTTATTTAAAATTCCATAATTAAAGGCGCTCCATCCAAAACTAACTTTTTCTAACGCTAACCAGGCATGGCCATCATACATAATTTTAAAGTAAATTAAATGGCGACCTCTAACAAAGGTGCTTCCCACATACATCACCTCTTTAGGCATTAAATGGTTAACAACCTCCCGATACCAAAAACACCTTTTGTTAATTAAGGCGCCATATACGGCCATTTTGAACCAGCGTCCCAAAAGCATCCCAGCTGACATACACTGTAGTGAACCCGGACGCTGGCAATGACAATGAATAAGCCACCGCTCATGACCATGTAATAATTGAGTAACTTCAACATTTATAGTGGCACAACACATACATACACTCATGTATTTTTTCAAAATAAACATCTCATAATCAGTTAGAATCATATCCCACGGTATTGGCCATTCCTGCAGCACTGTAAAACCTACACATGAAGGAATGCCTCTTACCTCACTTACATTATGTAAAGTCAGACTATTACACTCAGGCCATAAAGAATTTTCCGAAGTACTCAACGTAGCTTTTGACTGTTCCTCACAGGGCGGTAGTTGGTACTTGTTGTATGGTGCCAATCTGTAGCGATACCGTCTGTCGCGCTGCATCGTAAACAACAGACTTGCGAGCGTCTTCGTACTTAAAAAAACAAAACCACGTACGACCACTGGTTATCGCACCTCGTCCTTTTTGTTTGCAGCGTTGGCGTTCCGTCAAAAAAGCAAAGTACAACCACTCTCGCAGGCTTGCTAAAATGTATTCAGCTTCAGGTGTTATCTTCAAATCATGATGTTTAATAAAGCGCAGAGTATCCACACAGGATGCATGGGCTAAACCAAGCCATGCTATGCAGGCAGCCGTGTCCCGACTTACAGGAGGAGGAGGAATACAAGGTAGAGGCATAAAAACTTAATCAAGACGGTCAGCAAGGATTTGAATGCGTAAATCTCGCAGGTGGCAGCGATCGCCTCCGCTGTGCTGGTGAAAGATCACAGCCAGATCAAATTGTAAGCGATTTTCCAAATGTTCAACAACAGCTTCTAAAAGAGCCACAGCTCTGATTTCGATAAACAAAAGCAAAGCAAATGCATTATCATGAAACTCTTCTATCATCAAACTGCCTGACTGAACCATTCCCAGGTAATTTTCATTCTTCCACTGTTGTATTATTTGAACACACTGATTTTGCAGGTTTAAACCGTGAATATTAAAAAGCTCTGTAAGGGCGCCCTCCACCGCCATCCGCAGGCAGTACTTCATATTTGCTGAAAAAAGTCTGGATCTTCAAACACCTGCAGTAAATTCAGTAGATTTACATTAGGCTCCACACCTTGGTCTCGCAGCTGACATCTTAATGCCAGTTGTATAAAATCATACAAATCAGAAGCCAGCAGCAAAGAAAGTTCACCTCCAGGTACAAGTTCCGGAGTTCCCACAGAACATACAACTTGCACAAATGGACCCATATTAGTAAGCGTGGCGCCAACGTAGACATCGCGCATAGGAGGAGTTAAATAATGCATTACCAGCAGCCAAAACTCAGGTAGCACGTCTTTAAGAAACGTCACCACCTCAAAATCTAAGCCATGCAAATAGTTCCGTAAAGACTCCGGAAACAACACGGAGTAATGAACAAGCGACCTCTGAAACATGCTTTAGGTTAGCCTGAAAAATAAAAATATGTTAAATTAAAGATGCCTGGCAAACGGGTGGAAAAACAACTCTACTTAAAAGCAAGCGCGCGACTGGCTGCTTTGCGCGAACATCGCAAAACACGTCGGAATGATTAAACAACAAAACACTGAGCTCCATTCTTGAGCCTGGATAAAGCGTTTCAGCGCCAACAAAAACCCCTCTGGCGTTCATGTCGCATAATGAAAACAATGTTCCCAAATATCCAGGAGGAATATCAACTGCTATGTGCAAATATAAAAGCACAACTCCATGTGGAGGTATAACAAAATTCGCAGGAGAAAATAACACATAAGCATTAGAGTCGCCCTCTTGTTTAGGCAACATAGCCCCAGGTCCCGTAAAATACACATAAAGAGTCTCAAAAGCAGCCATAATGCCTTACCAGAAAAACAGTACAAAGCCAGGCACAGCAGACACAATCTGCCGCAAGTGCGCACCTTTAATACTGAAAAATAGTGACGTAAATGGCCAAAGTTCGCCTACACAACACAAAAAAAACCCCAAAAGCCCGCGAAAAAAATCACTTCCGCATATGACTCGGCATAATACGGTGTTCTCACGACACGTCACATCCGGCGCGCCCGGCTCCCACGCCGCGCCCCACTTCCTCATCCGCCCAAACTTACAAGCACGCCAAAGCCACACCTCCACCCAATCAAATTACACACTACGCCCACTTCATTTTAATATTGGCACTAGTCCAGTATAAGGTATATTATTGGATAGG

>Bloc 1

ATCGATACCTATCTAATAATATACCTTATACTGGACTAGTGCCAATATTAAAATGAAGTGGGCGTAGTGTGTAATTTGATTGGGTGGAGGTGTGGCTTTGGCGTGCTTGTAAGTTTGGGCGGATGAGGAAGTGGGGCGCGGCGTGGGAGCCGGGCGCGCCGGATGTGACGTTTTAGACGCCATTTTACACGGAAATGATGTTTTTTGGGCGTTGTTTGTGCAAATTTTGTGTTTTAGGCGCGAAAACTGAAATGCGGAAGTGAAAATTGATGACGGCAATTTTATTATAGGCGCGGAATATTTACCGAGGGCAGAGTGAACTCTGAGCCTCTACGTGTGGGTTTCGATACGTGAGCGACGGGGAAACTCCACGTTGGCGCTCAAAGGGCGCGTTTATTGTTCTGTCAGCTGATCGTTTGGGTATTTAATGCCGCCGTGTTCGTCAAGAGGCCACTCTTGAGTGCCAGCGAGAAGAGTTTTCTCTGCCAGCTCATTTTCACGGCGCCATTATGAGAACTGAAATGACTCCCTTGGTCCTGTCGTATCAGGAAGCTGACGACATATTGGAGCATTTGGTGGACAACTTTTTTAACGAGGTACCCAGTGATGATGATCTTTATGTTCCGTCTCTTTACGAACTGTATGATCTTGATGTGGAGTCTGCCGGTGAAGATAATAATGAACAGGCGGTGAATGAGTTTTTTCCCGAATCGCTTATTTTAGCTGCCAGTGAGGGGTTGTTTTTACCGGAGCCTCCTGTACTTTCTCCTGTCTGTGAGCCTATTGGGGGCGAATGTATGCCACAACTGCACCCTGAAGATATGGATTTATTGTGCTACGAGATGGGCTTTCCCTGTAGCGATTCGGAAGACGAGCAAGACGAGAACGGAATGGCGCATGTTTCTGCATCCGCAGCTGCTGCTGCCGCTGATAGGGAACGTGAGGAGTTTCAGTTAGACCATCCAGAGTTGCCCGGACACAATTGTAAGTCCTGTGAGCACCACCGGAATAGTACTGGAAATACTGACTTAATGTGCTCTTTGTGCTATCTGCGAGCCTACAACATGTTCATTTACAGTAAGTGTGCTATGGGAGGTGGGAGGTGATTTTTTTTTCTTAAGCAGTGAAAAATAATATTTTGTTGTTTTTAGGTCCTGTTTCCGATAATGAGCCTGAACCTAATAGCACTTTGGATGGCGATGAGCGACCCTCACCCCCGAAACTAGGAAGTGCGGTTCCAGAAGGAGTAATAAAACCTGTGCCTCAGCGGGTGACTGGGAGGCGTAGATGTGCTGTGGAAAGCATTTTGGATTTGATTCAAGAGGAAGAAAGAGAACAAACAGTGCCTGTTGATCTGTCAGTGAAACGCCCTAGATGTAATTAATGGACTTTGAGCACCTGGGCAATAAAATAGGGGTAATGTGGTTTTTGTGAGTCATGTATAATAAAACTGGTTTCGGTTGAAGTGTCTTGTTAATGTTTGTTTGGGCGTGGTTAAACAGGGATATAAAGCTGGGTTGGTGTTGCTTTGAATAGTTCATCTTAGTAATGGAGTTGGAAACTGTGCTGCAAAGTTTTCAGAGCGTTCGCCAGCTCTTGCAGTATACCTCTAAAAACACTTCAGGTTTTTGGAGGTATCTGTTTGGCTCTACCTTAAGCAAGGTGGTAAATAGGGTGAAAGAAGACTATAGAGAGGAATTTGAAAACATATTGGCCGACTGTCCAGGGCTTTTGGCTTCACTAGACCTTTGTTACCACTTGGTGTTTCAGGAAAAAGTGGTCAGATCCTTAGATTTTTCATCTGTGGGACGAACGGTTGCTTCTATTGCTTTTTTGGCAACCATATTGGATAAATGGAGCGAGAAATCCCACCTGAGTTGGGATTACATGCTGGATTACATGTCAATGCAGCTGTGGAGGGCATGGCTGAAGAGGAGGGTTTGCATTTACTCGCTGGCGCGGCCTTTGACCATGCCGCCGCTGCCGACGTTGCAAGAGGAGAAGGAGGAGGAGCGGAACCCTGCGGTGGTGGAGAAGTAAACATGGAACAACAGGTGCAAGAAGGCCATGTACTTGACTCTGGCGAAGGGCCTAGTTGCGCAGATGATAGAGATAAGCAGGAAAAAAAAGAAAGTTTAAAGGAAGCTGCTGTTCTTAGTAGGCTAACTGTTAATCTGATGTCCCGCCCGCGTTTGGAAACTGTATATTGGCAGGAGTTGCAGGATGAATTTCAGCGGGGTGATATGCATTTACAGTACAAATACAGTTTTGAACAATTAAAAACCCACTGGTTAGAGCCATGGGAGGATATGGAGTGTGCTATTAAAGCTTTTGCTAAATTGGCCTTACGTCCTGATTGTAGCTACAGAATTACTAAAACAGTAACCATTACTTCATGCGCCTATATTATAGGTAACGGGGCAATAGTTGAGGTAGATACAAGCGACAGAGTTGCTTTTAGATGTCGAATGCAGGGTATGGGCCCAGGGGTGGTGGGTTTGGATGGAATTACATTTATAAATGTTAGGTTTGCTGGAGATAAGTTTAAAGGCATTATGTTCGAAGCTAATACCTGTCTTGTCTTGCATGGTGTTTACTTTCTTAACTTTAGTAACATTTGTGTAGAGTCTTGGAATAAGGTTTCTGCTAGGGGCTGTACTTTTTATGGATGTTGGAAGGGTTTGGTGGGTAGACCAAAAAGTAAACTGTCTGTAAAAAAGTGTTTGTTTGAAAAATGTGTACTTGCTTTAATTGTAGAGGGGGATGCACATATTAGGCATAATGCAGCTTCAGAAAATGCCTGTTTTGTATTATTGAAGGGAATGGCTATTTTAAAGCATAATATGGTTTGTGGGGTGTCTGATCAAACTATGCGACGTTTTGTTACCTGTGCTGATGGAAATTGTCATACCTTAAAAACTGTTCATATTGTGAGCCACAGTAGACATTGTTGGCCTGTATGTGATCATAACATGTTTATGCGCTGTACCATACATTTAGGCTTAAGGCGGGGTATGTTTAGACCTTCCCAATGTAACTTCAGCCACTCAAACATTATGCTGGAACCTGAAGTGTTTTCTAGAGTGTGTTTAAATGGGGTATTTGATTTATCTGTGGAATTATGTAAGGTTATAAGATATAATGATGATACTCGACATCGTTGCCGACAGTGTGAGTGTGGTAGCAGTCATCTAGAACTTCGTCCCATTGTGCTAAATGTAACTGAGGAGCTGAGAAGTGACCACCTTACCCTGTCTTGCCTGCGGACTGACTATGAGTCAAGTGATGAAGACGACAACTGAGGTAAGTGGGTGGAGCTAGGTATCGATATCTTTCTAGAAGATCTCCTACAATATTCTCAGCTGCCATGGAAAATCGATGTTCTTCTTTTATTCTCTCAAGATTTTCAGGCTGTATATTAAAACTTATATTAAGAACTATGCTAACCACCTCATCAGGAACCGTTGTAGGTGGCGTGGGTTTTCTTGGCAATCGACTCTCATGAAAACTACGAGCTAAATATTCAATATGTTCCTCTTGACCAACTTTATTCTGCATTTTTTTTGAACGAGGTTTAGAGCAAGCTTCAGGAAACTGAGACAGGAATTTTATTAAAAATTTAAATTTTGAAGAAAGTTCAGGGTTAATAGCATCCATTTTTTGCTTTGCAAGTTCCTCAGCATTCTTAACAAAAGACGTCTCTTTTGACATGTTTAAAGTTTAAACCTCCTGTGTGAAATTATTATCCGCTCATAATTCCACACATTATACGAGCCGGAAGCATAAAGTGTAAAGCCTGGGGTGCCTAATGAGTGAGCTAACTCACATTAATTGCGTTGCGCTCACTGCCAATTGCTTTCCAGTCGGGAAACCTGTCGTGCCAGCTGCATTAATGAATCGGCCAACGCGCGGGGAGAGGCGGTTTGCGTATTGGGCGCTCTTCCGCTTCCTCGCTCACTGACTCGCTGCGCTCGGTCGTTCGGCTGCGGCGAGCGGTATCAGCTCACTCAAAGGCGGTAATACGGTTATCCACAGAATCAGGGGATAACGCAGGAAAGAACATGTGAGCAAAAGGCCAGCAAAAGGCCAGGAACCGTAAAAAGGCCGCGTTGCTGGCGTTTTTCCATAGGCTCCGCCCCCCTGACGAGCATCACAAAAATCGACGCTCAAGTCAGAGGTGGCGAAACCCGACAGGACTATAAAGATACCAGGCGTTTCCCCCTGGAAGCTCCCTCGTGCGCTCTCCTGTTCCGACCCTGCCGCTTACCGGATACCTGTCCGCCTTTCTCCCTTCGGGAAGCGTGGCGCTTTCTCATAGCTCACGCTGTAGGTATCTCAGTTCGGTGTAGGTCGTTCGCTCCAAGCTGGGCTGTGTGCACGAACCCCCCGTTCAGCCCGACCGCTGCGCCTTATCCGGTAACTATCGTCTTGAGTCCAACCCGGTAAGACACGACTTATCGCCACTGGCAGCAGCCACTGGTAACAGGATTAGCAGAGCGAGGTATGTAGGCGGTGCTACAGAGTTCTTGAAGTGGTGGCCTAACTACGGCTACACTAGAAGAACAGTATTTGGTATCTGCGCTCTGCTGAAGCCAGTTACCTTCGGAAAAAGAGTTGGTAGCTCTTGATCCGGCAAACAAACCACCGCTGGTAGCGGTGGTTTTTTTGTTTGCAAGCAGCAGATTACGCGCAGAAAAAAAGGATCTCAAGAAGATCCTTTGATCTTTTCTACGGGGTCTGACGCTCAGTGGAACGAAAACTCACGTTAAGGGATTTTGGTCATGAGATTATCAAAAAGGATCTTCACCTAGATCCTTTTAAATTAAAAATGAAGTTTTAAATCAATCTAAAGTATATATGAGTAAACTTGGTCTGACAGTTACCAATGCTTAATCAGTGAGGCACCTATCTCAGCGATCTGTCTATTTCGTTCATCCATAGTTGCCTGACTCCCCGTCGTGTAGATAACTACGATACGGGAGGGCTTACCATCTGGCCCCAGTGCTGCAATGATACCGCGAGACCCACGCTCACCGGCTCCAGATTTATCAGCAATAAACCAGCCAGCCGGAAGGGCCGAGCGCAGAAGTGGTCCTGCAACTTTATCCGCCTCCATCCAGTCTATTAATTGTTGCCGGGAAGCTAGAGTAAGTAGTTCGCCAGTTAATAGTTTGCGCAACGTTGTTGCCATTGCTACAGGCATCGTGGTGTCACGCTCGTCGTTTGGTATGGCTTCATTCAGCTCCGGTTCCCAACGATCAAGGCGAGTTACATGATCCCCCATGTTGTGCAAAAAAGCGGTTAGCTCCTTCGGTCCTCCGATCGTTGTCAGAAGTAAGTTGGCCGCAGTGTTATCACTCATGGTTATGGCAGCACTGCATAATTCTCTTACTGTCATGCCATCCGTAAGATGCTTTTCTGTGACTGGTGAGTACTCAACCAAGTCATTCTGAGAATAGTGTATGCGGCGACCGAGTTGCTCTTGCCCGGCGTCAATACGGGATAATACCGCGCCACATAGCAGAACTTTAAAAGTGCTCATCATTGGAAAACGTTCTTCGGGGCGAAAACTCTCAAGGATCTTACCGCTGTTGAGATCCAGTTCGATGTAACCCACTCGTGCACCCAACTGATCTTCAGCATCTTTTACTTTCACCAGCGTTTCTGGGTGAGCAAAAACAGGAAGGCAAAATGCCGCAAAAAAGGGAATAAGGGCGACACGGAAATGTTGAATACTCATACTCTTCCTTTTTCAATATTATTGAAGCATTTATCAGGGTTATTGTCTCATGAGCGGATACATATTTGAATGTATTTAGAAAAATAAACAAATAGGGGTTCCGCGCACATTTCCCCGAAAAGTGCCACCTGACGTCTAAGAAACCATTATTATCATGACATTAACCTATAAAAATAGGCGTATCACGAGGCCGCCCCTGCAGCCGAATTATATTATTTTTGCCAAATAATTTTTAACAAAAGCTCTGAAGTCTTCTTCATTTAAATTCTTAGATGATACTTCATCTGGAAAATTGTCCCAATTAGTAGCATCACGCTGTGAGTAAGTTCTAAACCATTTTTTTATTGTTGTATTATCTCTAATCTTACTACTCGATGAGTTTTCGGTATTATCTCTATTTTTAACTTGGAGCAGGTTCCATTCATTGTTTTTTTCATCATAGTGAATAAAATCAACTGCTTTAACACTTGTGCCTGAACACCATATCCATCCGGCGTAATACGACTCACTATAGGGAGAGCGGCCGCCAGATCTTCCGGATGGCTCGAGTTTTTCAGCAAGAT

>Bloc 2

GATATCGATAAGTGGGTGGAGCTAGGTGGGATTATAAAAGGCTGGAAGTCAACTAAAAATTGTTTTTGTTCTTTTAACAGCACGATGAACGGAACTACTCAGAACAACGCTGCGCTTTTTGATGGAGGGGTTTTTAGCCCTTATTTGACTTCCAGGTTACCATATTGGGCCGGAGTACGTCAGAATGTGGTAGGATCTACAGTGGACGGTCGACCTGTGGCACCTGCAAATTCATCAACATTAACCTATGCAACTATTGGACCCTCGCCTTTGGATACCGCCGCCGCCGCTGCAGCTTCCGCGGCCGCTTCTACGGCTCGCAGTATGGCAGCTGATTTCAGCTTCTACAATCACTTGGCTTCGAATGCTGTGACACGCACCGCAGTTCGAGAGGACATTCTGACTGTTATGCTTGCCAAGCTTGAAACTCTAACTGCTCAGCTGGAAGAGCTATCGCAAAAGGTTGAGGAATTAGCTGATGCTACTACCCATACCCCAGCCCAACCTGTAACCCAATAAAGAAAAAACTTAAATTGAGATGGTGTTATGAATCTTTATTGATACTTGTTTTTTCTGACATGGTAAGCTCTTGACCACCGTTCCCTATCATTAAGAACACGGTGAATGTGTTCCAGTATTTTGTAAAGATGAGCCTGTATATTAAGGTACATTGGCATTAGGCCATCTTTGGGATGAAGGTAGGACCATTGAAGGGCTTCATGTTCCGGGTTAGTGTTGTAGATAATCCAGTCATAGCAACAACGCTGGGCATGGTGATTAAATATATCTTTTAACAACAAGCTAATTGCTAATGGAAGACCTTTAGTATAGGTATTGATAAAACGGTTAAGCTGGGTGGGATGCATCCGAGGTGACATGATATGAAGTTTTGATTGTATTTTGAGATTGGCAATGTTACCTGCCAAATCTCTTCTTGGATTCATATTGTGGAGAACCACGAAAACGGTGTAGCCAGTACACTTGGGAAATTTGTCATGGAGTTTAGAAGGAAAGGCATGGAAAAACTTGGAAACGCCTTTGTGACTTCCCAAATTTTCCATACACTCATCCATTATTATGGCAATTGGACCGCGAGCAGCGGCTTGAGCAAAAATGTTTTCTGGATCAGAAACATCATAGTTGTGGTCTAGAGTTAGGTCATCGTAGGACAACTTAACAAATTTAGGACACAGCGTTCCAGATTGTGGAATAATAGTTCCCTCTGGTCCTGGGACATAATTTCCCTCACAAATTTGCATTTCCCAAGATTTAATTTCAGATGGGGGAATCATGTCCACTTGCGGAACAATAAAAAAAACAGTTTCTGGAGCAGGTGTAACCAGCTGGGCAGAAAGCAAATTACGCAACAACTGAGACTTCCCACAGCCAGTGGGTCCATAAATTACCCCAATTACAGGTTGCAAGTGATAGTTTAACGAGGTGCAGCTGCCGTCTTCGTGGAGAAGCGGAGCCACTTCATTCATCATTTGTCGGACGCGGATGTTTTGCTTGGCCAGTTCCCCTAACAGACGCTCTCCGCCTAAGGAAAGTAACTCTTGTAAAGATTTGAAATTTTTAAGTGGCTTTAGGCCATCGGCCATAGGCATGTGGTCCAGGGTTTGCTTCAGCAGTTGCAAGCGATCCCATAGCTCAGTTATATTTTCTATGCCATCTCGATCCAGCAAACTTCCTCGTTGCGGGGGTTTGGCTGGCTGTTGCTGTAAGGAACGAGGCGGTGAGCATCCAAATGGACGAGGGTTTTGTCCTTCCAGGGACGTAATGTGCGCGTCAGGGTTGTTTCGGTCACGGTGAATGGATGCGCTCCTGGTTGAGCGCTGGCCAGTGTGCGCTTTAAACTGAGGCGGCTGGTGCTGAAGCGCGTGTCTTCTCCCTGTGCTTCGGCAAGGTAGCATTTTAACATAAGATCATAAGACAAAGCCTCTGTAGCGTGGCCTTTAGCCCGTATTTTTCCTTTGGAGGTGCTCCCGCAGTGAGGACACTGAAGGCATTTAAGGGCGTACAGTTTTGGAGCCAAAAAAACAGATTCTGGAGAATAAGCATCTGCGCCACAATAACTACAAACAGTTTCACATTCAACTGACCAGGTCAGCTCAGGACATGATGGATCAAAAACAAGTTTCCCTCCGTACTTTTTGATGCGTTTCTTACCTTGCGACTCCATAAGGCGGCGTCCTTTCTCTGTGACAAAAAGACTGTCAGTGTCTCCGTATACAGATTTAAGGGGTCTATCCTTCAGTGGTATTCCGCGGTCCTCCTCGTACAGGAATTCTGACCACTCTGACACAAAAGCTCTAGTCCAAGCAAGTACAAAGGAAGCCACATGGGAAGGGTACCGATCGTTGTTAATTAAAGGGTTAGAACTTTCTAAGGTGTGTAAACACATGTCTCCTTCTTCAGCGTCCATGAATGTGATTGGTTTGTAGGTGTAAGTCACGTGTTCACAATTTTCTGGTGGTGGGCTATAAAAAGGGGCGGGTCCTTGGTCTTCATCGCTTTCTTCTGCTTCGCTGTTTACGAGCGCCAACTGGTTGGGTGAGTACACGCGCTCAAAGGCAGGCATTACCTCTGTACTCAACGTGTCAGTTTCTATAAACGATGAGGATTTGATGTTTAATCGCCCCGCTGCAATTTCTTTCATTAGGCTTTCTTCCATTTGATCAGAAAAAACTATTTTTTTGTTATCTAGTTTGGTAGCAAAAGATCCGTACAAGGCATTGGAAAGCAGCTTGGCTATAGATCTTAGGGTTTGATTTTTGTCCCTATCGGCCCGTTCTTTTGCGGCAATATTGAGTTGCACATATTCGCGTGCCAGGCATTTCCAGGTGGGGAAAATGGTGGTGCGCTCGTCAGATAGCAAGCGTAAGCGCCACCCGCGATTATGCAGTGTAACCAGATCTACGCTGGTAACTACTTCACCGCGCAAGCTTTCATTGGTCCAGGCTAAACGACCGCCTTTTCTAGAACAAAAAGGAGGAAGAACATCCAACTGATTTTCATCTGGGGGGTCGGCATCTATAGTAAAAATGCCAGGACAAAGATTTTTGTCAAAATAATCAATTTTGCAAGTGTAATTTTCCAGCGCCACCTGCCATTGCCGCACGGCCAATGCCCGCTCATAGGGGTTAAGGGGAGGACCCCAAGGCATGGGGTGTGTGAGGGCCGATGCATACATGCCGCAAATATCATATACATATATGGGCTCTTTTAGTACTCCTATGTAAGTAGGATAGCACCTGCCGCCACGAATGCTGGCGCGAACGTAGTCATATAGCTCATGTGAAGGCGCCAGGATGTTGGGCCCAAGATGTGTGCGCTGTGGTTTTTCGGCGCGGTACAAAATTTGTCTGAAAATTGCATGAGAGTTAGAGGAAATGGTAGGACGCTGAAACACATTAAAATGTGCCGCGTCAAGACCCACTGCGTCAGTAACAAACTGGGCGTATGAGCTACGCAGTTTTTCTACCAATGAGGCAGTCACAAGTACATCCAGGGCACAATAGTTTAATGTTTCCCCGATAAGATTGTAATTTTTTTCTCCTTTTTTTTTCCATAGTTCTTGATTTAGGAGGTATTCCTCCTTATCCTTCCAGTACTCCTCCAGGGGAAACCCATTTGCATCTGCACGGTAAGAACCAAGCATATAAAACTGATTTACCGCCTTGTACGGACAACATCCTTTTTCTACAGGCAGGGCATACGCTTGTGCAGCCTTTCTTAAAGATGTATGAGTAAGAGCAAAGGTATCTCTGACCATTACTTTTAAATACTGGTATTTAAAATCTTGGTCGTCACACCCTCCGTGTTCCCACAGTAGGAAGTTAGTTCGCTTTTTGTAGTGGGGATTGGGAAGGGCAAAAGTAATATCATTAAATAATATTTTGCCAGCTCTTGGAATAAAATTTCTAGAAATTTTAAAGGGTCCAGGGACGTCCAAGCGGTTATTGATTACCTGAGCGGCAAGAACAATTTCATCAAATCCATTAATATTGTGTCCTACTATATACAACTCTACAAATCTTGGCTCACCCTTAATTGCAGGGGCTCTTTTAAGATCTTCGTAGGAAAGATCTTCAAGCGCGACTAGTCCGTTTTCTTCTTGAGCCCATTGAGACAAGTGTGGATTTTTTTGTAAAAAAGTCATCCAAAGATCAGTAGCTAAGGAGGTTTGTAAGCGGTTTCTATAGGTACGAAACTGTTGACCGACCTTCATTTTTTCTGGGGTTAAGCAGTAGAAAGTAGTAGAGTCTTTTTCCCATTGGTCCCATCCAAGTTCTAATGCAAGTTGTAAGGCATGTTTGACAAGATTGTCATCCCCAGACAGTTTCATCACCAGCATAAATGGGACAAGTTGCTTTCCAAATGCCCCCATCCAGGTGTAGGTTTCTACATCATAGGTAATAAAAAGGCGCTCAGTGCGAGGATGCGAACCGATTGGGAAAAAGTGGATCTCCTGCCACCAGTTGGAAGAATGGCTGTTGATGTGATGAAAGTAGAAATCTCGTCGGCGGACAGAGCATTCATGCTGATGTTTGTAAAAGCGTGCGCAGTGTTCGCATCGTTGCACGGGCTGTATCTGTTGAATGAGGTGTACCTGGCGGCCTCGCACCAGAAAGCAGATGGGAAAATCAATACCACTTGGCAGCTGCCGTTCGTCCTCTTCCTCTTCTGCTGCATTGCCACTACCGTTTGGATCCTCGAAAGCGAGAACGGAGAGGGTGACGGTGCCCCTCGAGCTGCATGTCCAGATTTCAGCACGAGAGGGGCGGAAACGGGAAATCAGGGCGTACAGCCTGGAGCTGTCCATGGTATCAGTCAGAGAGAAAAGCATGTCCGCGGGGACAGCGCGCAAGTTGACTTCGCACAGGCGGGTAAGAGCAGGCTGGAGGTGCAGGTAATACTTAATTTCTAGAGGCGTGCCGTTGGCAGAGTCTATTGCGTGAAGTATTCCATGAGCCCGGGGACTAACCACGGTTCCACGGTGCACTTTTCCAATGCGCCTGCTTAAAATCGGCGGCGCGGACGAGCTCCCGGAGGAAGCGGCGGTTCGGGTCCTGCGGGAAGCGGGGGAAGCGGTATGTCGGCCTGACGCTCTGGCAGGGGAAGGTGTTGAGCCCGAAGTTGACTGGCATGGGCGACTACCCGGCGATTGATATCTTGAATCTGTCGGCGTTGTGTAAACACTACCGGCCCTGTTGTTTTGAACCTGAAAGAAAGTTCAACAGAATCAATCTCAGTGTCATTTACTGCAGCCTGTCTTAAAATCTCCTGAACGTCGCCTGAGTTATCTTGGTAGGCAATTTCTGCCATTAATTGATCAATTTCTTCCTCCTGGAGGTCTCCATGTCCCGCACGTTCAATAGTGGCTGCAAGGTCATTAGATATCCGACTCATAAGCTGTGAAAATGCGTTTAGTCCAATTTCGTTCCAGACTCGGCTGTATACTACCCCTCCTTCGCTGTCCCGAGCGCGCATAACCACTTGCGCCAAGTTGAGTTCCACGAGCCGTGCGAACACGCCGTAGTTGCGCAAGCGCTGAAACAGGTAGTTTAAGGTGGTGGCAACGTGTTCTGAGACGAAGAAATACAGAATCCACCGACGAAGCGTCAGCTCGTTGATGTCACCTAAGGCTTCAAGACGTTCCATGGCTTCGTAAAAGTCTACTGCAAAATTGAAAAACTGGGAGTTGCGAGCTGCCACCGTCAATTCTTCTTCCAACAGACGAATAAGCTCGGCCACCGTCTCGCGCACTTCTTGCTGAAATGCGCCCGGAACTATTTCTTGTTCTTCCTCTTCTACCTCCATTATTTCTTCCTCGACCACAGGTGGTGGGGGTTGTCTTCTTCGACGCCGGCGAACGGGCAGCCTGTCTACAAATCTTTCAATCATTTCGCCGCGACGGCGGCGCATAGTTTCGGTTACTGCTCGACCGTTTTCACGTGGTCGTAACTCAAAAACTCCACCTCTAAGTTCTGTTTCATGTAAAATGGGAAATGAGGCGTTGCGAGGGGCGTTAGGTAGGGATACAGCGCTGATTATGCATTTTATTATTTGCTGCGTAGGAACTCCGCGCAAGGAGCTAAGCGTCTGCATATCCACCGGGTCGGAGAACCTTTCAAGAAAGGCATCTAGCCAGTCACAGTCACAAGGTAGGCTAAGTTTTGTTTCTTCTAAAGTACCAGGAAGCTGAGCAATATCGATATCTTGCTGAAAAACTCGAGCCATCCGGAAGATCTGGCGGCCGCTCTCCCTATAGTGAGTCGTATTACGCCGGATGGATATGGTGTTCAGGCACAAGTGTTAAAGCAGTTGATTTTATTCACTATGATGAAAAAAACAATGAATGGAACCTGCTCCAAGTTAAAAATAGAGATAATACCGAAAACTCATCGAGTAGTAAGATTAGAGATAATACAACAATAAAAAAATGGTTTAGAACTTACTCACAGCGTGATGCTACTAATTGGGACAATTTTCCAGATGAAGTATCATCTAAGAATTTAAATGAAGAAGACTTCAGAGCTTTTGTTAAAAATTATTTGGCAAAAATAATATAATTCGGCTGCAGGGGCGGCCTCGTGATACGCCTATTTTTATAGGTTAATGTCATGATAATAATGGTTTCTTAGACGTCAGGTGGCACTTTTCGGGGAAATGTGCGCGGAACCCCTATTTGTTTATTTTTCTAAATACATTCAAATATGTATCCGCTCATGAGACAATAACCCTGATAAATGCTTCAATAATATTGAAAAAGGAAGAGTATGAGTATTCAACATTTCCGTGTCGCCCTTATTCCCTTTTTTGCGGCATTTTGCCTTCCTGTTTTTGCTCACCCAGAAACGCTGGTGAAAGTAAAAGATGCTGAAGATCAGTTGGGTGCACGAGTGGGTTACATCGAACTGGATCTCAACAGCGGTAAGATCCTTGAGAGTTTTCGCCCCGAAGAACGTTTTCCAATGATGAGCACTTTTAAAGTTCTGCTATGTGGCGCGGTATTATCCCGTATTGACGCCGGGCAAGAGCAACTCGGTCGCCGCATACACTATTCTCAGAATGACTTGGTTGAGTACTCACCAGTCACAGAAAAGCATCTTACGGATGGCATGACAGTAAGAGAATTATGCAGTGCTGCCATAACCATGAGTGATAACACTGCGGCCAACTTACTTCTGACAACGATCGGAGGACCGAAGGAGCTAACCGCTTTTTTGCACAACATGGGGGATCATGTAACTCGCCTTGATCGTTGGGAACCGGAGCTGAATGAAGCCATACCAAACGACGAGCGTGACACCACGATGCCTGTAGCAATGGCAACAACGTTGCGCAAACTATTAACTGGCGAACTACTTACTCTAGCTTCCCGGCAACAATTAATAGACTGGATGGAGGCGGATAAAGTTGCAGGACCACTTCTGCGCTCGGCCCTTCCGGCTGGCTGGTTTATTGCTGATAAATCTGGAGCCGGTGAGCGTGGGTCTCGCGGTATCATTGCAGCACTGGGGCCAGATGGTAAGCCCTCCCGTATCGTAGTTATCTACACGACGGGGAGTCAGGCAACTATGGATGAACGAAATAGACAGATCGCTGAGATAGGTGCCTCACTGATTAAGCATTGGTAACTGTCAGACCAAGTTTACTCATATATACTTTAGATTGATTTAAAACTTCATTTTTAATTTAAAAGGATCTAGGTGAAGATCCTTTTTGATAATCTCATGACCAAAATCCCTTAACGTGAGTTTTCGTTCCACTGAGCGTCAGACCCCGTAGAAAAGATCAAAGGATCTTCTTGAGATCCTTTTTTTCTGCGCGTAATCTGCTGCTTGCAAACAAAAAAACCACCGCTACCAGCGGTGGTTTGTTTGCCGGATCAAGAGCTACCAACTCTTTTTCCGAAGGTAACTGGCTTCAGCAGAGCGCAGATACCAAATACTGTTCTTCTAGTGTAGCCGTAGTTAGGCCACCACTTCAAGAACTCTGTAGCACCGCCTACATACCTCGCTCTGCTAATCCTGTTACCAGTGGCTGCTGCCAGTGGCGATAAGTCGTGTCTTACCGGGTTGGACTCAAGACGATAGTTACCGGATAAGGCGCAGCGGTCGGGCTGAACGGGGGGTTCGTGCACACAGCCCAGCTTGGAGCGAACGACCTACACCGAACTGAGATACCTACAGCGTGAGCTATGAGAAAGCGCCACGCTTCCCGAAGGGAGAAAGGCGGACAGGTATCCGGTAAGCGGCAGGGTCGGAACAGGAGAGCGCACGAGGGAGCTTCCAGGGGGAAACGCCTGGTATCTTTATAGTCCTGTCGGGTTTCGCCACCTCTGACTTGAGCGTCGATTTTTGTGATGCTCGTCAGGGGGGCGGAGCCTATGGAAAAACGCCAGCAACGCGGCCTTTTTACGGTTCCTGGCCTTTTGCTGGCCTTTTGCTCACATGTTCTTTCCTGCGTTATCCCCTGATTCTGTGGATAACCGTATTACCGCCTTTGAGTGAGCTGATACCGCTCGCCGCAGCCGAACGACCGAGCGCAGCGAGTCAGTGAGCGAGGAAGCGGAAGAGCGCCCAATACGCAAACCGCCTCTCCCCGCGCGTTGGCCGATTCATTAATGCAGCTGGCACGACAGGTTTCCCGACTGGAAAGCAATTGGCAGTGAGCGCAACGCAATTAATGTGAGTTAGCTCACTCATTAGGCACCCCAGGCTTTACACTTTATGCTTCCGGCTCGTATAATGTGTGGAATTATGAGCGGATAATAATTTCACACAGGAGGTTTAAACTTTAAACATGTCAAAAGAGACGTCTTTTGTTAAGAATGCTGAGGAACTTGCAAAGCAAAAAATGGATGCTATTAACCCTGAACTTTCTTCAAAATTTAAATTTTTAATAAAATTCCTGTCTCAGTTTCCTGAAGCTTGCTCTAAACCTCGTTCAAAAAAAATGCAGAATAAAGTTGGTCAAGAGGAACATATTGAATATTTAGCTCGTAGTTTTCATGAGAGTCGATTGCCAAGAAAACCCACGCCACCTACAACGGTTCCTGATGAGGTGGTTAGCATAGTTCTTAATATAAGTTTTAATATACAGCCTGAAAATCTTGAGAGAATAAAAGAAGAACATCGATTTTCCATGGCAGCTGAGAATATTGTAGGAGATCTTCTAGAAA

>Bloc 3

TAATACGACTCACTATAGGGAGAGCGGCCGCCAGATCTTCCGGATGGCTCGAGTTTTTCAGCAAGATATCGATAAGTACCAGGAAGCTGAGCAATGCTACTAATAATGTAATTGAAGTAAGCTGTTTTAAGCCCACGAATGGTTTTAAGAAGCACCACATCTTTGGGTCCGGCTTGTTGAATTCGCAGGCGGTCTGCCATTCCCCACACGTCACTTTGACATCGTCCAAGATCTTTGTAGTAGTCTTGCATTAACCTTTCCACCTCTACCTCGCGGTTTCCGCGATCAGCCATGTGCGTGCTTCCGTAGCCTTGCAGCGGTTGTAATAAAGCTAAATCTGCCACTACCCGTTCCGCAAGCACTGCCTGTTGAATTTGGGTAAGGGTGGTTGCAAAGTCATCCACATCTACAAAGCGGTGATAAGCTCCTGCATTAATGGTGTAGCTGCAGTTTGTCATTACTGACCAATTAACAGTTTGCGTGCCTGGCTGTACAGTTTCTGTGTATCGCAAGCGTGAGTAAGCCCGAGAGTCAAAAACATAGTCATTGCAGGTGCGCACTAGGTATTGATAGCCCACAAGGAAATGAGGAGGAGGTTCGCGATACAACGGCCAGCCAAGCGTAGCCGCAGCACCTGGAGCGAGATCTTCCAACATGAGGCGGTGGTATTCATATATGTATCTGGACATCCATGTGATGCCGGCAGCGGTAGTTGTTGCTCGCATAAATTCGCGGGCTCGGTTCCAAATATTGCGCAGGGGTAAAAAGCGTTCAATAGTTGCCACGCTTTGACCGGTCAGGCGTGCGCAGTCTTGAATGCTCTGGACATGGAAAAAATGAAAGTCGGTAAGCGACTCCCTTCCGTGGTTTGGTGGAAAAGTCACAAGGGTACCATAGCGAGGAACCCCGGTTCGAAACCGGCAGGATCCGCTATGAGCACAAGTGAGGCGCTTGCGCGTTGAACCCGGCCAAGGACCCCCAGACACGGAGAGGAGTCTTTTTTTATTTATTTTTTCTTAGATGCATCCTGTCCTGCGACAAATGCGACCTCAGCCCAGGGCAACCACGGCCTCAGCAGCGGTGGCGCTTTCGGGCTCTGGCGAACAGGAAGAGCCTCAATGTCCTACATTGGAGTTGGAAGAAGGAGAAGGCATAGCCCGATTGGGCGCCCACTCTCCTGAGCGTCACCCAAGGGTGCAGCTCGCCCGGGACAGTCGCGTGGCATTTGTGCCTCGTCAGAACATGTTTCGCGACAACAGCGGGGAGGAAGCTGAGGAAATGCGAGACTGCAGGTTTAGGGCCGGTCGCGAGCTGCGCCGCGGATTTAATCGCGAGCGACTGCTGCGTGAGGAGGACTTTGAGCCAGATGAACATTCGGGGATTAGTTCTGCACGGGCCCATGTATCAGCAGCCAACTTAGTAACAGCATATGAACAAACGGTTACAGAGGAACGTAACTTTCAAAAAAGCTTTAATAACCATGTGCGCACACTAATAGCGCGAGAAGAAGTAGCCATTGGTTTAATGCATCTTTGGGACTTTGTAGAAGCTTATGTACATAATCCAGCAAGTAAACCCCTAACTGCCCAGCTGTTCTTAATAGTTCAACATAGTAGAGACAATGAAACTTTTAGGGATGCAATGCTTAACATAGCTGAACCCCAGGGTCGGTGGTTACTCGATTTAATTAACATTCTGCAGAGCATTGTGGTTCAGGAACGCAGTCTTAGTTTGGCAGACAAGGTGGCCGCCATTAATTACTCCATGTTAAGTTTGGGAAAGTTTTATGCTCGTAAAATCTACAAAAGTCCGTATGTTCCCATTGACAAGGAAGTGAAGATAGACAGCTTTTATATGCGCATGGCTTTAAAGGTACTAACATTAAGCGACGATCTTGGAGTGTACCGCAATGACCGAATCCACAAAGCAGTAAGCGCCAGTCGCCGCAGAGAGCTAAGCGACAAAGAGCTTATGCATAGCTTACAAAGGGCGCTGACGGGAGCAGGAACAGAGGACGAGTCGTTCTTTGATATGGGCGCAGACCTACGGTGGCAGCCAAGCGCTCGCGCTTTGGAGGCAGCTGGAGTGGCGTCTGCTGACGTCACTGGCGATGACGATGACGAAGACCAGTACGAGGACTGATCGGCCGTACCTTTTGTTAGATGCAGCGACCGGCGATCATCGCGGAGAGGGCTCCTAACCTGGATCCCGCGGTTTTGGCGGCCATGCAAAGCCAGCCTTCTGGCGTTACAGCTTCAGATGACTGGACAGCGGCCATGGATCGTATTATGGCTTTAACGGCGCGCAGTCCTGATGCTTTTCGCCAGCAGCCCCAAGCTAACCGCTTTTCGGCCATTTTGGAAGCAGTAGTGCCGTCTCGTACTAACCCTACTCACGAGAAAGTGTTAACCATTGTAAATGCTTTGTTGGATAGCAAAGCCATCCGCAAAGATGAGGCTGGTTTAATATACAACGCTTTGCTTGAGCGCGTGGCACGCTATAACAGTACCAATGTGCAGGCTAACTTAGACCGGATGGGTACAGATGTAAAGGAGGCGCTGGCTCAACGAGAGCGCTTTCATCGCGATGGTAATCTTGGTTCGCTAGTAGCATTAAACGCTTTTTTGAGTACTCAGCCGGCTAATGTTCCGCGTGGTCAGGAAGATTATACAAACTTCATCAGCGCCTTGCGACTAATGGTTACTGAAGTGCCTCAAAGTGAAGTGTATCAGTCTGGACCCGATTACTTTTTTCAAACGTCCAGGCAGGGTTTGCAAACCGTAAACTTAACTCAGGCTTTTAAAAATTTGCAAGGTTTGTGGGGGGTTCGTGCTCCAGTAGGCGATCGTTCAACTTTGTCCAGTTTACTAACACCAAACTCGCGCCTATTACTGTTGCTAATTGCCCCCTTTACCAACACCAACAGTTTAAGTCGAGATTCATACCTGGGTCACTTAGTTACTTTGTACCGCGAAGCCATTGGTCAAGCGCAGGTAGACGAACAAACTTATCAAGAAATAACCAGTGTTAGTCGCGCACTGGGCCAGGAGGACACTGGCAGTTTAGAGGCCACACTTAACTTTTTACTAACTAACCGTCGCCAGCAAGTGCCTCCTCAGTACACTTTAAATGCGGAAGAAGAACGCATATTGCGCTATGTACAGCAATCTGTAAGTTTGTATCTTATGCGTGAGGGTGCCACCCCCAGTGCCGCCTTAGACATGACAGCGCGCAATATGGAGCCGTCCTTCTACGCTTCCAATCGAGCTTTCATTAATCGCTTGATGGATTACCTTCACCGCGCTGCGGCCATGAACGGGGAATACTTTACAAATGCAATTCTAAATCCGCATTGGTTGCCCCCTCCTGGATTTTACACTGGTGAATTTGATTTGCCGGAAGGAAATGATGGCTTTTTGTGGGATGATGTTACGGACAGTCTGTTTAGTCCTGCAGTTATTGGACACCATGGTAAAAAGGAAGCAGGTGATGAAGGTCCCTTGCTTGACTCTCGGGCGAGTTCTCCATTCCCCAGTTTAACTAGTTTACCCGCCAGTGTTAACAGCGGTCGTACCACCAGACCCCGACTAACAGGTGAAAGTGAATACTTAAATGACCCCATCTTGTTTCCAGTGCGCGACAAAAATTTTCCCAACAATGGCATAGAAAGTTTGGTAGATAAAATGTCTCGCTGGAAAACATATGCACAAGAGCGGCGAGAATGGGAGGAAAGACAGCCAAGACCAGTTCGCCCTCCTAGGCAACGTTGGCAGCGACGCAAAAAAGGGGCACATGCGGGGGATGAAGGAAGCGATGACTCAGCTGACGACAGTAGTGTATTAGATTTAGGAGGGTCAGGAAACCCATTTGCTCATTTGCGCCCACAGGGTTGCATAGGGTCATTGTATTAAATTGAATAAAAGCATACTTACCAAAGCCATGGCGACCAGTGTTCGTCTTATTTTCCTTCTTCCGTTAGCTGTGAAATGAGGCGCGCGGTGGAACTGCAGACAGTGGCTTTTCCTGAGACACCACCTCCCTCTTACGAAACCGTGATGGCAGCGGCGCCACCCTACGTGCCTCCCCGCTATTTGGGTCCTACGGAGGGAAGAAACAGTATCCGTTACTCGGAATTGTCACCGTTGTACGATACCACTCGAGTGTACTTGGTGGACAACAAGTCTTCTGACATTGCTTCATTGAATTACCAGAATGATCACAGCAACTTTTTAACCACTGTAGTGCAAAATAATGACTATTCCCCTATAGAGGCTGGCACGCAAACTATTAACTTTGATGAAAGGTCTAGATGGGGTGGAGATTTAAAAACCATCTTACATACCAACATGCCAAACGTGAACGATTTTATGTTTACCACCAAATTTAAGGCCAGGGTAATGGTGGCTAGGAAAACAAACAACGAAGGCCAAACCATTTTAGAATATGAGTGGGCAGAATTTGTGCTACCCGAGGGTAACTATTCGGAAACCATGACTATTGACTTAATGAACAATGCTATTATTGAGCATTATTTGCGAGTAGGAAGACAGCATGGAGTGCTGGAAAGTGACATTGGAGTTAAGTTTGACACCAGAAACTTTCGTCTGGGTTGGGACCCCGAAACCCAATTAGTAACTCCGGGAGTGTACACTAATGAGGCTTTTCATCCAGATATAGTACTGCTTCCAGGTTGCGGGGTTGATTTTACAGAGAGCAGATTAAGCAACATACTAGGTATAAGAAAGAGGCAGCCGTTTCAGGAAGGATTTGTGATTATGTATGAACACTTAGAGGGAGGCAATATTCCAGCTCTTTTGGATGTAAAAAAATACGAAAACAGTCTGCAGGATCAAAACACTGTAAGAGGAGACAACTTTATTGCCTTAAATAAGGCTGCTAGGATTGAACCGGTTGAAACAGACCCCAAAGGACGCAGTTACAACTTGCTTCCAGACAAAAAAAATACTAAATATCGCAGCTGGTATTTGGCATACAACTACGGAGACCCAGAAAAAGGAGTTCGGTCATGGACTCTACTAACAACTCCAGATGTAACAGGCGGCTCCGAACAGGTGTACTGGTCCCTACCCGATATGATGCAAGATCCGGTGACTTTTCGCTCCTCGCGTCAAGTTAGCAACTATCCTGTAGTTGCAGCAGAATTACTGCCAGTTCATGCTAAAAGCTTCTACAACGAGCAAGCCGTCTACTCACAGCTTATTCGCCAGTCAACCGCGCTTACGCGCGTGTTTAATCGCTTTCCCGAGAACCAGATACTGGTGCGTCCACCAGCCGCTACCATCACTACCGTCAGTGAAAACGTTCCCGCCCTTACAGATCACGGGACCCTGCCGCTGCGTAGCAGTATCAGTGGAGTTCAGCGAGTCACCATCACTGACGCCCGCCGCCGGACCTGTCCCTACGTTTACAAAGCACTGGGCATAGTTTCTCCACGAGTGCTTTCTAGTCGCACTTTTTAAAAAAGTGTGGTAACATGTCCATTTTGGTTTCGCCAAGTAACAACACGGGCTGGGGACTGGGTGCCGCCCGCATGTATGGAGGAGCTAAAACAAGGTCTAGCCAACATCCAGTGCGCGTACGCGGACATTACCGAGCTCCATGGGGCGCGCATACCCGAGGACGCACTGGTCGCACCACTGTAGACGATGTTATTGACTCGGTAGTGGCCGATGCTCGCAAGTACCGCGCGCCCGCTGAAACAGCAGGGTCTACTGTTGATGCAGTAATTGATGAGGTAGTGGCAAACGCGCGGGCTTATGCAAGGCGCCGCAGACGGCTGCGTCGCCGGCGTAGACCAACCACCGCCATGCGCGCGGCCAGAGCGTTGGTTCGACGGGCCAGGCGCATTGGGCGGCGAGCTATGATGCGGGCAGCCAGGCGGGCTGCAACGCCTGCCGGTCGAGCGCGGAGACGGGCCGCAGCTGCGGCCGCAACAGCTATTGCAAACCTAGCTGCTCCGCGACGAGGAAATGTATACTGGGTGCGCGACTCAGTGACCGGGACGCGTGTGCCAGTTCGTACGCGTCCACCTCACCCTTAGAAGACAAAGAGTGACTCAATGTCTGTTATGTATGCCCAGCATGACCAAACGCAAGTTCAAAGAAGAGCTGCTGCAGGCCTTAGCGCCTGAAATATATGGCCCATCGGATAACCTTACCAAGCGCGATATCAAGCATGTTAAAAAACGGGAAAAAAAAGAGGAAGAAGTCGCCGCGGCGTCAGCAGACGGCGTCGAGTTTGTGCGCTCATTTGCGCCCAGACGTAGGGTACAGTGGAAGGGACGGCAAGTAAAACGCATTTTGCGACCGGGCACCACAGTGGTTTTTTCTCCCGGAGAGCGAACGATTATGCGTCCCCTAAAGCGCGAGTACGACGAAGTGTACGCAGACGATGACATTTTGGAGCAAGCGGCACAACAGACTGGGGAATTTGCATATGGAAAAAAAGGGCGTTACGGAGACAAAATTGCTATTCCTTTGGACGAGGGAAATCCAACACCCAGTTTAAAGGCTGTCACTTTGCAACAAGTGTTGCCCGTCCTTGGGCCTTCGGAAGAAAAGCGTGGAATTAAAAGGGAAGCCATGGATGAATTGCAGCCTACAATGCAACTGATGGTGCCTAAGCGGCAAAAGTTAGAGGACGTACTAGAGCACATGAAGGTGGATCCTAGCGTACAGCCAGATGTAAAAGTACGTCCGATAAAAAAGGTAGCTCCAGGATTGGGAGTTCAAACAGTGGACATTCAAATTCCTGTGCAAACTGCATTGGGTGAAACTATGGAAATCCAAACTTCGCCAATAAAAACAACGGTGAACGCAAGCGTGCAAACAGACCCTTGGTACCCGCCAGTGCTTTCAACAAAAAAAAAGCGTCACTACAGACAAACAAGTTCGCTTTTGCCAGACTACGTTTTACATCCTTCCATTGTGCCCACGCCTGGGTACCGTGGGACAACTTTTCAGCGCCGAGCCACAGCCCCTAGCCGTAGACGAGGTCCATCACGCCGTAGACGTCGACGCAAAGCCACTTTAGCCCCAGCGGCAGTACGTCGCGTTGTACAAAGGGGGCGCACACTAATACTTCCATCCGTGCGTTACCACCCTAGCATTCTCTAACAAGCTGCGCTGCCGTTTTTTCAGATGGCTCTTACTTGCCGAATGCGCATACCCATTCCAGGATACAGAGGACGACCCCGCCGGAGGAAAGGGCTGACCGGGAACGGTCGATTTCGGCGGCGTAGTATGCGCAGACGCATGAAGGGTGGGGTGCTGCCCTTCCTAATTCCACTTATTGCTGCGGCCATTGGAGCCGTTCCCGGAATTGCCTCAGTAGCCTTGCAGGCTTCTCGAAAAAATTAAAATAAAATAAAACTTCCAACTTATTACTGGTACTATGACTGTTTTATGCAGAATCGATATCTTTCTAGAAGATCTCCTACAATATTCTCAGCTGCCATGGAAAATCGATGTTCTTCTTTTATTCTCTCAAGATTTTCAGGCTGTATATTAAAACTTATATTAAGAACTATGCTAACCACCTCATCAGGAACCGTTGTAGGTGGCGTGGGTTTTCTTGGCAATCGACTCTCATGAAAACTACGAGCTAAATATTCAATATGTTCCTCTTGACCAACTTTATTCTGCATTTTTTTTGAACGAGGTTTAGAGCAAGCTTCAGGAAACTGAGACAGGAATTTTATTAAAAATTTAAATTTTGAAGAAAGTTCAGGGTTAATAGCATCCATTTTTTGCTTTGCAAGTTCCTCAGCATTCTTAACAAAAGACGTCTCTTTTGACATGTTTAAAGTTTAAACCTCCTGTGTGAAATTATTATCCGCTCATAATTCCACACATTATACGAGCCGGAAGCATAAAGTGTAAAGCCTGGGGTGCCTAATGAGTGAGCTAACTCACATTAATTGCGTTGCGCTCACTGCCAATTGCTTTCCAGTCGGGAAACCTGTCGTGCCAGCTGCATTAATGAATCGGCCAACGCGCGGGGAGAGGCGGTTTGCGTATTGGGCGCTCTTCCGCTTCCTCGCTCACTGACTCGCTGCGCTCGGTCGTTCGGCTGCGGCGAGCGGTATCAGCTCACTCAAAGGCGGTAATACGGTTATCCACAGAATCAGGGGATAACGCAGGAAAGAACATGTGAGCAAAAGGCCAGCAAAAGGCCAGGAACCGTAAAAAGGCCGCGTTGCTGGCGTTTTTCCATAGGCTCCGCCCCCCTGACGAGCATCACAAAAATCGACGCTCAAGTCAGAGGTGGCGAAACCCGACAGGACTATAAAGATACCAGGCGTTTCCCCCTGGAAGCTCCCTCGTGCGCTCTCCTGTTCCGACCCTGCCGCTTACCGGATACCTGTCCGCCTTTCTCCCTTCGGGAAGCGTGGCGCTTTCTCATAGCTCACGCTGTAGGTATCTCAGTTCGGTGTAGGTCGTTCGCTCCAAGCTGGGCTGTGTGCACGAACCCCCCGTTCAGCCCGACCGCTGCGCCTTATCCGGTAACTATCGTCTTGAGTCCAACCCGGTAAGACACGACTTATCGCCACTGGCAGCAGCCACTGGTAACAGGATTAGCAGAGCGAGGTATGTAGGCGGTGCTACAGAGTTCTTGAAGTGGTGGCCTAACTACGGCTACACTAGAAGAACAGTATTTGGTATCTGCGCTCTGCTGAAGCCAGTTACCTTCGGAAAAAGAGTTGGTAGCTCTTGATCCGGCAAACAAACCACCGCTGGTAGCGGTGGTTTTTTTGTTTGCAAGCAGCAGATTACGCGCAGAAAAAAAGGATCTCAAGAAGATCCTTTGATCTTTTCTACGGGGTCTGACGCTCAGTGGAACGAAAACTCACGTTAAGGGATTTTGGTCATGAGATTATCAAAAAGGATCTTCACCTAGATCCTTTTAAATTAAAAATGAAGTTTTAAATCAATCTAAAGTATATATGAGTAAACTTGGTCTGACAGTTACCAATGCTTAATCAGTGAGGCACCTATCTCAGCGATCTGTCTATTTCGTTCATCCATAGTTGCCTGACTCCCCGTCGTGTAGATAACTACGATACGGGAGGGCTTACCATCTGGCCCCAGTGCTGCAATGATACCGCGAGACCCACGCTCACCGGCTCCAGATTTATCAGCAATAAACCAGCCAGCCGGAAGGGCCGAGCGCAGAAGTGGTCCTGCAACTTTATCCGCCTCCATCCAGTCTATTAATTGTTGCCGGGAAGCTAGAGTAAGTAGTTCGCCAGTTAATAGTTTGCGCAACGTTGTTGCCATTGCTACAGGCATCGTGGTGTCACGCTCGTCGTTTGGTATGGCTTCATTCAGCTCCGGTTCCCAACGATCAAGGCGAGTTACATGATCCCCCATGTTGTGCAAAAAAGCGGTTAGCTCCTTCGGTCCTCCGATCGTTGTCAGAAGTAAGTTGGCCGCAGTGTTATCACTCATGGTTATGGCAGCACTGCATAATTCTCTTACTGTCATGCCATCCGTAAGATGCTTTTCTGTGACTGGTGAGTACTCAACCAAGTCATTCTGAGAATAGTGTATGCGGCGACCGAGTTGCTCTTGCCCGGCGTCAATACGGGATAATACCGCGCCACATAGCAGAACTTTAAAAGTGCTCATCATTGGAAAACGTTCTTCGGGGCGAAAACTCTCAAGGATCTTACCGCTGTTGAGATCCAGTTCGATGTAACCCACTCGTGCACCCAACTGATCTTCAGCATCTTTTACTTTCACCAGCGTTTCTGGGTGAGCAAAAACAGGAAGGCAAAATGCCGCAAAAAAGGGAATAAGGGCGACACGGAAATGTTGAATACTCATACTCTTCCTTTTTCAATATTATTGAAGCATTTATCAGGGTTATTGTCTCATGAGCGGATACATATTTGAATGTATTTAGAAAAATAAACAAATAGGGGTTCCGCGCACATTTCCCCGAAAAGTGCCACCTGACGTCTAAGAAACCATTATTATCATGACATTAACCTATAAAAATAGGCGTATCACGAGGCCGCCCCTGCAGCCGAATTATATTATTTTTGCCAAATAATTTTTAACAAAAGCTCTGAAGTCTTCTTCATTTAAATTCTTAGATGATACTTCATCTGGAAAATTGTCCCAATTAGTAGCATCACGCTGTGAGTAAGTTCTAAACCATTTTTTTATTGTTGTATTATCTCTAATCTTACTACTCGATGAGTTTTCGGTATTATCTCTATTTTTAACTTGGAGCAGGTTCCATTCATTGTTTTTTTCATCATAGTGAATAAAATCAACTGCTTTAACACTTGTGCCTGAACACCATATCCATCCGGCG

>Bloc 4

CTGCTGAAGCCAGTTACCTTCGGAAAAAGAGTTGGTAGCTCTTGATCCGGCAAACAAACCACCGCTGGTAGCGGTGGTTTTTTTGTTTGCAAGCAGCAGATTACGCGCAGAAAAAAAGGATCTCAAGAAGATCCTTTGATCTTTTCTACGGGGTCTGACGCTCAGTGGAACGAAAACTCACGTTAAGGGATTTTGGTCATGAGATTATCAAAAAGGATCTTCACCTAGATCCTTTTAAATTAAAAATGAAGTTTTAAATCAATCTAAAGTATATATGAGTAAACTTGGTCTGACAGTTACCAATGCTTAATCAGTGAGGCACCTATCTCAGCGATCTGTCTATTTCGTTCATCCATAGTTGCCTGACTCCCCGTCGTGTAGATAACTACGATACGGGAGGGCTTACCATCTGGCCCCAGTGCTGCAATGATACCGCGAGACCCACGCTCACCGGCTCCAGATTTATCAGCAATAAACCAGCCAGCCGGAAGGGCCGAGCGCAGAAGTGGTCCTGCAACTTTATCCGCCTCCATCCAGTCTATTAATTGTTGCCGGGAAGCTAGAGTAAGTAGTTCGCCAGTTAATAGTTTGCGCAACGTTGTTGCCATTGCTACAGGCATCGTGGTGTCACGCTCGTCGTTTGGTATGGCTTCATTCAGCTCCGGTTCCCAACGATCAAGGCGAGTTACATGATCCCCCATGTTGTGCAAAAAAGCGGTTAGCTCCTTCGGTCCTCCGATCGTTGTCAGAAGTAAGTTGGCCGCAGTGTTATCACTCATGGTTATGGCAGCACTGCATAATTCTCTTACTGTCATGCCATCCGTAAGATGCTTTTCTGTGACTGGTGAGTACTCAACCAAGTCATTCTGAGAATAGTGTATGCGGCGACCGAGTTGCTCTTGCCCGGCGTCAATACGGGATAATACCGCGCCACATAGCAGAACTTTAAAAGTGCTCATCATTGGAAAACGTTCTTCGGGGCGAAAACTCTCAAGGATCTTACCGCTGTTGAGATCCAGTTCGATGTAACCCACTCGTGCACCCAACTGATCTTCAGCATCTTTTACTTTCACCAGCGTTTCTGGGTGAGCAAAAACAGGAAGGCAAAATGCCGCAAAAAAGGGAATAAGGGCGACACGGAAATGTTGAATACTCATACTCTTCCTTTTTCAATATTATTGAAGCATTTATCAGGGTTATTGTCTCATGAGCGGATACATATTTGAATGTATTTAGAAAAATAAACAAATAGGGGTTCCGCGCACATTTCCCCGAAAAGTGCCACCTGACGTCTAAGAAACCATTATTATCATGACATTAACCTATAAAAATAGGCGTATCACGAGGCCGCCCCTGCAGCCGAATTATATTATTTTTGCCAAATAATTTTTAACAAAAGCTCTGAAGTCTTCTTCATTTAAATTCTTAGATGATACTTCATCTGGAAAATTGTCCCAATTAGTAGCATCACGCTGTGAGTAAGTTCTAAACCATTTTTTTATTGTTGTATTATCTCTAATCTTACTACTCGATGAGTTTTCGGTATTATCTCTATTTTTAACTTGGAGCAGGTTCCATTCATTGTTTTTTTCATCATAGTGAATAAAATCAACTGCTTTAACACTTGTGCCTGAACACCATATCCATCCGGCGTAATACGACTCACTATAGGGAGAGCGGCCGCCAGATCTTCCGGATGGCTCGAGTTTTTCAGCAAGATATCGATTACTGGTACTATGACTGTTTTATGCAGACTAAATGGAAGACATCAATTTTTCGTCGCTGGCCCCGCGACACGGCACGCGGCCGTACATGGGCACCTGGAACGAGATCGGCACGAGCCAGCTGAACGGGGGCGCCTTCAATTGGAACAGTATCTGGAGCGGTCTTAAAAATTTTGGTTCCACGATTAAGACATATGGCACCAAGGCGTGGAACAGCCAAACCGGCCAGATGCTAAGGGACAAGTTAAAAGACCAAAATTTTCAACAGAAAGTTGTAGATGGTCTGGCTTCGGGAATTAATGGAGTTGTAGACATAGCCAATCAGGCTGTACAGAAAAAAATTGCCAACCGTTTAGAGCCGCGGCCCGACGAGGTAATGGTAGAGGAAAAGCTGCCACCTCTAGAAACTGTGCCCGGATCCGTTCCAACCAAAGGAGAAAAGCGGCCACGGCCGGATGCAGAGGAAACCTTAGTAACGCACACAACAGAACCGCCGTCCTATGAGGAAGCAATAAAACAAGGAGCCGCTCTGTCACCTACCACCTATCCCATGACCAAGCCTATTTTACCCATGGCTACTAGAGTGTATGGAAAAAACGAAAATGTGCCTATGACCCTTGAGCTGCCTCCTTTGCCAGAACCCACTATCGCGGATCCCGTAGGTTCCGTTCCTGTTGCATCTGTTCCAGTTGCATCGACAGTGAGCCGTCCAGCAGTGCGGCCTGTTGCCGTGGCTAGCTTGCGAAACCCACGATCCAGTAATTGGCAAAGTACCCTAAACAGTATTGTGGGACTGGGAGTAAAGTCTCTCAAACGCCGACGCTGCTACTAACATTAAAAGACGAGTGTTAATTCCCATCTGTGTATACGCCTCCTATGTTAGCGCCAGAGGACCAACGCGTGAATCGCAGTCACCACCAGCGCTTTCAAGATGGCCACTCCCTCGATGATGCCGCAGTGGTCTTACATGCACATCGCCGGTCAGGATGCCTCGGAGTACCTGAGTCCCGGTCTGGTGCAATTCGCCCGCGCCACGGACACCTACTTCACCCTGGGAAACAAGTTTAGAAACCCCACCGTGGCTCCCACCCATGATGTTACCACCGATCGCTCGCAGCGTCTGACGCTGCGTTTTGTGCCCGTGGATCGGGAAGATACTACCTACTCCTACAAGGCTCGCTTTACGCTGGCTGTGGGTGACAACCGCGTGTTAGACATGGCTAGTTCTTACTTTGACATTCGAGGGGTACTGGATCGTGGTCCCAGTTTTAAGCCCTATTCCGGAACCGCCTACAATTCTTTGGCACCAAAAGGCGCTCCTAATGCTTCACAATGGTCAGATAACGCTAAGCTTAATACCTTTGCTCAGGCGCCGTATCTTAGCGACACTATCACCGCCGCCGATGGTATTAAAGTTGGAACAGACACCGCCCAGGCAGGCGCGGCGGTGTATGCCAACAAAACTTATCAGCCAGAGCCGCAAGTAGGACCAAGTGAATGGAACACCAGCATTGAAAACGTTAAAGCTGGCGGGAGGGCATTAAAGCAAACCACTGCAATGCAGCCGTGCTATGGCTCCTACGCTCGTCCAACCAACGAACACGGAGGACAATCCAAGGATGACAACATTGAACTTAAGTTCTTTGATTCAGCTAACAATGCAGCAAACACTGCTCAAGTTGTGTTCTATACCGAAGACGTAAACCTTGAAATGCCAGACACGCATCTTGTGTTTAAGCCTACTGTTACCAATGGAACAATTGCTTCTGAGTCGCTGTTGGGACAGCAAGCAGCGCCAAATAGAGCAAACTACATTGCATTCAGAGATAATTTTATTGGCCTGATGTATTACAACAGTACAGGCAACATGGGTGTATTGGCCGGGCAAGCTTCCCAACTTAACGCAGTAGTAGACCTGCAAGACAGAAATACAGAGCTGTCATACCAGTTAATGCTGGATGCTTTGGGAGACAGAACACGGTACTTTTCCTTGTGGAATTCCGCAGTGGACAGTTACGACCCTGACGTTCGCGTTATTGAGAATCACGGGGTAGAGGATGAACTACCAAATTATTGCTTTCCTCTTAGCGCAGTAGGTGAAATAAAAAATTACAAAGGCATTAAGCCAGATAACGGAGGAGGAGGTGGCTGGACTGCCGACAACACTGTCAGTGAAGCAAACCACATAGGCATTGGGAATATAGCCGCCATGGAAATTAATTTGCAGGCTAATTTGTGGAGAAGCTTCTTGTACTCAAATGTGGGCTTATACCTACCAGACGACTTAAAATACACTCCAGGAAACATAAAACTACCTGATAACAAGAACACCTACGAGTACATGAACGGGCGTGTGACTGCCCCGGGGTTGGTGGATACCTATGTCAATATCGGCGCTCGCTGGTCCCCAGATGTGATGGATAATGTAAACCCTTTTAACCACCACCGAAACGCAGGGTTGCGCTACAGATCCATGTTGCTAGGCAATGGGAGATTTGTTCCTTTTCACATTCAGGTGCCGCAAAAATTTTTTGCCATCAGAAATTTGTTGCTGTTGCCCGGTTCCTACACTTACGAATGGAACTTTAGAAAGGATGTAAACATGATTCTTCAGAGCACACTGGGAAATGATCTTCGGGTGGACGGAGCCAGCGTTCGCTTTGACAACATTGCCCTGTATGCTAACTTTTTTCCCATGGCACATAACACAGCTTCTACTTTAGAAGCCATGTTAAGAAATGACACCAACGACCAGTCTTTTAACGATTATTTGTGTGCTGCAAACATGCTGTATCCCATCCCAGCTAACGCCACCAGCGTGCCCATTTCAATACCTTCGCGAAATTGGGCGGCATTTAGAGGCTGGAGCTTTACTCGCCTAAAAACTAAAGAAACTCCTTCCCTGGGTTCAGGGTTTGACCCCTACTTTGTATACTCTGGAACCATTCCCTATTTAGACGGCACCTTTTACCTAAACCACACTTTTAAGAAGGTGTCAATCATGTTTGACTCCTCCGTGAGTTGGCCTGGAAATGACCGTTTGCTAACCCCAAATGAATTTGAAATAAAGCGTTCTGTGGATGGGGAGGGATACAATGTGGCCCAATGCAATATGACTAAGGATTGGTTCCTAATACAAATGCTTAGTCATTACAACATTGGATACCAAGGTTTTTACATTCCAGAGAGCTACAAGGACCGCATGTATTCTTTCTTTAGAAACTTTCAGCCCATGAGTAGGCAAGTTGTGGATACCACAGAATATAAGAACTACAAAAAAGTAACCGTAGAGTTTCAACATAACAACTCAGGATTCGTGGGATACCTGGGCCCCACTATGCGGGAGGGACAAGCTTACCCCGCCAACTATCCCTACCCTCTTATAGGCAAAACAGCTGTGGAAAGCATCACACAGAAAAAGTTTCTATGCGATCGTGTTATGTGGCGCATCCCATTTTCTAGTAACTTCATGTCTATGGGGGCGCTAACGGATCTTGGGCAAAATATGCTGTACGCAAACTCAGCCCATGCTCTAGACATGACATTTGAGGTGGATCCAATGGATGAGCCTACCCTTCTTTATGTTTTATTTGAAGTTTTCGACGTGGTACGCATTCACCAGCCACACCGCGGCGTCATTGAAGCGGTCTACCTGCGCACGCCCTTCTCGGCGGGTAACGCTACCACCTAAGAAGGCACCCTCCCAGACTGCTGTAATGGGTTCAAGCGAACAGGAGCTGACGGCCATTGTTCGAGATCTAGGCTGTGGACCCTATTTTTTGGGAACCTTTGACAAACGTTTTCCGGGTTTTGTGTCTCGCGACCGCTTATCATGTGCTATTGTTAACACTGCCGGTCGCGAAACTGGGGGCGTACACTGGCTGGCTTTTGGATGGAACCCCAAATCGCACACTTGCTATTTATTCGATCCATTTGGATTTTCTGATCAACGACTAAAACAAATCTATCAGTTTGAGTACGAAAGTCTGTTGCGCCGTAGTGCGCTAGCGGCCACTAAAGACCGATGCGTTACCCTAGAAAAGTCAACCCAAACTGTACAAGGACCGTTTTCTGCAGCGTGCGGCCTGTTTTGTTGTATGTTCTTACACGCTTTTACTCACTGGCCTGACCATCCAATGGATAAAAATCCCACTATGGACCTACTTACTGGGGTGCCTAATTGTATGCTACAAAGTCCTCAGGTAGTGGGCACATTGCAACGCAATCAGAATGAATTGTATAAATTCTTAAACAATCTGTCCCCTTACTTTCGTCACAACCGCGAGCGCATAGAAAAAGCTACATCTTTTACTAAAATGCAAAATGGACTCAAATAAACGTGTACACAATGCATTAATAATAAAACCATTTTATTAGCTCATTGGAGTACAAGCTTATCGATATCTTTCTAGAAGATCTCCTACAATATTCTCAGCTGCCATGGAAAATCGATGTTCTTCTTTTATTCTCTCAAGATTTTCAGGCTGTATATTAAAACTTATATTAAGAACTATGCTAACCACCTCATCAGGAACCGTTGTAGGTGGCGTGGGTTTTCTTGGCAATCGACTCTCATGAAAACTACGAGCTAAATATTCAATATGTTCCTCTTGACCAACTTTATTCTGCATTTTTTTTGAACGAGGTTTAGAGCAAGCTTCAGGAAACTGAGACAGGAATTTTATTAAAAATTTAAATTTTGAAGAAAGTTCAGGGTTAATAGCATCCATTTTTTGCTTTGCAAGTTCCTCAGCATTCTTAACAAAAGACGTCTCTTTTGACATGTTTAAAGTTTAAACCTCCTGTGTGAAATTATTATCCGCTCATAATTCCACACATTATACGAGCCGGAAGCATAAAGTGTAAAGCCTGGGGTGCCTAATGAGTGAGCTAACTCACATTAATTGCGTTGCGCTCACTGCCAATTGCTTTCCAGTCGGGAAACCTGTCGTGCCAGCTGCATTAATGAATCGGCCAACGCGCGGGGAGAGGCGGTTTGCGTATTGGGCGCTCTTCCGCTTCCTCGCTCACTGACTCGCTGCGCTCGGTCGTTCGGCTGCGGCGAGCGGTATCAGCTCACTCAAAGGCGGTAATACGGTTATCCACAGAATCAGGGGATAACGCAGGAAAGAACATGTGAGCAAAAGGCCAGCAAAAGGCCAGGAACCGTAAAAAGGCCGCGTTGCTGGCGTTTTTCCATAGGCTCCGCCCCCCTGACGAGCATCACAAAAATCGACGCTCAAGTCAGAGGTGGCGAAACCCGACAGGACTATAAAGATACCAGGCGTTTCCCCCTGGAAGCTCCCTCGTGCGCTCTCCTGTTCCGACCCTGCCGCTTACCGGATACCTGTCCGCCTTTCTCCCTTCGGGAAGCGTGGCGCTTTCTCATAGCTCACGCTGTAGGTATCTCAGTTCGGTGTAGGTCGTTCGCTCCAAGCTGGGCTGTGTGCACGAACCCCCCGTTCAGCCCGACCGCTGCGCCTTATCCGGTAACTATCGTCTTGAGTCCAACCCGGTAAGACACGACTTATCGCCACTGGCAGCAGCCACTGGTAACAGGATTAGCAGAGCGAGGTATGTAGGCGGTGCTACAGAGTTCTTGAAGTGGTGGCCTAACTACGGCTACACTAGAAGAACAGTATTTGGTATCTGCGCT

>Bloc 5

ACTCTTCCTTTTTCAATATTATTGAAGCATTTATCAGGGTTATTGTCTCATGAGCGGATACATATTTGAATGTATTTAGAAAAATAAACAAATAGGGGTTCCGCGCACATTTCCCCGAAAAGTGCCACCTGACGTCTAAGAAACCATTATTATCATGACATTAACCTATAAAAATAGGCGTATCACGAGGCCGCCCCTGCAGCCGAATTATATTATTTTTGCCAAATAATTTTTAACAAAAGCTCTGAAGTCTTCTTCATTTAAATTCTTAGATGATACTTCATCTGGAAAATTGTCCCAATTAGTAGCATCACGCTGTGAGTAAGTTCTAAACCATTTTTTTATTGTTGTATTATCTCTAATCTTACTACTCGATGAGTTTTCGGTATTATCTCTATTTTTAACTTGGAGCAGGTTCCATTCATTGTTTTTTTCATCATAGTGAATAAAATCAACTGCTTTAACACTTGTGCCTGAACACCATATCCATCCGGCGTAATACGACTCACTATAGGGAGAGCGGCCGCCAGATCTTCCGGATGGCTCGAGTTTTTCAGCAAGATATCGATTAGCTCATTGGAGTACAAGCTTGACTGTTTTATTAAAAATCAAATGGCTCTTCGCGACAGTCGCCGTGGTTGGTGGGCAGGGATATGTTTCTGTACTGCAAACGCTGATGCCACTTGAATTCTGGAATAACAAGCCTAGGGGGGGAGCCGTCAAAATTTTCTCCCCACAGCTGGCGCACAAGTTGCAGGGCGCCCATAACATCAGGAGCAGAAATCTTGAAGTCGCAATTAGGGCCAGCATTGCCGCGCGCATTGCGATAAACTGGATTTGCGCACTGAAAAACCAACAAACACGGATACTTAATACTGGCTAACGCTCCAGGGTCGGTTACTTCGTTGATATCAATGTTATCCACATTGCTGAGGTTAAAAGGAGTGATTTTACACAGTTGACGCCCCATCCGTGGCAGGCCATCTTGCTTGTTTAAACATTCGCAGCGCACTGGCATAAGGAGACGTTTTTGCCCATGTCGCATGTGAGGGTAGTCGGCCAGCATAAAAGCTTCAATTTGCCTAAAAGCTATTTGAGCCTTCATTCCTTCAGAATAAAACAAGCCGCAGGACTTTCCGGAGAAAGAATTATTCCCGCAGCCAACATCATGAAAACAGCAGCGGGCATCGTCGTTTTTAATTTGAACTACATTACGCCCCCAGCGGTTTTGCGCCACCTTGGCTTTCGAGGGGTTCTCTTTCAACGCTCGTTGCCCACTTTCGCTGGTTACATCCATTTCCACCAAATGCTCTTTGCGCACCATCTCCATTCCATGCAGGCATCTAAGCTCCCCTTCGCGCTCGGTACACTTATGCTCCCACACGCAGCAACCGGTGGGTTCCCAGGAATTCTGTTGGACACCGGCATAAGCTTGCATATATCCTTGCAAAAAGCGTCCCATGAGCTCCTGAAAGGTTTTTTGGGATGAAAAAGTCAGCTGCAAACCGCGCTTTTCTTCGTTGAGCCATGTTGTGCATATTTTCTTGTACACGCTGCCCTGATCCGGCAAAAAACGAAAGGTGGCGCGCTCGTCGTGATCCACATGGTACTTTTCCATTAGCATAGCCATGGCTTCCATGCCTTTTTCCCAAGCTGAAACTAGGGGCTGGCTTGCCGGATTGCGAACAACAACAACATTCTTTTCATTTTCGTCGCTGTTTTGAGCGGAAGCCTTCAAAACGTGTACCTGCCTGGTTTCCATTTTTTGAAAAGACTGAGAACCGTCTGCATGATGCATAATGCGGACGGGCGGCATGCTGAAACCCATTACTCCTAAAACTGCTCTTGGTGGTTCTGCCTCTTCTTCTTCTGCACTCTCTGGGGAAAGAGGTATCGCAGCCATAGATTTCTTGACTTTTTTCTTTGGAGGTAAAGGCACAGCTTCCAGTTCTTCTTCGCTTTCGGAATCCAGAAAGTATCTGCCCATTTTTGGCGGCGGCGGCTGAGCGCTGCGGTCTGGGGTGCGCTCCCTCTGTGAGTGCTGATTGCTGGCCATTATTTAATCCTAGGCAAAGAAACACATGATGGATCTGGAGCCACAGGAAAGCTTAACCGCCCCCACCGCTCCCGCCATTGGCGCTACGGCTGTCATGGAGAAGGACAAAAGTCTACTCATACCCCAAGACGCACCGGTTGAGCAGAACTTGGGCTACGAGACTCCCCCCGAGGAATTTGAAGGCTTTCTTCAAATCCAAAAGCAACCAAATGAGCAAAACGCTGGGCTCGAGGACCATGACTACCTAAACGAGGGAGATGTCCTGTTTAAACATCTACAGCGACAAAGCACTATCGTTCGCGACGCCATATCTGATCGCTCTTCAATACCAGTTTCAATTGCAGAACTATCTTGCATCTACGAACGCAACCTGTTCTCCCCACGTGTGCCCCCTAAACGGCAAGCCAACGGCACATGCGAGCCAAATCCTCGCCTTAACTTCTACCCAGTTTTTGCAGTGCCAGAAGCACTGGCAACATACCATATTTTCTTTAAAAATCACAAAATACCCCTATCCTGTCGAGCTAACCGCAGCCGCGCAGATGAGCTTCTTGCTTTAAGGGCTGGCGCTTCCATACCTGGGATTGTGTCCTTGGAAGAGGTGCCTAAAATTTTTGAAGGTTTAGGTCGGGATGAAAAACGAGCAGCAAATGCCCTGCAAAAAGAAAATGAACAAAATCACCATGGGAATAGTGCTCTAATAGAACTGGAAGGTGACAATGCCCGCCTGGCAGTTTTAAAGCGCAATATTGAGGTTACTCACTTTGCCTACCCGGCAGTAAATCTTCCGCCAAAGGTAATGAGCGCAGTGATGAATCAGCTACTAATTAAGCGAGCCCAACCCATTGACAAAGATGCAAACTTGCAAGACCCGGAGGCAACAGATGATGGAAAGCCGGTTGTAAGCGACGAGCAATTAACTAAGTGGTTGGGAACAGACAATTCCAACGAACTACAACAGCGGCGTAAACTCATGATGGCCGCCGTACTTGTAACTGTGGAACTCGAGTGCATGCATCGTTTTTTCTCCGACATCACCACATTGCGCAAAATTGAGGAATGTCTTCACTACACTTTCCGCCATGGCTACGTGCGCCAAGCCTGTAAAATTTCTAATGTGGAGCTGAGCAATCTAGTTTCTTACATGGGCATCTTGCATGAAAACCGATTGGGACAGAACGTGCTACACTCAACACTACGCGATGAAGCACGCAGAGATTACGTGCGAGACTGCATTTACCTTTTCCTGTTACATACCTGGCAAACTGGGATGGGTGTTTGGCAGCAATGCTTGGAAGAAAAAAACCTTCGAGAACTAAACAAACTGTTAGACAGAGCACTAAAATCCCTATGGACCGGTTTTGACGAACGGACAGTAGCTGCAGAGCTAGCTGACATAATTTTCCCAGAAAGGTTAATGATAACCTTGCAAAACGGCTTGCCTGACTTTATGAGTCAAAGTATGCTGCACAATTATCGCTCTTTTATATTAGAGCGTTCTGGGATGCTTCCTAGCATGTGTTGTGCACTTCCTTCAGATTTTGTGCCTATATATTTTAGAGAGTGCCCCCCTCCCCTGTGGAGCCACTGCTACTTACTACGACTTGCTAACTACCTAGCTTACCACTCAGACCTTATGACAGATTCAAGCGGCGAAGGCCTAATGGAGTGTCACTGCCGCTGCAATCTTTGCACCCCCCACCGTTCTTTGGTTTGCAATACTGAACTATTAAGTGAAAGTCAAGTCATTGGTACCTTCGAAATGCAGGGACCGCAGTCTGACAGCAATTTCACGACGAACCTAAGACTTACCCCTGGGCTTTGGACTTCTGCCTACCTGCGCAAATTTGAACCCCAAGATTACCACGCCCACAGTATCAATTTTTACGAAGACCAATCCAAACCCCCAAAAGCGCCACTAACGGCTTGCGTCATTACGCAGGGAAAAATTCTAGCCCAATTGCATGCTATTAAGCAAGCGCGCGAAGAGTTTTTACTTAAAAAAGGACACGGAGTGTACCTTGATCCCCAAACCGGCGAGGAACTAAACCTTCCATCACCTTTGTGTGCTACTGCGTCTCCCCATTCGCAGCATGTCCCCGAAAGCCGCAAAACAGGCTATTGCGCAGCAACGCTCAAAGAAACAGCAGCAACGGCAGGAAATCTGGGAGGAAGAATCTTGGGAGAGTCAGGCAGAGGACGAGGTCGAGGACTTGGAAGAATGGGAGGAGGAGGAGGCGGACAGCCTAGACGAGGATCCAGAGGAGGAGGAGGAAGGTTCCAAGGACGGAGCGACCGCCGCAAAACCGTCGCTTTCAACCAAGCCCTCTCCAATGAAACCCGCTGTGAGCAAATCTCAGAAAGCCAGCCGTAGATGGGACACCATTGAAACCAGCGCCGCAAACTTGGGTAAGAATCGCAAGCAGGCGCGTCGGGGCTACTGCTCATGGCGGGCTCACAAAAGTAATATTGTAGCCTGCTTGCAGCACTGCGGGGGGAATATCTCATTTGCAAGGCGGTATTTGCTATACCATGATGGAGTGGCGATTCCAAGGAATGTCCTCCATTACTACCGTCATCTCTACAGCCCCTTTGAAGAGCTCGACAAGGAACCGACCTGCAACAGCCAAGCGGCCCACTAGAATCGGCAACAGCAGCAACAAGGAAAGTCCTGAGGCGCGCGAGTTAAGAAAACGCATTTTTCCCACTTTATATGCTATTTTTCAGCAGAGTCGAGGTCAAGAACACGAACTGAAAATAAAAAACCGTTCCCTGCGTTCACTTACCCGCAGCTGTCTCTACCTCAAAAGCGAAGATCAGTTGCAACGCACCTTGCAGGACGCAGAAGCTCTGTTCAATAAATACTGCTCCCTCTCGCTTAAAGAGTAAAAAAAGCCCGCGCGCGGACTTTCAACAGGCGGGAAAAGTGAATCGATATCTTTCTAGAAGATCTCCTACAATATTCTCAGCTGCCATGGAAAATCGATGTTCTTCTTTTATTCTCTCAAGATTTTCAGGCTGTATATTAAAACTTATATTAAGAACTATGCTAACCACCTCATCAGGAACCGTTGTAGGTGGCGTGGGTTTTCTTGGCAATCGACTCTCATGAAAACTACGAGCTAAATATTCAATATGTTCCTCTTGACCAACTTTATTCTGCATTTTTTTTGAACGAGGTTTAGAGCAAGCTTCAGGAAACTGAGACAGGAATTTTATTAAAAATTTAAATTTTGAAGAAAGTTCAGGGTTAATAGCATCCATTTTTTGCTTTGCAAGTTCCTCAGCATTCTTAACAAAAGACGTCTCTTTTGACATGTTTAAAGTTTAAACCTCCTGTGTGAAATTATTATCCGCTCATAATTCCACACATTATACGAGCCGGAAGCATAAAGTGTAAAGCCTGGGGTGCCTAATGAGTGAGCTAACTCACATTAATTGCGTTGCGCTCACTGCCAATTGCTTTCCAGTCGGGAAACCTGTCGTGCCAGCTGCATTAATGAATCGGCCAACGCGCGGGGAGAGGCGGTTTGCGTATTGGGCGCTCTTCCGCTTCCTCGCTCACTGACTCGCTGCGCTCGGTCGTTCGGCTGCGGCGAGCGGTATCAGCTCACTCAAAGGCGGTAATACGGTTATCCACAGAATCAGGGGATAACGCAGGAAAGAACATGTGAGCAAAAGGCCAGCAAAAGGCCAGGAACCGTAAAAAGGCCGCGTTGCTGGCGTTTTTCCATAGGCTCCGCCCCCCTGACGAGCATCACAAAAATCGACGCTCAAGTCAGAGGTGGCGAAACCCGACAGGACTATAAAGATACCAGGCGTTTCCCCCTGGAAGCTCCCTCGTGCGCTCTCCTGTTCCGACCCTGCCGCTTACCGGATACCTGTCCGCCTTTCTCCCTTCGGGAAGCGTGGCGCTTTCTCATAGCTCACGCTGTAGGTATCTCAGTTCGGTGTAGGTCGTTCGCTCCAAGCTGGGCTGTGTGCACGAACCCCCCGTTCAGCCCGACCGCTGCGCCTTATCCGGTAACTATCGTCTTGAGTCCAACCCGGTAAGACACGACTTATCGCCACTGGCAGCAGCCACTGGTAACAGGATTAGCAGAGCGAGGTATGTAGGCGGTGCTACAGAGTTCTTGAAGTGGTGGCCTAACTACGGCTACACTAGAAGAACAGTATTTGGTATCTGCGCTCTGCTGAAGCCAGTTACCTTCGGAAAAAGAGTTGGTAGCTCTTGATCCGGCAAACAAACCACCGCTGGTAGCGGTGGTTTTTTTGTTTGCAAGCAGCAGATTACGCGCAGAAAAAAAGGATCTCAAGAAGATCCTTTGATCTTTTCTACGGGGTCTGACGCTCAGTGGAACGAAAACTCACGTTAAGGGATTTTGGTCATGAGATTATCAAAAAGGATCTTCACCTAGATCCTTTTAAATTAAAAATGAAGTTTTAAATCAATCTAAAGTATATATGAGTAAACTTGGTCTGACAGTTACCAATGCTTAATCAGTGAGGCACCTATCTCAGCGATCTGTCTATTTCGTTCATCCATAGTTGCCTGACTCCCCGTCGTGTAGATAACTACGATACGGGAGGGCTTACCATCTGGCCCCAGTGCTGCAATGATACCGCGAGACCCACGCTCACCGGCTCCAGATTTATCAGCAATAAACCAGCCAGCCGGAAGGGCCGAGCGCAGAAGTGGTCCTGCAACTTTATCCGCCTCCATCCAGTCTATTAATTGTTGCCGGGAAGCTAGAGTAAGTAGTTCGCCAGTTAATAGTTTGCGCAACGTTGTTGCCATTGCTACAGGCATCGTGGTGTCACGCTCGTCGTTTGGTATGGCTTCATTCAGCTCCGGTTCCCAACGATCAAGGCGAGTTACATGATCCCCCATGTTGTGCAAAAAAGCGGTTAGCTCCTTCGGTCCTCCGATCGTTGTCAGAAGTAAGTTGGCCGCAGTGTTATCACTCATGGTTATGGCAGCACTGCATAATTCTCTTACTGTCATGCCATCCGTAAGATGCTTTTCTGTGACTGGTGAGTACTCAACCAAGTCATTCTGAGAATAGTGTATGCGGCGACCGAGTTGCTCTTGCCCGGCGTCAATACGGGATAATACCGCGCCACATAGCAGAACTTTAAAAGTGCTCATCATTGGAAAACGTTCTTCGGGGCGAAAACTCTCAAGGATCTTACCGCTGTTGAGATCCAGTTCGATGTAACCCACTCGTGCACCCAACTGATCTTCAGCATCTTTTACTTTCACCAGCGTTTCTGGGTGAGCAAAAACAGGAAGGCAAAATGCCGCAAAAAAGGGAATAAGGGCGACACGGAAATGTTGAATACTCAT

>Bloc 6

GACTCTCATGAAAACTACGAGCTAAATATTCAATATGTTCCTCTTGACCAACTTTATTCTGCATTTTTTTTGAACGAGGTTTAGAGCAAGCTTCAGGAAACTGAGACAGGAATTTTATTAAAAATTTAAATTTTGAAGAAAGTTCAGGGTTAATAGCATCCATTTTTTGCTTTGCAAGTTCCTCAGCATTCTTAACAAAAGACGTCTCTTTTGACATGTTTAAAGTTTAAACCTCCTGTGTGAAATTATTATCCGCTCATAATTCCACACATTATACGAGCCGGAAGCATAAAGTGTAAAGCCTGGGGTGCCTAATGAGTGAGCTAACTCACATTAATTGCGTTGCGCTCACTGCCAATTGCTTTCCAGTCGGGAAACCTGTCGTGCCAGCTGCATTAATGAATCGGCCAACGCGCGGGGAGAGGCGGTTTGCGTATTGGGCGCTCTTCCGCTTCCTCGCTCACTGACTCGCTGCGCTCGGTCGTTCGGCTGCGGCGAGCGGTATCAGCTCACTCAAAGGCGGTAATACGGTTATCCACAGAATCAGGGGATAACGCAGGAAAGAACATGTGAGCAAAAGGCCAGCAAAAGGCCAGGAACCGTAAAAAGGCCGCGTTGCTGGCGTTTTTCCATAGGCTCCGCCCCCCTGACGAGCATCACAAAAATCGACGCTCAAGTCAGAGGTGGCGAAACCCGACAGGACTATAAAGATACCAGGCGTTTCCCCCTGGAAGCTCCCTCGTGCGCTCTCCTGTTCCGACCCTGCCGCTTACCGGATACCTGTCCGCCTTTCTCCCTTCGGGAAGCGTGGCGCTTTCTCATAGCTCACGCTGTAGGTATCTCAGTTCGGTGTAGGTCGTTCGCTCCAAGCTGGGCTGTGTGCACGAACCCCCCGTTCAGCCCGACCGCTGCGCCTTATCCGGTAACTATCGTCTTGAGTCCAACCCGGTAAGACACGACTTATCGCCACTGGCAGCAGCCACTGGTAACAGGATTAGCAGAGCGAGGTATGTAGGCGGTGCTACAGAGTTCTTGAAGTGGTGGCCTAACTACGGCTACACTAGAAGAACAGTATTTGGTATCTGCGCTCTGCTGAAGCCAGTTACCTTCGGAAAAAGAGTTGGTAGCTCTTGATCCGGCAAACAAACCACCGCTGGTAGCGGTGGTTTTTTTGTTTGCAAGCAGCAGATTACGCGCAGAAAAAAAGGATCTCAAGAAGATCCTTTGATCTTTTCTACGGGGTCTGACGCTCAGTGGAACGAAAACTCACGTTAAGGGATTTTGGTCATGAGATTATCAAAAAGGATCTTCACCTAGATCCTTTTAAATTAAAAATGAAGTTTTAAATCAATCTAAAGTATATATGAGTAAACTTGGTCTGACAGTTACCAATGCTTAATCAGTGAGGCACCTATCTCAGCGATCTGTCTATTTCGTTCATCCATAGTTGCCTGACTCCCCGTCGTGTAGATAACTACGATACGGGAGGGCTTACCATCTGGCCCCAGTGCTGCAATGATACCGCGAGACCCACGCTCACCGGCTCCAGATTTATCAGCAATAAACCAGCCAGCCGGAAGGGCCGAGCGCAGAAGTGGTCCTGCAACTTTATCCGCCTCCATCCAGTCTATTAATTGTTGCCGGGAAGCTAGAGTAAGTAGTTCGCCAGTTAATAGTTTGCGCAACGTTGTTGCCATTGCTACAGGCATCGTGGTGTCACGCTCGTCGTTTGGTATGGCTTCATTCAGCTCCGGTTCCCAACGATCAAGGCGAGTTACATGATCCCCCATGTTGTGCAAAAAAGCGGTTAGCTCCTTCGGTCCTCCGATCGTTGTCAGAAGTAAGTTGGCCGCAGTGTTATCACTCATGGTTATGGCAGCACTGCATAATTCTCTTACTGTCATGCCATCCGTAAGATGCTTTTCTGTGACTGGTGAGTACTCAACCAAGTCATTCTGAGAATAGTGTATGCGGCGACCGAGTTGCTCTTGCCCGGCGTCAATACGGGATAATACCGCGCCACATAGCAGAACTTTAAAAGTGCTCATCATTGGAAAACGTTCTTCGGGGCGAAAACTCTCAAGGATCTTACCGCTGTTGAGATCCAGTTCGATGTAACCCACTCGTGCACCCAACTGATCTTCAGCATCTTTTACTTTCACCAGCGTTTCTGGGTGAGCAAAAACAGGAAGGCAAAATGCCGCAAAAAAGGGAATAAGGGCGACACGGAAATGTTGAATACTCATACTCTTCCTTTTTCAATATTATTGAAGCATTTATCAGGGTTATTGTCTCATGAGCGGATACATATTTGAATGTATTTAGAAAAATAAACAAATAGGGGTTCCGCGCACATTTCCCCGAAAAGTGCCACCTGACGTCTAAGAAACCATTATTATCATGACATTAACCTATAAAAATAGGCGTATCACGAGGCCGCCCCTGCAGCCGAATTATATTATTTTTGCCAAATAATTTTTAACAAAAGCTCTGAAGTCTTCTTCATTTAAATTCTTAGATGATACTTCATCTGGAAAATTGTCCCAATTAGTAGCATCACGCTGTGAGTAAGTTCTAAACCATTTTTTTATTGTTGTATTATCTCTAATCTTACTACTCGATGAGTTTTCGGTATTATCTCTATTTTTAACTTGGAGCAGGTTCCATTCATTGTTTTTTTCATCATAGTGAATAAAATCAACTGCTTTAACACTTGTGCCTGAACACCATATCCATCCGGCGTAATACGACTCACTATAGGGAGAGCGGCCGCCAGATCTTCCGGATGGCTCGAGTTTTTCAGCAAGATATCGATTCAACAGGCGGGAAAAGTGACGTCACAACAAGATGAGTAAAGATATTCCCACGCCTTACATGTGGAGCTTTCAACCGCAAATGGGACTGGCGGCCGGCGCGGCTCAAGACTATTCTAGCAAAATGAATTGGTTAAGCGCCGGACCCCACATGATTTCCAGGGTGAATGGGGTACGAGCCCGGCGTAACCAAATACTGCTAGAACAAGCCGCTCTCACCGCTACACCACGTAATCAACTTAACCCTCCCTCTTGGCCAGCTGCCCTGATATATCAGGAAAATCCCCCTCCTACCACTGTACTTTTGCCTCGCGACGCCCAGGCCGAAGTCCATATGACTAACGCTGGGGCACAGCTTGCGGGCGGTGCACGTCACAGTTTCAGGTATAAAGGTCGCACTGAGCCCTATCCGTCTCCAGCTATAAAAAGAGTACTCATCAGAGGGAAAGGTATTCAGCTGAACGACGAAGTCACATCGCCATTGGGAGTCAGACCCGACGGAGTGTTTCAGCTCGGAGGGTCCGGACGTTCCTCCTTTACCGCTCGTCAAGCCTACCTGACACTACAGAGCTCATCCTCAGCTCCGAGATCTGGTGGTATTGGAACTCTCCAATTTGTGGAGGAATTTACTCCATCTGTTTACTTCAATCCTTTTTCGGGCTCGCCTGGACACTATCCTGACGCCTTCATACCCAACTTTGACGCAGTGAGTGAATCTGTGGATGGCTATGATTAATGTCTAATGGAGCGGCTGACAGAGCGCGGCTGCGACATTTAGACCACTGTCGCCAACCTCACTGCTTTGCTCGAGACATCTGTGTCTTTACCTACTTTGAGCTTCCAGAGGAGCACCCCCAGGGGCCAGCTCACGGTGTCAGAATAACAGTTGAAAAAGGAATTGATACACACCTCATTAAATTTTTCACCAAACGCCCGCTATTGGTGGAAAAAGATCAAGGAAATACTATATTAACTTTATATTGCATTTGTCCTGTTCCCGGATTACATGAAGATTTCTGCTGTCATTTGTGTGCTGAATTTAATCATCTGTAGTGGCGCTGTACCGCCTGAAGAAGAACCTAACTGTCATCCGCATTTAAGCAACATTAAAATCAACCTTTCGATCCCTCATATCACTCTTCGCTGCAGTTTTTTTTCCACACATCTCACCTGGACCTTTAACGGAAAACACGTTACCAATACAGATATAAAGTTTAAACTACACAAAGAAAACATCACTCTATTTCAACCTATTAACCTGGGATACTACCGCTGCTCAGCTCCACCCTGTACGCAAGCATTTTTTGTTGCTCCAGTTATTGACAAACGCCCTGCTCCGACAACAGCTGCTGTCACTGAGCACATCACCGAGGCAGTTTCTCCTTCTAAAGGTACAGAGGAAATTGTGTACTTTTCAAACTTTACAAACCACTTAGTTTTAAATTGTTCCTGTTCTAACTCCTTAATTTCATGGTTTGCTAACAGCTCTCTGTGCAAAACTTTCTACCAAGGAAAACTTTTGTATTCTGCTAAACTCACATTGTGTAACCAGAGCACCCCTTCCCACCTTACTCTATTGCCACCTTTTGTTGCCGGTCGTTACTTTTGCATAGGAGCTGCACGTACTAGCCCCTGTCAACAGCATTGGAATTTAACTTACTGTCCCCCACCAGTGTCGCCCTTTGTGATCAATACTGAATATTTAGACTATAATCCCTTGCTTGCTTACGGCGGTCTCGCAGCTCTTATTTTATTCCTGATTTCTAACTTGTTTCTAGTGCAACATTTGTATTCATACTAACAATGCTTTCCATTTTTCTTTTATTTCTCTTTTCTTTACCTTCTGGCTTGTATGCTCAAACAGCCGAAAGACCACTAAAAGTCGTGGTGGAAGCTGGCCATAATGTAACCCTTCCCCACCTTTCTGGTTCACACCAAACTGGCCATGTTACTTGGCTAGTAGAGACATCAGATTATGGTTCAGCTTCTCCAGACAACTTCATTTTCAGTGGACAAAAACTATGCCAGTTTACTGACAGAACCATGGTGTGGCCTTATTACAATTTACATTTTAACTGTGAAAATTATGACCTTAATCTGTTTTGGCTTAAGGTGGAAAATTCGGCTATTTACAACGTTAAAAATACAGTCAATGCTTCTGAAACAAATATTTACTATGATTTAAGAGTAGTACAAATTTTTCCACCTAAATGCATCATTACTTCAAAGTACCTTACAAACGATTATTGTCACATTACAATTAACTGCACTAACTCTGATTACCCCAATAAGGTTGTGTTTAATAATGTCAGTCGATGGTACTACGGATACGGTAAGGGCAGCCCAACCCTTCCCAACTACTTTATAACTAACTTTAATGTTTCAGGTATTACTAAAAGCTTTAATCACACTTACCCTTTTAATGAGCTCTGTGATTATCCCACATCCCAATCTCAACACAGTTTAACACATACAGTAAGCACAGTAATCTTTTTAGGAATAATTGGCTTCAGCATTTTGATTATTATAGCAGCCTTTATTTATCTGTGCTGGCATAGAAAATCTTTGTGTGTTTCTAAAACAGAACCTCTTATGCCGATTCCTTACTAGTTTTCTTTTTTCTTACAGTATGGTCACGGTTCTTCTCATCTTTTTATGCCTGCCAGTCATTTTTTCTTCTTCGACTTTTGCCGCAGTCAGTGACCTTGATCCCGAGTGTTTAGCCCCCTTTGCGGTGTACCTGATTTTCACATTTGTGACTGCTACCTGCGTCTGCAGTATTATTACTCTGCTAATCACCTCGCTCCAATTTTTTGATTACTACTACGTGAGAATTGTTTACCGCAGACACCACCCCCGTTACCAAAACCCTCAAATTGCGGCTCTTTTGCAGCTCCAACCATGAAAACAGCATTAGTTCTTTTCTTTATGTTAATCCCAGTTTGGGCTAGTTCTTGTCAACTACATAAACCATGGAATTTTTTAGATTGTTATACTAAAGAAACAAACTACATAGGCTGGGTTTATGGAATTATGTCTGGCTTAGTATTTGTCTCCTCTGTAGTTTCTTTACAACTGTATGCGCGCCTTAATTTTAGTTGGAATAAGTATACTGATGATCTTCCCGAATATCCAAACCCCCAGGATGATTTACCCCTAAATATTGTATTTCCAGAGCCCCCGCGTCCTCCTTCTGTTGTTAGCTATTTTAAGTTCACCGGTGAAGATGATTGAACCTGATCTAGAAATTGATGGAAGAATCACCGAACAGAGGCTCCTCACTGATCGCGCTAGGCGACGCCAACAGGATCAAAAAAATAAAGAGTTAATTGATTTACAAACCGTGCATCAGTGTAAAAAAGGACTTTTTTGCCTGGTAAAACAAGCTACCCTTCGCTATGAATCTTTACCAGGCAAAGAACATCAACTGTGCTACACGCTGCCCACTCAGCGACAAACCTTTACTGCAATGGTGGGCTCGGTACCTATTAAAGTGTCCCAACAAGCAGGAGAACAAGAAGGCTCTATTCGGTGCCTATGTGATAACCCTGAATGTTTGTACACTTTAATAAAAACACTGTGCGGTTTAAGAAATCTTTTACCAATGAATTAAATAAATTACTTACCGGAAATCTGAAAATACATATCGATATCTTTCTAGAAGATCTCCTACAATATTCTCAGCTGCCATGGAAAATCGATGTTCTTCTTTTATTCTCTCAAGATTTTCAGGCTGTATATTAAAACTTATATTAAGAACTATGCTAACCACCTCATCAGGAACCGTTGTAGGTGGCGTGGGTTTTCTTGGCAATC

>Bloc 7

TAATACGACTCACTATAGGGAGAGCGGCCGCCAGATCTTCCGGATGGCTCGAGTTTTTCAGCAAGATATCGATACCTATCTAATAATATACCTTATACTGGACTAGTGCCAATATTAAAATGAAGTGGGCGTAGTGTGTAATTTGATTGGGTGGAGGTGTGGCTTTGGCGTGCTTGTAAGTTTGGGCGGATGAGGAAGTGGGGCGCGGCGTGGGAGCCGGGCGCGCCGGATGTGACGTGTCGTGAGAACACCGTATTATGCCGAGTCATATGCGGAAGTGATTTTTTTCGCGGGCTTTTGGGGTTTTTTTTGTGTTGTGTAGGCGAACTTTGGCCATTTACGTCACTATTTTTCAGTATTAAAGGTGCGCACTTGCGGCAGATTGTGTCTGCTGTGCCTGGCTTTGTACTGTTTTTCTGGTAAGGCATTATGGCTGCTTTTGAGACTCTTTATGTGTATTTTACGGGACCTGGGGCTATGTTGCCTAAACAAGAGGGCGACTCTAATGCTTATGTGTTATTTTCTCCTGCGAATTTTGTTATACCTCCACATGGAGTTGTGCTTTTATATTTGCACATAGCAGTTGATATTCCTCCTGGATATTTGGGAACATTGTTTTCATTATGCGACATGAACGCCAGAGGGGTTTTTGTTGGCGCTGAAACGCTTTATCCAGGCTCAAGAATGGAGCTCAGTGTTTTGTTGTTTAATCATTCCGACGTGTTTTGCGATGTTCGCGCAAAGCAGCCAGTCGCGCGCTTGCTTTTAAGTAGAGTTGTTTTTCCACCCGTTTGCCAGGCATCTTTAATTTAACATATTTTTATTTTTCAGGCTAACCTAAAGCATGTTTCAGAGGTCGCTTGTTCATTACTCCGTGTTGTTTCCGGAGTCTTTACGGAACTATTTGCATGGCTTAGATTTTGAGGTGGTGACGTTTCTTAAAGACGTGCTACCTGAGTTTTGGCTGCTGGTAATGCATTATTTAACTCCTCCTATGCGCGATGTCTACGTTGGCGCCACGCTTACTAATATGGGTCCATTTGTGCAAGTTGTATGTTCTGTGGGAACTCCGGAACTTGTACCTGGAGGTGAACTTTCTTTGCTGCTGGCTTCTGATTTGTATGATTTTATACAACTGGCATTAAGATGTCAGCTGCGAGACCAAGGTGTGGAGCCTAATGTAAATCTACTGAATTTACTGCAGGTGTTTGAAGATCCAGACTTTTTTCAGCAAATATGAAGTACTGCCTGCGGATGGCGGTGGAGGGCGCCCTTACAGAGCTTTTTAATATTCACGGTTTAAACCTGCAAAATCAGTGTGTTCAAATAATACAACAGTGGAAGAATGAAAATTACCTGGGAATGGTTCAGTCAGGCAGTTTGATGATAGAAGAGTTTCATGATAATGCATTTGCTTTGCTTTTGTTTATCGAAATCAGAGCTGTGGCTCTTTTAGAAGCTGTTGTTGAACATTTGGAAAATCGCTTACAATTTGATCTGGCTGTGATCTTTCACCAGCACAGCGGAGGCGATCGCTGCCACCTGCGAGATTTACGCATTCAAATCCTTGCTGACCGTCTTGATTAAGTTTTTATGCCTCTACCTTGTATTCCTCCTCCTCCTGTAAGTCGGGACACGGCTGCCTGCATAGCATGGCTTGGTTTAGCCCATGCATCCTGTGTGGATACTCTGCGCTTTATTAAACATCATGATTTGAAGATAACACCTGAAGCTGAATACATTTTAGCAAGCCTGCGAGAGTGGTTGTACTTTGCTTTTTTGACGGAACGCCAACGCTGCAAACAAAAAGGACGAGGTGCGATAACCAGTGGTCGTACGTGGTTTTGTTTTTTTAAGTACGAAGACGCTCGCAAGTCTGTTGTTTACGATGCAGCGCGACAGACGGTATCGCTACAGATTGGCACCATACAACAAGTACCAACTACCGCCCTGTGAGGAACAGTCAAAAGCTACGTTGAGTACTTCGGAAAATTCTTTATGGCCTGAGTGTAATAGTCTGACTTTACATAATGTAAGTGAGGTAAGAGGCATTCCTTCATGTGTAGGTTTTACAGTGCTGCAGGAATGGCCAATACCGTGGGATATGATTCTAACTGATTATGAGATGTTTATTTTGAAAAAATACATGAGTGTATGTATGTGTTGTGCCACTATAAATGTTGAAGTTACTCAATTATTACATGGTCATGAGCGGTGGCTTATTCATTGTCATTGCCAGCGTCCGGGTTCACTACAGTGTATGTCAGCTGGGATGCTTTTGGGACGCTGGTTCAAAATGGCCGTATATGGCGCCTTAATTAACAAAAGGTGTTTTTGGTATCGGGAGGTTGTTAACCATTTAATGCCTAAAGAGGTGATGTATGTGGGAAGCACCTTTGTTAGAGGTCGCCATTTAATTTACTTTAAAATTATGTATGATGGCCATGCCTGGTTAGCGTTAGAAAAAGTTAGTTTTGGATGGAGCGCCTTTAATTATGGAATTTTAAATAACATGTTAGTGCTGTGTTGTGATTATTGTAAAGACTTAAGTGAGATACGCATGCGCTGTTGCGCTCGTCGTACCAGACTGCTAATGTTAAAAGTTGTTCAAGTAATTGCTGAAAACACTGTTCGCCCTCTAAAACATAGTCGGCATGAACGTTATCGTCAGCAACTGCTAAAGGGTTTAATTATGCATCATCGAGCAATTTTATTTGGAGATTATAATCAACGAGAGAATCCTTGGGCGGCTGATGGACACTGACTGTTGTTACTTTTTGTAGGATAAAATCATGGACCTGGTTTTGGATGGGGAATGCCGCTTGAGTGACTGTGCGGGCGAGGGATTCGTTTCCATCACCGACCCTCGCTTTGCCCGTAAAGAAACTGTGTGGACGCTAACGCCAAAAAACCTAAGTCGAAATATTCAAGTGCAGTTGTTTTCAGCTACAAAGGGGGAAAGGGAGGTATACAAGGTAAAATGGGAAGGAGGCAGTTTAACCACGCGTATAGTGTAAAAAAAAAAAAAAATAAACAATAAGTACGCAATTTGTGTTTTGTGTGTGTTTTTATTCTTGGGTAATGTAAGAAAAGGAGCAGGATGGTGTAGAGAAAGGCTGATTTATATAGTTTGATAGACCTGACCACATGAATGTTAAAGAGTATCCATTTAGCGACGTAATGCCATTAAATGCAACTTTCATTGTTATAGGTTTAGATGTATCTCCCTGTAAGTACGTGAGACTTACCATTTGGCTTTTAGCCTCACTGGCATTTGGTCTAGGGTAAGCACTCACATTAGGCATAAAACCTAGACCATTGGTAACAGTATTGGTAGACACTGATTGGCCTTGTCTATATCCCCACGAAGCTTGGGGAACCAGGGCAGTAGGGGTTGATGTGATTAATCTTCCCTGTTCATCAAACACTAAATGCACTCCTACAGTGGTAGTAGTACTTTGGATATTTAGGAGATTACCCTTAACACCCACTAAACTTACAATGCCATTAACAATAGATCCGTTTTTTGTTAAGCACAGGGTGAGTTTTGCATCTAGCTCTTGTATGAGGCTGCAGTTTGGTGGTGGGTCAGGAGTTGTCCACAGTGTCAGAGGGTCGTATGGAGTGTTGCTGCTGCTGCCTAAGGCAATGTTATTGTTTGAATCAAAAATAAGTCCAGCTCCCAACTTAACCCTAAGTTGGCCATTATTAAATGTAAGCCCCTGACCTGCATTAAGAGCTATTTGATTGCCACTAAACATTAAACCTTTTTCGGTACTAATATCAGTTGTTAAGTTCCAGTTTGTAGATTGGTTATAAATTAGTCCCAATCCCCGTCTTAAGGACAGGTTATTGCTCGCATCAAAGGGATAATTAACATCTAAAATTATTCTACCGCCAGAAGTTCTCATTCCCCCGGCAACGTTTACTTTCATGACCCCATTATCGAATGTTAAACCATCCCCTATTCTTGTTACTAATTGAGACCCCGACACTTGAAGACCGTTACCAGTGGAAACAGTCAATGTGTCTTGTGAAATAGTCAGAGGATTGGCAACAGACAGACTTAAAGTGGATCCTGTGCTGTTGAGGGGAGCAGTTGTAGACAAGGCTAAGGAACCGTTTATTACTGTAATGGGACTAGTGACACTTATTCCCAACCCATCGGAGCTCACCATTAACGGATCTGCCGTAGGTAGGGTAAGAGCGTTGTTGCGAACACTTATGGGGTCTGCAGTAGCAACAGATAGGGCACCGCTAGAATTTACAACTAAGGGAGTTTCAGTGGTGAGATGCAAATTGTTGTTACTAACGTCCAGGGGAGCAGAAAGATTTAAACCTAGGTTTCCACCTCCATCTAAGCTTAGAGGGGCTATGGTGGCCAAGCCCAAACGCGAAGACTCTACTGTAATGGGAGGGGCGGTTATTAAGGCTAAGCTTTCATCCGTTGTGGTTAAGGGCGCTCTTGTGTTAAGTGTGAGGGCACTAGCCTTTACTGCTAGGGGGGCGCTCCAAGAAAGTTTAAGACCTTGTGAGGTGTTGGTAAGGGGCTCCAAAACATTGATATTATTACTAGCTGTAAGTTGACCTTGGGCATTAAGTTTTATTCCGTCCCCTAGCTTGAGTGTAAGGGTTCCATTTTCAGTTACAATGGGGTCTTTGTAATTAAGTGCTAATACACCTGGTGGTTTTTCTTGAAGACCATTGGAAGAAGTAAAAGGGGGTGTAACAAAGGGTACGTCTGATGTGTCAAATGGGTCAAAAGGGTAAACGGGGTTGAAGTCATCATTTTCTTCTGTTTCTTCAGCATACTGGGTTCTGCTGCGCTTCATCTAGAAAAGATGAAGATGGTGGGTAGGGATTGGGAAGAAGAATCTGACCTTCCATTTAAGATTTGGAGAAAGTTTGCTGTTTGCAAGTCTGTCAGGTTTGACAGTTGGGAAGAGGGAAATTTTATAAGAGTACACGGAGACCATGATGTATTTTCAGATTTCCGGTAAGTATCGATATCTTTCTAGAAGATCTCCTACAATATTCTCAGCTGCCATGGAAAATCGATGTTCTTCTTTTATTCTCTCAAGATTTTCAGGCTGTATATTAAAACTTATATTAAGAACTATGCTAACCACCTCATCAGGAACCGTTGTAGGTGGCGTGGGTTTTCTTGGCAATCGACTCTCATGAAAACTACGAGCTAAATATTCAATATGTTCCTCTTGACCAACTTTATTCTGCATTTTTTTTGAACGAGGTTTAGAGCAAGCTTCAGGAAACTGAGACAGGAATTTTATTAAAAATTTAAATTTTGAAGAAAGTTCAGGGTTAATAGCATCCATTTTTTGCTTTGCAAGTTCCTCAGCATTCTTAACAAAAGACGTCTCTTTTGACATGTTTAAAGTTTAAACCTCCTGTGTGAAATTATTATCCGCTCATAATTCCACACATTATACGAGCCGGAAGCATAAAGTGTAAAGCCTGGGGTGCCTAATGAGTGAGCTAACTCACATTAATTGCGTTGCGCTCACTGCCAATTGCTTTCCAGTCGGGAAACCTGTCGTGCCAGCTGCATTAATGAATCGGCCAACGCGCGGGGAGAGGCGGTTTGCGTATTGGGCGCTCTTCCGCTTCCTCGCTCACTGACTCGCTGCGCTCGGTCGTTCGGCTGCGGCGAGCGGTATCAGCTCACTCAAAGGCGGTAATACGGTTATCCACAGAATCAGGGGATAACGCAGGAAAGAACATGTGAGCAAAAGGCCAGCAAAAGGCCAGGAACCGTAAAAAGGCCGCGTTGCTGGCGTTTTTCCATAGGCTCCGCCCCCCTGACGAGCATCACAAAAATCGACGCTCAAGTCAGAGGTGGCGAAACCCGACAGGACTATAAAGATACCAGGCGTTTCCCCCTGGAAGCTCCCTCGTGCGCTCTCCTGTTCCGACCCTGCCGCTTACCGGATACCTGTCCGCCTTTCTCCCTTCGGGAAGCGTGGCGCTTTCTCATAGCTCACGCTGTAGGTATCTCAGTTCGGTGTAGGTCGTTCGCTCCAAGCTGGGCTGTGTGCACGAACCCCCCGTTCAGCCCGACCGCTGCGCCTTATACGGTAACTATCGTCTTGAGTCCAACCCGGTAAGACACGACTTATCGCCACTGGCAGCAGCCACTGGTAACAGGATTAGCAGAGCGAGGTATGTAGGCGGTGCTACAGAGTTCTTGAAGTGGTGGCCTAACTACGGCTACACTAGAAGAACAGTATTTGGTATCTGCGCTCTGCTGAAGCCAGTTACCTTCGGAAAAAGAGTTGGTAGCTCTTGATCCGGCAAACAAACCACCGCTGGTAGCGGTGGTTTTTTTGTTTGCAAGCAGCAGATTACGCGCAGAAAAAAAGGATCTCAAGAAGATCCTTTGATCTTTTCTACGGGGTCTGACGCTCAGTGGAACGAAAACTCACGTTAAGGGATTTTGGTCATGAGATTATCAAAAAGGATCTTCACCTAGATCCTTTTAAATTAAAAATGAAGTTTTAAATCAATCTAAAGTATATATGAGTAAACTTGGTCTGACAGTTACCAATGCTTAATCAGTGAGGCACCTATCTCAGCGATCTGTCTATTTCGTTCATCCATAGTTGCCTGACTCCCCGTCGTGTAGATAACTACGATACGGGAGGGCTTACCATCTGGCCCCAGTGCTGCAATGATACCGCGAGACCCACGCTCACCGGCTCCAGATTTATCAGCAATAAACCAGCCAGCCGGAAGGGCCGAGCGCAGAAGTGGTCCTGCAACTTTATCCGCCTCCATCCAGTCTATTAATTGTTGCCGGGAAGCTAGAGTAAGTAGTTCGCCAGTTAATAGTTTGCGCAACGTTGTTGCCATTGCTACAGGCATCGTGGTGTCACGCTCGTCGTTTGGTATGGCTTCATTCAGCTCAGGTTCCCAACGATCAAGGCGAGTTACATGATCCCCCATGTTGTGCAAAAAAGCGGTTAGCTCCTTCGGTCCTCCGATCGTTGTCAGAAGTAAGTTGGCCGCAGTGTTATCACTCATGGTTATGGCAGCACTGCATAATTCTCTTACTGTCATGCCATCCGTAAGATGCTTTTCTGTGACTGGTGAGTACTCAACCAAGTCATTCTGAGAATAGTGTATGCGGCGACCGAGTTGCTCTTGCCCGGCGTCAATACGGGATAATACCGCGCCACATAGCAGAACTTTAAAAGTGCTCATCATTGGAAAACGTTCTTCGGGGCGAAAACTCTCAAGGATCTTACCGCTGTTGAGATCCAGTTCGATGTAACCCACTCGTGCACCCAACTGATCTTCAGCATCTTTTACTTTCACCAGCGTTTCTGGGTGAGCAAAAACAGGAAGGCAAAATGCCGCAAAAAAGGGAATAAGGGCGACACGGAAATGTTGAATACTCATACTCTTCCTTTTTCAATATTATTGAAGCATTTATCAGGGTTATTGTCTCATGAGCGGATACATATTTGAATGTATTTAGAAAAATAAACAAATAGGGGTTCCGCGCACATTTCCCCGAAAAGTGCCACCTGACGTCTAAGAAACCATTATTATCATGACATTAACCTATAAAAATAGGCGTATCACGAGGCCGCCCCTGCAGCCGAATTATATTATTTTTGCCAAATAATTTTTAACAAAAGCTCTGAAGTCTTCTTCATTTAAATTCTTAGATGATACTTCATCTGGAAAATTGTCCCAATTAGTAGCATCACGCTGTGAGTAAGTTCTAAACCATTTTTTTATTGTTGTATTATCTCTAATCTTACTACTCGATGAGTTTTCGGTATTATCTCTATTTTTAACTTGGAGCAGGTTCCATTCATTGTTTTTTTCATCATAGTGAATAAAATCAACTGCTTTAACACTTGTGCCTGAACACCATATCCATCCGGCG

>Bloc 5 3x_FLAG-DBP

GCCTGGTTTCCATTTTTTGAAAAGACTGAGAACCGTCTGCATGATGCATAATGCGGACGGGCGGCATGCTGAAACCCATTACTCCTAAAACTGCTCTTGGTGGTTCTGCCTCTTCTTCTTCTGCACTCTCTGGGGAAAGAGGTATCGCAGCCATAGATTTCTTGACTTTTTTCTTTGGAGGTAAAGGCACAGCTTCCAGTTCTTCTTCGCTTTCGGAATCCAGAAAGTATCTGCCCATTTTTGGCGGCGGCGGCTGAGCGCTGCGGTCTGGGGTGCGCTCCCTCTGTGAGTGCTGATTGCTGGCTGCGGCCGCCTTGTCATCGTCATCCTTGTAATCGATATCATGATCTTTATAATCACCGTCATGGTCTTTGTAGTCCATTATTTAATCCTAGGCAAAGAAACACATGATGGATCTGGAGCCACAGGAAAGCTTAACCGCCCCCACCGCTCCCGCCATTGGCGCTACGGCTGTCATGGAGAAGGACAAAAGTCTACTCATACCCCAAGACGCACCGGTTGAGCAGAACTTGGGCTACGAGACTCCCCCCGAGGAATTTGAAGGCTTTCTTCAAATCCAAAAGCAACCAAATGAGCAAAACGCTGGGCTCGAGGACCATGACTACCTAAACGAGGGAGATGTCCTGTTTAAACATCTACAGCGACAAAGCACTATCGTTCGCGACGCCATATCTGATCGCTCTTCAATACCAGTTTCAATTGCAGAACTATCTTGCATCTACGAACGCAACCTGTTCTCCCCACGTGTGCCCCCTAAACGGCAAGCCAACGGCACATGCGAGCCAAATCCTCGCCTTAACTTCTACCCAGTTTTTGCAGTGCCAGAAGCACTGGCAACATACCATATTTTCTTTAAAAATCACAAAATACCCCTATCCTGTCGAGCTAACCGCAGCCGCGCAGATGAGCTTCTTGCTTTAAGGGCTGGCGCTTCCATACCTGGGATTGTGTCCTTGGAAGAGGTGCCTAAAATTTTTGAAGGTTTAGGTCGGGATGAAAAACGAGCAGCAAATGCCCTGCAAAAAGAAAATGAACAAAATCACCATGGGAATAGTGCTCTAATAGAACTGGAAGGTGACAATGCCCGCCTGGCAGTTTTAAAGCGCAATATTGAGGTTACTCACTTTGCCTACCCGGCAGTAAATCTTCCGCCAAAGGTAATGAGCGCAGTGATGAATCAGCTACTAATTAAGCGAGCCCAACCCATTGACAAAGATGCAAACTTGCAAGACCCGGAGGCAACAGATGATGGAAAGCCGGTTGTAAGCGACGAGCAATTAACTAAGTGGTTGGGAACAGACAATTCCAACGAACTACAACAGCGGCGTAAACTCATGATGGCCGCCGTACTTGTAACTGTGGAACTCGAGTGCATGCATCGTTTTTTCTCCGACATCACCACATTGCGCAAAATTGAGGAATGTCTTCACTACACTTTCCGCCATGGCTACGTGCGCCAAGCCTGTAAAATTTCTAATGTGGAGCTGAGCAATCTAGTTTCTTACATGGGCATCTTGCATGAAAACCGATTGGGACAGAACGTGCTACACTCAACACTACGCGATGAAGCACGCAGAGATTACGTGCGAGACTGCATTTACCTTTTCCTGTTACATACCTGGCAAACTGGGATGGGTGTTTGGCAGCAATGCTTGGAAGAAAAAAACCTTCGAGAACTAAACAAACTGTTAGACAGAGCACTAAAATCCCTATGGACCGGTTTTGACGAACGGACAGTAGCTGCAGAGCTAGCTGACATAATTTTCCCAGAAAGGTTAATGATAACCTTGCAAAACGGCTTGCCTGACTTTATGAGTCAAAGTATGCTGCACAATTATCGCTCTTTTATATTAGAGCGTTCTGGGATGCTTCCTAGCATGTGTTGTGCACTTCCTTCAGATTTTGTGCCTATATATTTTAGAGAGTGCCCCCCTCCCCTGTGGAGCCACTGCTACTTACTACGACTTGCTAACTACCTAGCTTACCACTCAGACCTTATGACAGATTCAAGCGGCGAAGGCCTAATGGAGTGTCACTGCCGCTGCAATCTTTGCACCCCCCACCGTTCTTTGGTTTGCAATACTGAACTATTAAGTGAAAGTCAAGTCATTGGTACCTTCGAAATGCAGGGACCGCAGTCTGACAGCAATTTCACGACGAACCTAAGACTTACCCCTGGGCTTTGGACTTCTGCCTACCTGCGCAAATTTGAACCCCAAGATTACCACGCCCACAGTATCAATTTTTACGAAGACCAATCCAAACCCCCAAAAGCGCCACTAACGGCTTGCGTCATTACGCAGGGAAAAATTCTAGCCCAATTGCATGCTATTAAGCAAGCGCGCGAAGAGTTTTTACTTAAAAAAGGACACGGAGTGTACCTTGATCCCCAAACCGGCGAGGAACTAAACCTTCCATCACCTTTGTGTGCTACTGCGTCTCCCCATTCGCAGCATGTCCCCGAAAGCCGCAAAACAGGCTATTGCGCAGCAACGCTCAAAGAAACAGCAGCAACGGCAGGAAATCTGGGAGGAAGAATCTTGGGAGAGTCAGGCAGAGGACGAGGTCGAGGACTTGGAAGAATGGGAGGAGGAGGAGGCGGACAGCCTAGACGAGGATCCAGAGGAGGAGGAGGAAGGTTCCAAGGACGGAGCGACCGCCGCAAAACCGTCGCTTTCAACCAAGCCCTCTCCAATGAAACCCGCTGTGAGCAAATCTCAGAAAGCCAGCCGTAGATGGGACACCATTGAAACCAGCGCCGCAAACTTGGGTAAGAATCGCAAGCAGGCGCGTCGGGGCTACTGCTCATGGCGGGCTCACAAAAGTAATATTGTAGCCTGCTTGCAGCACTGCGGGGGGAATATCTCATTTGCAAGGCGGTATTTGCTATACCATGATGGAGTGGCGATTCCAAGGAATGTCCTCCATTACTACCGTCATCTCTACAGCCCCTTTGAAGAGCTCGACAAGGAACCGACCTGCAACAGCCAAGCGGCCCACTAGAATCGGCAACAGCAGCAACAAGGAAAGTCCTGAGGCGCGCGAGTTAAGAAAACGCATTTTTCCCACTTTATATGCTATTTTTCAGCAGAGTCGAGGTCAAGAACACGAACTGAAAATAAAAAACCGTTCCCTGCGTTCACTTACCCGCAGCTGTCTCTACCTCAAAAGCGAAGATCAGTTGCAACGCACCTTGCAGGACGCAGAAGCTCTGTTCAATAAATACTGCTCCCTCTCGCTTAAAGAGTAAAAAAAGCCCGCGCGCGGACTTTCAACAGGCGGGAAAAGTGAATCGATATCTTTCTAGAAGATCTCCTACAATATTCTCAGCTGCCATGGAAAATCGATGTTCTTCTTTTATTCTCTCAAGATTTTCAGGCTGTATATTAAAACTTATATTAAGAACTATGCTAACCACCTCATCAGGAACCGTTGTAGGTGGCGTGGGTTTTCTTGGCAATCGACTCTCATGAAAACTACGAGCTAAATATTCAATATGTTCCTCTTGACCAACTTTATTCTGCATTTTTTTTGAACGAGGTTTAGAGCAAGCTTCAGGAAACTGAGACAGGAATTTTATTAAAAATTTAAATTTTGAAGAAAGTTCAGGGTTAATAGCATCCATTTTTTGCTTTGCAAGTTCCTCAGCATTCTTAACAAAAGACGTCTCTTTTGACATGTTTAAAGTTTAAACCTCCTGTGTGAAATTATTATCCGCTCATAATTCCACACATTATACGAGCCGGAAGCATAAAGTGTAAAGCCTGGGGTGCCTAATGAGTGAGCTAACTCACATTAATTGCGTTGCGCTCACTGCCAATTGCTTTCCAGTCGGGAAACCTGTCGTGCCAGCTGCATTAATGAATCGGCCAACGCGCGGGGAGAGGCGGTTTGCGTATTGGGCGCTCTTCCGCTTCCTCGCTCACTGACTCGCTGCGCTCGGTCGTTCGGCTGCGGCGAGCGGTATCAGCTCACTCAAAGGCGGTAATACGGTTATCCACAGAATCAGGGGATAACGCAGGAAAGAACATGTGAGCAAAAGGCCAGCAAAAGGCCAGGAACCGTAAAAAGGCCGCGTTGCTGGCGTTTTTCCATAGGCTCCGCCCCCCTGACGAGCATCACAAAAATCGACGCTCAAGTCAGAGGTGGCGAAACCCGACAGGACTATAAAGATACCAGGCGTTTCCCCCTGGAAGCTCCCTCGTGCGCTCTCCTGTTCCGACCCTGCCGCTTACCGGATACCTGTCCGCCTTTCTCCCTTCGGGAAGCGTGGCGCTTTCTCATAGCTCACGCTGTAGGTATCTCAGTTCGGTGTAGGTCGTTCGCTCCAAGCTGGGCTGTGTGCACGAACCCCCCGTTCAGCCCGACCGCTGCGCCTTATCCGGTAACTATCGTCTTGAGTCCAACCCGGTAAGACACGACTTATCGCCACTGGCAGCAGCCACTGGTAACAGGATTAGCAGAGCGAGGTATGTAGGCGGTGCTACAGAGTTCTTGAAGTGGTGGCCTAACTACGGCTACACTAGAAGAACAGTATTTGGTATCTGCGCTCTGCTGAAGCCAGTTACCTTCGGAAAAAGAGTTGGTAGCTCTTGATCCGGCAAACAAACCACCGCTGGTAGCGGTGGTTTTTTTGTTTGCAAGCAGCAGATTACGCGCAGAAAAAAAGGATCTCAAGAAGATCCTTTGATCTTTTCTACGGGGTCTGACGCTCAGTGGAACGAAAACTCACGTTAAGGGATTTTGGTCATGAGATTATCAAAAAGGATCTTCACCTAGATCCTTTTAAATTAAAAATGAAGTTTTAAATCAATCTAAAGTATATATGAGTAAACTTGGTCTGACAGTTACCAATGCTTAATCAGTGAGGCACCTATCTCAGCGATCTGTCTATTTCGTTCATCCATAGTTGCCTGACTCCCCGTCGTGTAGATAACTACGATACGGGAGGGCTTACCATCTGGCCCCAGTGCTGCAATGATACCGCGAGACCCACGCTCACCGGCTCCAGATTTATCAGCAATAAACCAGCCAGCCGGAAGGGCCGAGCGCAGAAGTGGTCCTGCAACTTTATCCGCCTCCATCCAGTCTATTAATTGTTGCCGGGAAGCTAGAGTAAGTAGTTCGCCAGTTAATAGTTTGCGCAACGTTGTTGCCATTGCTACAGGCATCGTGGTGTCACGCTCGTCGTTTGGTATGGCTTCATTCAGCTCCGGTTCCCAACGATCAAGGCGAGTTACATGATCCCCCATGTTGTGCAAAAAAGCGGTTAGCTCCTTCGGTCCTCCGATCGTTGTCAGAAGTAAGTTGGCCGCAGTGTTATCACTCATGGTTATGGCAGCACTGCATAATTCTCTTACTGTCATGCCATCCGTAAGATGCTTTTCTGTGACTGGTGAGTACTCAACCAAGTCATTCTGAGAATAGTGTATGCGGCGACCGAGTTGCTCTTGCCCGGCGTCAATACGGGATAATACCGCGCCACATAGCAGAACTTTAAAAGTGCTCATCATTGGAAAACGTTCTTCGGGGCGAAAACTCTCAAGGATCTTACCGCTGTTGAGATCCAGTTCGATGTAACCCACTCGTGCACCCAACTGATCTTCAGCATCTTTTACTTTCACCAGCGTTTCTGGGTGAGCAAAAACAGGAAGGCAAAATGCCGCAAAAAAGGGAATAAGGGCGACACGGAAATGTTGAATACTCATACTCTTCCTTTTTCAATATTATTGAAGCATTTATCAGGGTTATTGTCTCATGAGCGGATACATATTTGAATGTATTTAGAAAAATAAACAAATAGGGGTTCCGCGCACATTTCCCCGAAAAGTGCCACCTGACGTCTAAGAAACCATTATTATCATGACATTAACCTATAAAAATAGGCGTATCACGAGGCCGCCCCTGCAGCCGAATTATATTATTTTTGCCAAATAATTTTTAACAAAAGCTCTGAAGTCTTCTTCATTTAAATTCTTAGATGATACTTCATCTGGAAAATTGTCCCAATTAGTAGCATCACGCTGTGAGTAAGTTCTAAACCATTTTTTTATTGTTGTATTATCTCTAATCTTACTACTCGATGAGTTTTCGGTATTATCTCTATTTTTAACTTGGAGCAGGTTCCATTCATTGTTTTTTTCATCATAGTGAATAAAATCAACTGCTTTAACACTTGTGCCTGAACACCATATCCATCCGGCGTAATACGACTCACTATAGGGAGAGCGGCCGCCAGATCTTCCGGATGGCTCGAGTTTTTCAGCAAGATATCGATTAGCTCATTGGAGTACAAGCTTGACTGTTTTATTAAAAATCAAATGGCTCTTCGCGACAGTCGCCGTGGTTGGTGGGCAGGGATATGTTTCTGTACTGCAAACGCTGATGCCACTTGAATTCTGGAATAACAAGCCTAGGGGGGGAGCCGTCAAAATTTTCTCCCCACAGCTGGCGCACAAGTTGCAGGGCGCCCATAACATCAGGAGCAGAAATCTTGAAGTCGCAATTAGGGCCAGCATTGCCGCGCGCATTGCGATAAACTGGATTTGCGCACTGAAAAACCAACAAACACGGATACTTAATACTGGCTAACGCTCCAGGGTCGGTTACTTCGTTGATATCAATGTTATCCACATTGCTGAGGTTAAAAGGAGTGATTTTACACAGTTGACGCCCCATCCGTGGCAGGCCATCTTGCTTGTTTAAACATTCGCAGCGCACTGGCATAAGGAGACGTTTTTGCCCATGTCGCATGTGAGGGTAGTCGGCCAGCATAAAAGCTTCAATTTGCCTAAAAGCTATTTGAGCCTTCATTCCTTCAGAATAAAACAAGCCGCAGGACTTTCCGGAGAAAGAATTATTCCCGCAGCCAACATCATGAAAACAGCAGCGGGCATCGTCGTTTTTAATTTGAACTACATTACGCCCCCAGCGGTTTTGCGCCACCTTGGCTTTCGAGGGGTTCTCTTTCAACGCTCGTTGCCCACTTTCGCTGGTTACATCCATTTCCACCAAATGCTCTTTGCGCACCATCTCCATTCCATGCAGGCATCTAAGCTCCCCTTCGCGCTCGGTACACTTATGCTCCCACACGCAGCAACCGGTGGGTTCCCAGGAATTCTGTTGGACACCGGCATAAGCTTGCATATATCCTTGCAAAAAGCGTCCCATGAGCTCCTGAAAGGTTTTTTGGGATGAAAAAGTCAGCTGCAAACCGCGCTTTTCTTCGTTGAGCCATGTTGTGCATATTTTCTTGTACACGCTGCCCTGATCCGGCAAAAAACGAAAGGTGGCGCGCTCGTCGTGATCCACATGGTACTTTTCCATTAGCATAGCCATGGCTTCCATGCCTTTTTCCCAAGCTGAAACTAGGGGCTGGCTTGCCGGATTGCGAACAACAACAACATTCTTTTCATTTTCGTCGCTGTTTTGAGCGGAAGCCTTCAAAACGTGTACCT

>Bloc 5 GFP-DBP

TTGCGTTGCGCTCACTGCCAATTGCTTTCCAGTCGGGAAACCTGTCGTGCCAGCTGCATTAATGAATCGGCCAACGCGCGGGGAGAGGCGGTTTGCGTATTGGGCGCTCTTCCGCTTCCTCGCTCACTGACTCGCTGCGCTCGGTCGTTCGGCTGCGGCGAGCGGTATCAGCTCACTCAAAGGCGGTAATACGGTTATCCACAGAATCAGGGGATAACGCAGGAAAGAACATGTGAGCAAAAGGCCAGCAAAAGGCCAGGAACCGTAAAAAGGCCGCGTTGCTGGCGTTTTTCCATAGGCTCCGCCCCCCTGACGAGCATCACAAAAATCGACGCTCAAGTCAGAGGTGGCGAAACCCGACAGGACTATAAAGATACCAGGCGTTTCCCCCTGGAAGCTCCCTCGTGCGCTCTCCTGTTCCGACCCTGCCGCTTACCGGATACCTGTCCGCCTTTCTCCCTTCGGGAAGCGTGGCGCTTTCTCATAGCTCACGCTGTAGGTATCTCAGTTCGGTGTAGGTCGTTCGCTCCAAGCTGGGCTGTGTGCACGAACCCCCCGTTCAGCCCGACCGCTGCGCCTTATCCGGTAACTATCGTCTTGAGTCCAACCCGGTAAGACACGACTTATCGCCACTGGCAGCAGCCACTGGTAACAGGATTAGCAGAGCGAGGTATGTAGGCGGTGCTACAGAGTTCTTGAAGTGGTGGCCTAACTACGGCTACACTAGAAGAACAGTATTTGGTATCTGCGCTCTGCTGAAGCCAGTTACCTTCGGAAAAAGAGTTGGTAGCTCTTGATCCGGCAAACAAACCACCGCTGGTAGCGGTGGTTTTTTTGTTTGCAAGCAGCAGATTACGCGCAGAAAAAAAGGATCTCAAGAAGATCCTTTGATCTTTTCTACGGGGTCTGACGCTCAGTGGAACGAAAACTCACGTTAAGGGATTTTGGTCATGAGATTATCAAAAAGGATCTTCACCTAGATCCTTTTAAATTAAAAATGAAGTTTTAAATCAATCTAAAGTATATATGAGTAAACTTGGTCTGACAGTTACCAATGCTTAATCAGTGAGGCACCTATCTCAGCGATCTGTCTATTTCGTTCATCCATAGTTGCCTGACTCCCCGTCGTGTAGATAACTACGATACGGGAGGGCTTACCATCTGGCCCCAGTGCTGCAATGATACCGCGAGACCCACGCTCACCGGCTCCAGATTTATCAGCAATAAACCAGCCAGCCGGAAGGGCCGAGCGCAGAAGTGGTCCTGCAACTTTATCCGCCTCCATCCAGTCTATTAATTGTTGCCGGGAAGCTAGAGTAAGTAGTTCGCCAGTTAATAGTTTGCGCAACGTTGTTGCCATTGCTACAGGCATCGTGGTGTCACGCTCGTCGTTTGGTATGGCTTCATTCAGCTCCGGTTCCCAACGATCAAGGCGAGTTACATGATCCCCCATGTTGTGCAAAAAAGCGGTTAGCTCCTTCGGTCCTCCGATCGTTGTCAGAAGTAAGTTGGCCGCAGTGTTATCACTCATGGTTATGGCAGCACTGCATAATTCTCTTACTGTCATGCCATCCGTAAGATGCTTTTCTGTGACTGGTGAGTACTCAACCAAGTCATTCTGAGAATAGTGTATGCGGCGACCGAGTTGCTCTTGCCCGGCGTCAATACGGGATAATACCGCGCCACATAGCAGAACTTTAAAAGTGCTCATCATTGGAAAACGTTCTTCGGGGCGAAAACTCTCAAGGATCTTACCGCTGTTGAGATCCAGTTCGATGTAACCCACTCGTGCACCCAACTGATCTTCAGCATCTTTTACTTTCACCAGCGTTTCTGGGTGAGCAAAAACAGGAAGGCAAAATGCCGCAAAAAAGGGAATAAGGGCGACACGGAAATGTTGAATACTCATACTCTTCCTTTTTCAATATTATTGAAGCATTTATCAGGGTTATTGTCTCATGAGCGGATACATATTTGAATGTATTTAGAAAAATAAACAAATAGGGGTTCCGCGCACATTTCCCCGAAAAGTGCCACCTGACGTCTAAGAAACCATTATTATCATGACATTAACCTATAAAAATAGGCGTATCACGAGGCCGCCCCTGCAGCCGAATTATATTATTTTTGCCAAATAATTTTTAACAAAAGCTCTGAAGTCTTCTTCATTTAAATTCTTAGATGATACTTCATCTGGAAAATTGTCCCAATTAGTAGCATCACGCTGTGAGTAAGTTCTAAACCATTTTTTTATTGTTGTATTATCTCTAATCTTACTACTCGATGAGTTTTCGGTATTATCTCTATTTTTAACTTGGAGCAGGTTCCATTCATTGTTTTTTTCATCATAGTGAATAAAATCAACTGCTTTAACACTTGTGCCTGAACACCATATCCATCCGGCGTAATACGACTCACTATAGGGAGAGCGGCCGCCAGATCTTCCGGATGGCTCGAGTTTTTCAGCAAGATATCGATTAGCTCATTGGAGTACAAGCTTGACTGTTTTATTAAAAATCAAATGGCTCTTCGCGACAGTCGCCGTGGTTGGTGGGCAGGGATATGTTTCTGTACTGCAAACGCTGATGCCACTTGAATTCTGGAATAACAAGCCTAGGGGGGGAGCCGTCAAAATTTTCTCCCCACAGCTGGCGCACAAGTTGCAGGGCGCCCATAACATCAGGAGCAGAAATCTTGAAGTCGCAATTAGGGCCAGCATTGCCGCGCGCATTGCGATAAACTGGATTTGCGCACTGAAAAACCAACAAACACGGATACTTAATACTGGCTAACGCTCCAGGGTCGGTTACTTCGTTGATATCAATGTTATCCACATTGCTGAGGTTAAAAGGAGTGATTTTACACAGTTGACGCCCCATCCGTGGCAGGCCATCTTGCTTGTTTAAACATTCGCAGCGCACTGGCATAAGGAGACGTTTTTGCCCATGTCGCATGTGAGGGTAGTCGGCCAGCATAAAAGCTTCAATTTGCCTAAAAGCTATTTGAGCCTTCATTCCTTCAGAATAAAACAAGCCGCAGGACTTTCCGGAGAAAGAATTATTCCCGCAGCCAACATCATGAAAACAGCAGCGGGCATCGTCGTTTTTAATTTGAACTACATTACGCCCCCAGCGGTTTTGCGCCACCTTGGCTTTCGAGGGGTTCTCTTTCAACGCTCGTTGCCCACTTTCGCTGGTTACATCCATTTCCACCAAATGCTCTTTGCGCACCATCTCCATTCCATGCAGGCATCTAAGCTCCCCTTCGCGCTCGGTACACTTATGCTCCCACACGCAGCAACCGGTGGGTTCCCAGGAATTCTGTTGGACACCGGCATAAGCTTGCATATATCCTTGCAAAAAGCGTCCCATGAGCTCCTGAAAGGTTTTTTGGGATGAAAAAGTCAGCTGCAAACCGCGCTTTTCTTCGTTGAGCCATGTTGTGCATATTTTCTTGTACACGCTGCCCTGATCCGGCAAAAAACGAAAGGTGGCGCGCTCGTCGTGATCCACATGGTACTTTTCCATTAGCATAGCCATGGCTTCCATGCCTTTTTCCCAAGCTGAAACTAGGGGCTGGCTTGCCGGATTGCGAACAACAACAACATTCTTTTCATTTTCGTCGCTGTTTTGAGCGGAAGCCTTCAAAACGTGTACCTGCCTGGTTTCCATTTTTTGAAAAGACTGAGAACCGTCTGCATGATGCATAATGCGGACGGGCGGCATGCTGAAACCCATTACTCCTAAAACTGCTCTTGGTGGTTCTGCCTCTTCTTCTTCTGCACTCTCTGGGGAAAGAGGTATCGCAGCCATAGATTTCTTGACTTTTTTCTTTGGAGGTAAAGGCACAGCTTCCAGTTCTTCTTCGCTTTCGGAATCCAGAAAGTATCTGCCCATTTTTGGCGGCGGCGGCTGAGCGCTGCGGTCTGGGGTGCGCTCCCTCTGTGAGTGCTGATTGCTGGCCATTGCGGCCGCCTTGTACAGCTCGTCCATGCCGAGAGTGATCCCGGCGGCGGTCACGAACTCCAGCAGGACCATGTGATCGCGCTTCTCGTTGGGGTCTTTGCTCAGGGCGGACTGGGTGCTCAGGTAGTGGTTGTCGGGCAGCAGCACGGGGCCGTCGCCGATGGGGGTGTTCTGCTGGTAGTGGTCGGCGAGCTGCACGCTGCCGTCCTCGATGTTGTGGCGGATCTTGAAGTTCACCTTGATGCCGTTCTTCTGCTTGTCGGCCATGATATAGACGTTGTGGCTGTTGTAGTTGTACTCCAGCTTGTGCCCCAGGATGTTGCCGTCCTCCTTGAAGTCGATGCCCTTCAGCTCGATGCGGTTCACCAGGGTGTCGCCCTCGAACTTCACCTCGGCGCGGGTCTTGTAGTTGCCGTCGTCCTTGAAGAAGATGGTGCGCTCCTGGACGTAGCCTTCGGGCATGGCGGACTTGAAGAAGTCGTGCTGCTTCATGTGGTCGGGGTAGCGGCTGAAGCACTGCACGCCGTAGGTCAGGGTGGTCACGAGGGTGGGCCAGGGCACGGGCAGCTTGCCGGTGGTGCAGATGAACTTCAGGGTCAGCTTGCCGTAGGTGGCATCGCCCTCGCCCTCGCCGGACACGCTGAACTTGTGGCCGTTTACGTCGCCGTCCAGCTCGACCAGGATGGGCACCACCCCGGTGAACAGCTCCTCGCCCTTGCTCACCATTATTTAATCCTAGGCAAAGAAACACATGATGGATCTGGAGCCACAGGAAAGCTTAACCGCCCCCACCGCTCCCGCCATTGGCGCTACGGCTGTCATGGAGAAGGACAAAAGTCTACTCATACCCCAAGACGCACCGGTTGAGCAGAACTTGGGCTACGAGACTCCCCCCGAGGAATTTGAAGGCTTTCTTCAAATCCAAAAGCAACCAAATGAGCAAAACGCTGGGCTCGAGGACCATGACTACCTAAACGAGGGAGATGTCCTGTTTAAACATCTACAGCGACAAAGCACTATCGTTCGCGACGCCATATCTGATCGCTCTTCAATACCAGTTTCAATTGCAGAACTATCTTGCATCTACGAACGCAACCTGTTCTCCCCACGTGTGCCCCCTAAACGGCAAGCCAACGGCACATGCGAGCCAAATCCTCGCCTTAACTTCTACCCAGTTTTTGCAGTGCCAGAAGCACTGGCAACATACCATATTTTCTTTAAAAATCACAAAATACCCCTATCCTGTCGAGCTAACCGCAGCCGCGCAGATGAGCTTCTTGCTTTAAGGGCTGGCGCTTCCATACCTGGGATTGTGTCCTTGGAAGAGGTGCCTAAAATTTTTGAAGGTTTAGGTCGGGATGAAAAACGAGCAGCAAATGCCCTGCAAAAAGAAAATGAACAAAATCACCATGGGAATAGTGCTCTAATAGAACTGGAAGGTGACAATGCCCGCCTGGCAGTTTTAAAGCGCAATATTGAGGTTACTCACTTTGCCTACCCGGCAGTAAATCTTCCGCCAAAGGTAATGAGCGCAGTGATGAATCAGCTACTAATTAAGCGAGCCCAACCCATTGACAAAGATGCAAACTTGCAAGACCCGGAGGCAACAGATGATGGAAAGCCGGTTGTAAGCGACGAGCAATTAACTAAGTGGTTGGGAACAGACAATTCCAACGAACTACAACAGCGGCGTAAACTCATGATGGCCGCCGTACTTGTAACTGTGGAACTCGAGTGCATGCATCGTTTTTTCTCCGACATCACCACATTGCGCAAAATTGAGGAATGTCTTCACTACACTTTCCGCCATGGCTACGTGCGCCAAGCCTGTAAAATTTCTAATGTGGAGCTGAGCAATCTAGTTTCTTACATGGGCATCTTGCATGAAAACCGATTGGGACAGAACGTGCTACACTCAACACTACGCGATGAAGCACGCAGAGATTACGTGCGAGACTGCATTTACCTTTTCCTGTTACATACCTGGCAAACTGGGATGGGTGTTTGGCAGCAATGCTTGGAAGAAAAAAACCTTCGAGAACTAAACAAACTGTTAGACAGAGCACTAAAATCCCTATGGACCGGTTTTGACGAACGGACAGTAGCTGCAGAGCTAGCTGACATAATTTTCCCAGAAAGGTTAATGATAACCTTGCAAAACGGCTTGCCTGACTTTATGAGTCAAAGTATGCTGCACAATTATCGCTCTTTTATATTAGAGCGTTCTGGGATGCTTCCTAGCATGTGTTGTGCACTTCCTTCAGATTTTGTGCCTATATATTTTAGAGAGTGCCCCCCTCCCCTGTGGAGCCACTGCTACTTACTACGACTTGCTAACTACCTAGCTTACCACTCAGACCTTATGACAGATTCAAGCGGCGAAGGCCTAATGGAGTGTCACTGCCGCTGCAATCTTTGCACCCCCCACCGTTCTTTGGTTTGCAATACTGAACTATTAAGTGAAAGTCAAGTCATTGGTACCTTCGAAATGCAGGGACCGCAGTCTGACAGCAATTTCACGACGAACCTAAGACTTACCCCTGGGCTTTGGACTTCTGCCTACCTGCGCAAATTTGAACCCCAAGATTACCACGCCCACAGTATCAATTTTTACGAAGACCAATCCAAACCCCCAAAAGCGCCACTAACGGCTTGCGTCATTACGCAGGGAAAAATTCTAGCCCAATTGCATGCTATTAAGCAAGCGCGCGAAGAGTTTTTACTTAAAAAAGGACACGGAGTGTACCTTGATCCCCAAACCGGCGAGGAACTAAACCTTCCATCACCTTTGTGTGCTACTGCGTCTCCCCATTCGCAGCATGTCCCCGAAAGCCGCAAAACAGGCTATTGCGCAGCAACGCTCAAAGAAACAGCAGCAACGGCAGGAAATCTGGGAGGAAGAATCTTGGGAGAGTCAGGCAGAGGACGAGGTCGAGGACTTGGAAGAATGGGAGGAGGAGGAGGCGGACAGCCTAGACGAGGATCCAGAGGAGGAGGAGGAAGGTTCCAAGGACGGAGCGACCGCCGCAAAACCGTCGCTTTCAACCAAGCCCTCTCCAATGAAACCCGCTGTGAGCAAATCTCAGAAAGCCAGCCGTAGATGGGACACCATTGAAACCAGCGCCGCAAACTTGGGTAAGAATCGCAAGCAGGCGCGTCGGGGCTACTGCTCATGGCGGGCTCACAAAAGTAATATTGTAGCCTGCTTGCAGCACTGCGGGGGGAATATCTCATTTGCAAGGCGGTATTTGCTATACCATGATGGAGTGGCGATTCCAAGGAATGTCCTCCATTACTACCGTCATCTCTACAGCCCCTTTGAAGAGCTCGACAAGGAACCGACCTGCAACAGCCAAGCGGCCCACTAGAATCGGCAACAGCAGCAACAAGGAAAGTCCTGAGGCGCGCGAGTTAAGAAAACGCATTTTTCCCACTTTATATGCTATTTTTCAGCAGAGTCGAGGTCAAGAACACGAACTGAAAATAAAAAACCGTTCCCTGCGTTCACTTACCCGCAGCTGTCTCTACCTCAAAAGCGAAGATCAGTTGCAACGCACCTTGCAGGACGCAGAAGCTCTGTTCAATAAATACTGCTCCCTCTCGCTTAAAGAGTAAAAAAAGCCCGCGCGCGGACTTTCAACAGGCGGGAAAAGTGAATCGATATCTTTCTAGAAGATCTCCTACAATATTCTCAGCTGCCATGGAAAATCGATGTTCTTCTTTTATTCTCTCAAGATTTTCAGGCTGTATATTAAAACTTATATTAAGAACTATGCTAACCACCTCATCAGGAACCGTTGTAGGTGGCGTGGGTTTTCTTGGCAATCGACTCTCATGAAAACTACGAGCTAAATATTCAATATGTTCCTCTTGACCAACTTTATTCTGCATTTTTTTTGAACGAGGTTTAGAGCAAGCTTCAGGAAACTGAGACAGGAATTTTATTAAAAATTTAAATTTTGAAGAAAGTTCAGGGTTAATAGCATCCATTTTTTGCTTTGCAAGTTCCTCAGCATTCTTAACAAAAGACGTCTCTTTTGACATGTTTAAAGTTTAAACCTCCTGTGTGAAATTATTATCCGCTCATAATTCCACACATTATACGAGCCGGAAGCATAAAGTGTAAAGCCTGGGGTGCCTAATGAGTGAGCTAACTCACATTAA
