# Supplementary material for: AdamiForge: a modular reverse genetics tool for human adenovirus type 12 (HAdV-A12)
Source: Microbiol Spectr. 2026 May 21;14(7):e00696-26. doi: 10.1128/spectrum.00696-26 (PMC13340337; doi:10.1128/spectrum.00696-26)
Supplement: Reviewer comments [file reviewer-comments.pdf]

AMERICAN SOCIETY FOR MICROBIOLOGY | Microbiology Spectrum

AdamiForge: a modular reverse genetics tool for Human adenovirus type 12 (HAdV-A12)

maxence collard, jean-françois antoine, nicolas debortoli, céline maschietto, jonathan degosserie, Laurent Gillet, and Nicolas Gillet

Corresponding Author(s): Nicolas Gillet, Universite de Namur

Review Timeline:		
	Submission Date:	February 25, 2026
	Editorial Decision:	March 26, 2026
	Revision Received:	April 7, 2026
	Editorial Decision:	April 27, 2026
	Revision Received:	April 28, 2026
	Accepted:	April 29, 2026

Editor: Jose Martinez-Navio

Reviewer(s): The reviewers have opted to remain anonymous.

Transaction Report:

(Note: With the exception of the correction of typographical or spelling errors that could be a source of ambiguity, letters and reports are not edited. The original formatting of letters and referee reports may not be reflected in this compilation.)

DOI: https://doi.org/10.1128/spectrum.00696-26

Re: Spectrum00696-26 (AdamiForge: a modular reverse genetics tool for Human adenovirus type 12 (HAdV-A12))

Dear Prof. Nicolas Albert Gillet:

Thank you for the privilege of reviewing your work. Below you will find my comments, instructions from the Spectrum editorial office, and the reviewer comments.

Overall both reviewers have been very positive and their comments are more in the line of improving readability and clarity.

Please return the manuscript within 60 days; if you cannot complete the modification within this time period, please contact me. If you do not wish to modify the manuscript and prefer to submit it to another journal, notify me immediately so that the manuscript may be formally withdrawn from consideration by Spectrum.

Revision Guidelines
To submit your modified manuscript, log into the submission site at https://spectrum.msubmit.net/cgi-bin/main.plex. Go to Author Tasks and click the appropriate manuscript title to begin. The information you entered when you first submitted the paper will be displayed; update this as necessary. Note the following requirements:

• Upload point-by-point responses to the issues raised by the reviewers in a file named "Response to Reviewers," NOT in your cover letter.
• Upload a compare copy of the manuscript (without figures) as a "Marked-Up Manuscript" file.
• Upload a clean .DOC/.DOCX version of the revised manuscript and remove the previous version.
• Each figure must be uploaded as a separate, editable, high-resolution file (TIFF or EPS preferred), and any multipanel figures must be assembled into one file.
• Any supplemental material intended for posting by ASM should be uploaded with their legends separate from the main manuscript. You can combine all supplemental material into one file (preferred) or split it into a maximum of 10 files with all associated legends included.

For complete guidelines on revision requirements, see our Submission and Review Process webpage. Submission of a paper that does not conform to guidelines may delay acceptance of your manuscript.

Data availability: ASM policy requires that data be available to the public upon online posting of the article, so please verify all links to sequence records, if present, and make sure that each number retrieves the full record of the data. If a new accession number is not linked or a link is broken, provide Spectrum production staff with the correct URL for the record. If the accession numbers for new data are not publicly accessible before the expected online posting of the article, publication may be delayed; please contact production staff (Spectrum@asmusa.org) immediately with the expected release date.

Publication Fees: For information on publication fees and which article types are subject to charges, visit our website. If your manuscript is accepted for publication and any fees apply, you will be contacted separately about payment during the production process; please follow the instructions in that e-mail. Arrangements for payment must be made before your article is published.

ASM Membership: Corresponding authors may join or renew ASM membership to obtain discounts on publication fees. Need to upgrade your membership level? Please contact Customer Service at Service@asmusa.org.

The ASM Journals program strives for constant improvement in our submission and publication process. Please tell us how we can improve your experience by taking this quick Author Survey.

Thank you for submitting your paper to Spectrum.

Sincerely,
Jose Martinez-Navio
Editor
Microbiology Spectrum

Reviewer #1 (Comments for the Author):

The authors describe the construction and initial application of a new system to build recombinant HAdV-A12. This system is conceptually similar to the Adenobuilder system described for HAdV-C5.

A12 is probably the second best studied HAdV after C5, is quite divergent from C5 and A12 infection is actually tumorigenic in

select systems unlike C5. As such, having a modern ability to more readily generate select mutant virus in any gene is certainly going to be useful.

The manuscript is written at a low level with lots of experimental detail, which is essential for a technology based report. While not entirely perfect, the English is extremely good.

My only minor comment is related to the use of ClaI as the enzyme to release the genome fragments for the gibson assembly. ClaI is blocked by overlapping Dam methylation and it would be nice for the authors to remind the reader that in any future modifications for new construction, the user should avoid a flanking G or C that reconstitutes the GATC sequence recognized by the Dam methyltransferase in E. coli. I anticipate that pointing this out in the manuscript might save future uses some headaches.

Reviewer #2 (Comments for the Author):

This study presents AdamiForge, a modular reverse genetics platform developed for Human adenovirus type 12 (HAdV-A12). By adapting the AdenoBuilder system to accommodate its unique genome structure, the authors enable efficient assembly and targeted modification, including the generation of recombinant viruses expressing tagged or fluorescently labeled DNA-binding proteins. These tools facilitate detection in the absence of specific antibodies and generally have minimal impact on viral replication. Overall, the platform provides a valuable resource for advancing studies of HAdV-A12 biology and its applications.

Comments

1. Terminology: "Reverse Genetics"

The manuscript would benefit from a brief clarification of the term "reverse genetics," which is used throughout but not explicitly defined. While the meaning may be clear to readers familiar with contemporary usage, the term has historically been associated with RNA virus systems, and a short definition would improve clarity and accessibility for a broader audience.

2. Novelty and Significance

The manuscript describes the development of a reverse genetics tool for HAdV-A12 based on the AdenoBuilder system. While this is a valuable technical extension, the distinction between methodological innovation and adaptation could be articulated more clearly. In particular, it would strengthen the manuscript to more explicitly describe any challenges unique to HAdV-A12 and how these were addressed, thereby clarifying what aspects of the platform are novel beyond its application to this specific adenovirus type.

3. Methodological Strengths

The Materials and Methods section is well executed and includes several thoughtful adaptations for HAdV-A12. In particular, the use of A549 PIV5-V cells to suppress interferon signaling likely facilitates propagation of this more restrictive adenovirus type compared to commonly used strains such as HAdV-C5, and the genome segmentation strategy appears carefully designed for efficient assembly. The use of long-read sequencing to validate constructs further strengthens confidence in the system.

4. Genome Assembly Efficiency (Figure 3B)

In Figure 3B, the truncated genome products appear comparable in intensity to the full-length genome band, suggesting that a substantial proportion of assembly products may be incomplete. While full-length genomes are clearly obtained, it would be helpful to provide additional information on the efficiency of recovering correct assemblies (e.g., proportion of usable constructs or screening requirements) and whether truncated products impact downstream recovery of functional virus, as this has practical implications for reproducibility and adoption of the system.

5. Impact of DBP Tagging on Viral Behavior (Figure 4B)

The AdamiForge system is designed to preserve native viral genome organization, and the FLAG-tagged DBP construct appears to maintain replication kinetics comparable to wild-type virus (Figure 4B). However, the eGFP-tagged DBP exhibits delayed replication at early time points, indicating that not all modifications are functionally neutral. A more explicit discussion of how such tagging strategies may influence DBP function and viral replication would strengthen the manuscript, particularly for studies requiring minimally perturbed viral behavior.

6. Statistical Analysis of Replication Kinetics (Figure 4B)

It would be helpful to clarify whether statistical analyses were performed to assess differences in replication kinetics between variants, particularly for the observed delay in the eGFP-tagged virus.

7. Figure Readability (Figure 4B)

The font size in Figure 4B is difficult to read. Increasing the font size would improve clarity and accessibility for readers.

8. Framing of Future Applications in the Discussion

The Discussion outlines several potential applications of HAdV-A12, including its use in oncolytic vectors and the possibility of avoiding VITT based on capsid properties. These points are interesting and grounded in prior work; however, they are not directly addressed by the current study. It would strengthen the manuscript to more clearly distinguish between conclusions supported by the presented data and forward-looking hypotheses, to ensure that the scope of the findings is clearly defined.

Resubmission for Spectrum00696-26 (AdamiForge: a modular reverse genetics tool for Human adenovirus type 12 (HAdV-A12))

Dear Dr Martinez-Navio, dear reviewers,

First, we would like to thank you for considering our work and appreciate the time you spent reviewing our manuscript. Your requests and comments have contributed to the improvement of the manuscript.

This letter contains a detailed list of our responses (colored in blue in the following text) and a description of the changes we have made to the manuscript.

Reviewer #1 (Comments for the Author):

The authors describe the construction and initial application of a new system to build recombinant HAdV-A12. This system is conceptually similar to the Adenobuilder system described for HAdV-C5.

A12 is probably the second best studied HAdV after C5, is quite divergent from C5 and A12 infection is actually tumorigenic in select systems unlike C5. As such, having a modern ability to more readily generate select mutant virus in any gene is certainly going to be useful.

The manuscript is written at a low level with lots of experimental detail, which is essential for a technology based report. While not entirely perfect, the English is extremely good.

My only minor comment is related to the use of ClaI as the enzyme to release the genome fragments for the gibson assembly. ClaI is blocked by overlapping Dam methylation and it would be nice for the authors to remind the reader that in any future modifications for new construction, the user should avoid a flanking G or C that reconstitutes the GATC sequence recognized by the Dam methyltransferase in E. coli. I anticipate that pointing this out in the manuscript might save future uses some headaches.

We agree with the reviewer and have added the following sentence to the manuscript in the first paragraph of the results section (overview of AdamiForge):
"Importantly, ClaI is blocked by overlapping dam methylation. If future users wish to modify the plasmids, they must ensure that no dam methylation site (GATC) overlaps the ClaI restriction site (ATCGAT). Therefore, it is imperative to avoid introducing a G or C flanking the ClaI site."

Reviewer #2 (Comments for the Author):

This study presents AdamiForge, a modular reverse genetics platform developed for Human adenovirus type 12 (HAdV-A12). By adapting the AdenoBuilder system to accommodate its unique genome structure, the authors enable efficient assembly and targeted modification, including the generation of recombinant viruses expressing tagged or fluorescently labeled DNA-binding proteins. These tools facilitate detection

in the absence of specific antibodies and generally have minimal impact on viral replication. Overall, the platform provides a valuable resource for advancing studies of HAdV-A12 biology and its applications.

Comments

1. Terminology: "Reverse Genetics"

The manuscript would benefit from a brief clarification of the term "reverse genetics," which is used throughout but not explicitly defined. While the meaning may be clear to readers familiar with contemporary usage, the term has historically been associated with RNA virus systems, and a short definition would improve clarity and accessibility for a broader audience.

We clarified the term “reverse genetics” by the addition of following sentences in the introduction section:
A reverse genetics tool is an experimental system that enables the reconstruction of viral particles from a known viral genome sequence. The genome, cloned into a plasmid, can be deliberately modified through mutations, deletions, or insertions. This approach is termed “reverse genetics” because it begins with defined genetic changes and examines the resulting phenotype.

2. Novelty and Significance

The manuscript describes the development of a reverse genetics tool for HAdV-A12 based on the AdenoBuilder system. While this is a valuable technical extension, the distinction between methodological innovation and adaptation could be articulated more clearly. In particular, it would strengthen the manuscript to more explicitly describe any challenges unique to HAdV-A12 and how these were addressed, thereby clarifying what aspects of the platform are novel beyond its application to this specific adenovirus type.

It would be fair to say that AdamiForge is a technical adaptation of the AdenoBuilder system for HAdV-A12.

A key feature distinguishing HAdV-A12 from C5 is its relatively poor propagation in A549 cells. This likely reflects the fact that A12 encodes only a single VA-RNA, resulting in less efficient antagonism of PKR activation and interferon signaling compared to the C5 strain. Consequently, following the initial recovery of infectious viral particles in 293T cells, viral stocks are amplified through multiple passages in interferon-deficient A549 cells (A549 PIV5-V - engineered to constitutively express the V protein of parainfluenza virus type 5).

We added the following sentence in the “Block release, genome reassembly and production of viral particles” of the results section:
“After recovery of the first virions produced in HEK-293T cells, successive rounds of amplification in A549 cells deficient for IFN signaling (like A549 PIV5-V) will be necessary to achieve a high-titer production.”

3. Methodological Strengths

The Materials and Methods section is well executed and includes several thoughtful adaptations for HAdV-A12. In particular, the use of A549 PIV5-V cells to suppress interferon signaling likely facilitates propagation of this more restrictive adenovirus type compared to commonly used strains such as HAdV-C5, and the genome segmentation strategy appears carefully designed for efficient assembly. The use of long-read sequencing to validate constructs further strengthens confidence in the system.

4. Genome Assembly Efficiency (Figure 3B)

In Figure 3B, the truncated genome products appear comparable in intensity to the full-length genome band, suggesting that a substantial proportion of assembly products may be incomplete. While full-length genomes are clearly obtained, it would be helpful to provide additional information on the efficiency of recovering correct assemblies (e.g., proportion of usable constructs or screening requirements) and whether truncated products impact downstream recovery of functional virus, as this has practical implications for reproducibility and adoption of the system.

Thank you for this important comment.

It is true that a significant proportion of the material that is transfected into 293T cells is made of incompletely reconstituted viral genome. We did not investigate whether partial reassemblies negatively affected the recovery of functional viruses.

One possible approach would be to excise the full-length genome from the gel prior to purification and to transfect only the purified full-length product. However, this additional step would increase the duration of the procedure, as separation of such large DNA fragments requires overnight electrophoresis. Transfection of the total mixture, including recombination by-products, simplifies the workflow and we always managed to recover infectious particles.

Importantly, during reconstitution of the GFP-expressing virus, a high proportion of 293T cells appeared GFP-positive following transfection with the reassembly mix, which could suggest widespread viral replication. However, upon the first passage in A549 cells, only a small number of cells exhibited GFP expression. This indicates that only a limited fraction of the initially GFP-positive 293T cells actually harbored a fully recombined, replication-competent viral genome.

In the material and methods section, we added the following sentences:
“If using the eGFP-DPB construct, the user should observe a fair proportion of GFP-positive 293T cells after transfection of the reassembly mix. Despite that, the first passage in A549 PIV5-V cells would only lead to very few replication plaques. This is expected and suggests that only a limited fraction of the initially GFP-positive 293T cells actually harbored a fully recombined, replication-competent viral genome. It is reasonable to assume that such a bottleneck is also present for other constructions (although less easily visible) and that therefore several amplification cycles in the A549 PIV5-V cells are required to achieve high-titer production.”

5. Impact of DBP Tagging on Viral Behavior (Figure 4B)

The AdamiForge system is designed to preserve native viral genome organization, and the FLAG-tagged DBP construct appears to maintain replication kinetics comparable to wild-type virus (Figure 4B). However, the eGFP-tagged DBP exhibits delayed replication at early time points, indicating that not all modifications are functionally neutral. A more explicit discussion of how such tagging strategies may influence DBP function and viral replication would strengthen the manuscript, particularly for studies requiring minimally perturbed viral behavior.

We added the following sentence in the “Creation of modified HAdV-A12 using the AdamiForge system” of the results section:
“This delayed replication for AF eGFP-DBP shows that not all modifications are functionally neutral. Fusion of the 27 kDA eGFP protein to DBP reduces replication kinetics, consistent with previous description for the HAdV-C5 strain.”

We added the following sentence in the Discussion section:
“The AF eGFP-DBP allows the visualization of viral replication centers (VRCs) in infected cells without using standard immunostaining methods. The morphology of DBP evolves during the infection, appearing as small nuclear foci in the early stages, then developing into larger structures, and finally concentrating at the nuclear periphery in the later stages. Because AF eGFP-DBP displays a slight delay in replication, the use of AF 3xFLAG-DBP would be more suitable for studies requiring minimally perturbed viral behavior.”

6. Statistical Analysis of Replication Kinetics (Figure 4B)

It would be helpful to clarify whether statistical analyses were performed to assess differences in replication kinetics between variants, particularly for the observed delay in the eGFP-tagged virus.

T-tests were performed for each time points and results were added to Figure 4B.

7. Figure Readability (Figure 4B)

The font size in Figure 4B is difficult to read. Increasing the font size would improve clarity and accessibility for readers.

The modifications on Figure 4B have been made accordingly.

8. Framing of Future Applications in the Discussion

The Discussion outlines several potential applications of HAdV-A12, including its use in oncolytic vectors and the possibility of avoiding VITT based on capsid properties. These points are interesting and grounded in prior work; however, they are not directly addressed by the current study. It would strengthen the manuscript to more clearly distinguish between conclusions supported by the presented data and forward-looking hypotheses, to ensure that the scope of the findings is clearly defined.

We rephrased the end of the paragraph relative to the VITT issue to distinguish more clearly our results from possible future applications:

"Since then, the scientific community has devoted considerable effort to addressing this issue, mainly by selecting adenoviral capsids that do not bind PF4. Interestingly, the HAdV-A12 capsid does not seem to bind PF4, suggesting that an A12-based vector should not elicit VITT."

Re: Spectrum00696-26R1 (AdamiForge: a modular reverse genetics tool for Human adenovirus type 12 (HAdV-A12))

Dear Prof. Nicolas Albert Gillet:

Thank you for the privilege of reviewing your work. Below you will find my comments, instructions from the Spectrum editorial office, and the reviewer comments.

It seems one of the reviewers would like additional clarifications on a few points. Please return the manuscript within 60 days; if you cannot complete the modification within this time period, please contact me. If you do not wish to modify the manuscript and prefer to submit it to another journal, notify me immediately so that the manuscript may be formally withdrawn from consideration by Spectrum.

Revision Guidelines
To submit your modified manuscript, log into the submission site at https://spectrum.msubmit.net/cgi-bin/main.plex. Go to Author Tasks and click the appropriate manuscript title to begin. The information you entered when you first submitted the paper will be displayed; update this as necessary. Note the following requirements:

• Upload point-by-point responses to the issues raised by the reviewers in a file named "Response to Reviewers," NOT in your cover letter.
• Upload a compare copy of the manuscript (without figures) as a "Marked-Up Manuscript" file.
• Upload a clean .DOC/.DOCX version of the revised manuscript and remove the previous version.
• Each figure must be uploaded as a separate, editable, high-resolution file (TIFF or EPS preferred), and any multipanel figures must be assembled into one file.
• Any supplemental material intended for posting by ASM should be uploaded with their legends separate from the main manuscript. You can combine all supplemental material into one file (preferred) or split it into a maximum of 10 files with all associated legends included.

For complete guidelines on revision requirements, see our Submission and Review Process webpage. Submission of a paper that does not conform to guidelines may delay acceptance of your manuscript.

Data availability: ASM policy requires that data be available to the public upon online posting of the article, so please verify all links to sequence records, if present, and make sure that each number retrieves the full record of the data. If a new accession number is not linked or a link is broken, provide Spectrum production staff with the correct URL for the record. If the accession numbers for new data are not publicly accessible before the expected online posting of the article, publication may be delayed; please contact production staff (Spectrum@asmusa.org) immediately with the expected release date.

Publication Fees: For information on publication fees and which article types are subject to charges, visit our website. If your manuscript is accepted for publication and any fees apply, you will be contacted separately about payment during the production process; please follow the instructions in that e-mail. Arrangements for payment must be made before your article is published.

ASM Membership: Corresponding authors may join or renew ASM membership to obtain discounts on publication fees. Need to upgrade your membership level? Please contact Customer Service at Service@asmusa.org.

The ASM Journals program strives for constant improvement in our submission and publication process. Please tell us how we can improve your experience by taking this quick Author Survey.

Thank you for submitting your paper to Spectrum.

Sincerely,
Jose Martinez-Navio
Editor
Microbiology Spectrum

Reviewer #1 (Comments for the Author):

I think that the authors have addressed my single comment as well as the suggestions of the other reviewer appropriately. I am happy with the final manuscript.

Reviewer #2 (Comments for the Author):

Several key concerns remain only partially addressed. The manuscript still does not provide quantitative information on genome assembly efficiency (Comment #4), including recovery of full-length genomes and screening requirements. It is not clear what is new here versus what has been adapted from existing approaches (Comment #2). In addition, elements of the Discussion continue to extend beyond the data presented without clear separation from supported conclusions (Comment #8).

Resubmission for Spectrum00696- 26R1 (AdamiForge: a modular reverse genetics tool for Human adenovirus type 12 (HAdV-A12))

Dear Dr Martinez-Navio, dear reviewers,

First, we would like to thank you again for the time you spent reviewing our manuscript. This letter contains a detailed list of our responses (colored in blue in the following text) and a description of the changes we have made to the manuscript.

Reviewer #1 (Comments for the Author):

I think that the authors have addressed my single comment as well as the suggestions of the other reviewer appropriately. I am happy with the final manuscript.

Reviewer #2 (Comments for the Author):

Several key concerns remain only partially addressed.

The manuscript still does not provide quantitative information on genome assembly efficiency (Comment #4), including recovery of full-length genomes and screening requirements.

Upon recombination, a significant proportion of the material transfected into 293T cells consists of incompletely reconstituted viral genomes. Estimating this proportion from figure 3B is rather unreliable, as band intensity depends not only on quantity but also on the length of the recombined fragments. For this reason, we did not include a figure for this proportion. Importantly, no screening step is required, as only correctly reassembled genomes can replicate in A549 cells. Upon recovery of the recombinant virus, high-throughput sequencing is recommended to verify the integrity of the construct.

In the material and methods section, we added the following sentences:
"No screening step is required, the first passage in A549 PIV5-V acts as a selection step, only retaining fully recombined, replication-competent viral genomes."
"Upon recovery of the recombinant virus, viral genome sequencing is recommended to verify the integrity of the construct."

It is not clear what is new here versus what has been adapted from existing approaches (Comment #2).

The principle of AdamiForge is not new, we have adapted the modular AdenoBuilder system to the A12 strain. To clarify that point, we added the following sentence in the introduction:
"The concept underlying AdamiForge is not novel; rather, it represents an adaptation of the modular AdenoBuilder system to the HAdV-A12 strain."

In addition, elements of the Discussion continue to extend beyond the data presented without clear separation from supported conclusions (Comment #8).

We reorganized the discussion to establish a clear separation between our tool and the data presented and the possible future applications. We split the discussion into four paragraphs.

The first paragraph compares AdamiForge to other reverse genetics tools for adenoviruses.

The second paragraph details the different mutated viruses constructed in this paper.

The third paragraph presents future applications made possible with AdamiForge. To separate presented data a from forward-looking hypothesis, we started this paragraph with the following sentence: “Although it is not within the scope of this article, AdamiForge tool could be instrumental for extending our knowledge on HAdV-A12 and for the design of A12-based therapeutics viruses and vectors.”

The fourth paragraph resumes the properties of AdamiFroge tool and ends the discussion section.

Re: Spectrum00696-26R2 (AdamiForge: a modular reverse genetics tool for Human adenovirus type 12 (HAdV-A12))

Dear Prof. Nicolas Albert Gillet:

Congratulations!

Your manuscript has been accepted, and I am forwarding it to the ASM production staff for publication. Your paper will first be checked to make sure all elements meet the technical requirements. ASM staff will contact you if anything needs to be revised before copyediting and production can begin. Otherwise, you will be notified when your proofs are ready to be viewed.

Data Availability: ASM policy requires that data be available to the public upon online posting of the article, so please verify all links to sequence records, if present, and make sure that each number retrieves the full record of the data. If a new accession number is not linked or a link is broken, provide production staff with the correct URL for the record. If the accession numbers for new data are not publicly accessible before the expected online posting of the article, publication may be delayed; please contact ASM production staff immediately with the expected release date.

Publication Fees: For information on publication fees and which article types have charges, please visit our website. We have partnered with Copyright Clearance Center (CCC) to collect author charges. If fees apply to your paper, you will receive a message from no-reply@copyright.com with further instructions. For questions related to paying charges through RightsLink, please contact CCC at ASM_Support@copyright.com or toll free at +1-877-622-5543. CCC makes every attempt to respond to all emails within 24 hours.

ASM Membership: Corresponding authors may join or renew ASM membership to obtain discounts on publication fees. Need to upgrade your membership level? Please contact Customer Service at Service@asmusa.org.

Embargo Policy: A press release may be issued as soon as the manuscript is posted on the Spectrum Latest Articles webpage. The corresponding author will receive an email with the subject line "ASM Journals Author Services Notification" when the article is available online.

The ASM Journals program strives for constant improvement in our submission and publication process. Please tell us how we can improve your experience by taking this quick Author Survey.

Thank you for submitting your paper to Spectrum.

Sincerely,
Jose Martinez-Navio
Editor
Microbiology Spectrum
